# Supplementary material for: In-depth study of tomato and weed viromes reveals undiscovered plant virus diversity in an agroecosystem
Source: Microbiome. 2023 Mar 28;11:60. doi: 10.1186/s40168-023-01500-6 (PMC10042675; doi:10.1186/s40168-023-01500-6)
Supplement: Supplementary file 4 — Additional file 3: Supplementary Figure 1. Genome organization of novel viruses, first full genomes of known viruses showing known and putative open reading frames and the protein they code for and predicted secondary structures of selected viroid-like circular RNAs detected in this study. [file 40168_2023_1500_MOESM3_ESM.pdf]

In-depth study of tomato and weed viromes reveals undiscovered plant virus diversity in an agroecosystem

Rivarez, MPS[1,2,*], Pecman, A[1,2], Bačnik, K[1,2], Maksimović Carvalho Ferreira, O[1,2], Vučurović, A[1], Seljak, G[1], Mehle, N[1,3], Gutiérrez-Aguirre, I[1], Ravnikar, M[1], and Kutnjak, D[1,*]

[1]Department of Biotechnology and Systems Biology, National Institute of Biology, Večna pot 111, Ljubljana, 1000 Slovenia. [2]Jožef Stefan International Postgraduate School, Jamova cesta 39, Ljubljana, 1000 Slovenia. [3]School for Viticulture and Enology, University of Nova Gorica, Dvorec Lanthieri Glavni trg 8, Vipava, 5271 Slovenia. *for correspondence email Denis Kutnjak (denis.kutnjak@nib.si) and Mark Paul Selda Rivarez (mpsrivarez@gmail.com)

SUPPLEMENTARY INFORMATION

Additonal File 03

Supplementary Figure 1. Genome organization of novel viruses or first full genomes of known viruses, showing known and putative open reading frames and the protein it codes for, and predicted secondary structures of selected viroid-like circular RNAs detected in this study.

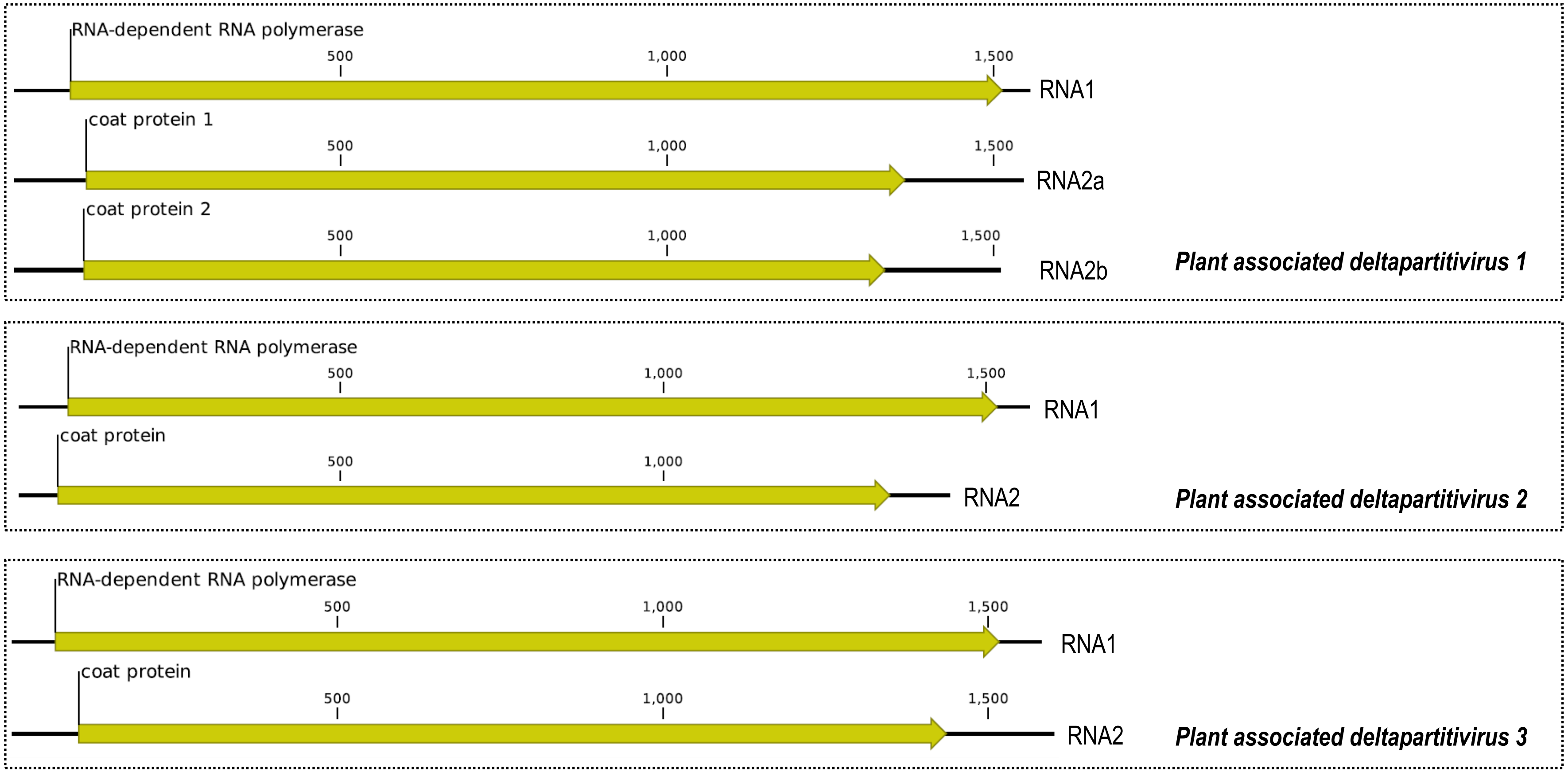

Supplementary Figure 1-01. Genomes of new virus species discovered under family *Partitiviridae,* order *Durnavirales.*

Supplementary Figure 1. Genome organization of novel viruses or first full genomes of known viruses, showing known and putative open reading frames and the protein it codes for, and predicted secondary structures of selected viroid-like circular RNAs detected in this study. **Note**: Genome length in number of bases are shown with a scale. For full information on genome length, protein domains, *etc.,* please refer to Supplementary Table 5, and the corresponding accession in GenBank.

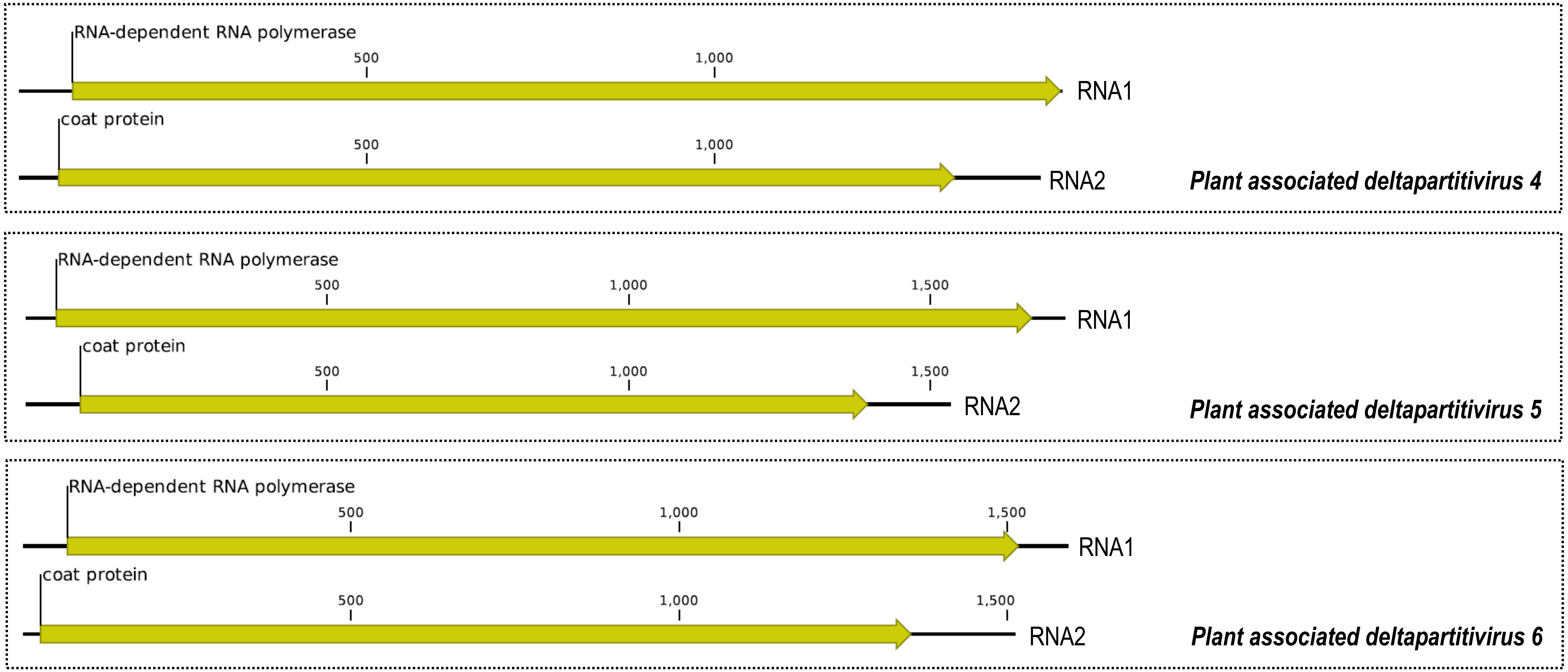

Supplementary Figure 1-01 (continued)**.** Genomes of new virus species discovered under family *Partitiviridae,* order *Durnavirales.*

Supplementary Figure 1 (continued)**.** Genome organization of novel viruses or first full genomes of known viruses, showing known and putative open reading frames and the protein it codes for, and predicted secondary structures of selected viroid-like circular RNAs detected in this study. **Note**: Genome length in number of bases are shown with a scale. For full information on genome length, protein domains, *etc.,* please refer to Supplementary Table 5, and the corresponding accession in GenBank.

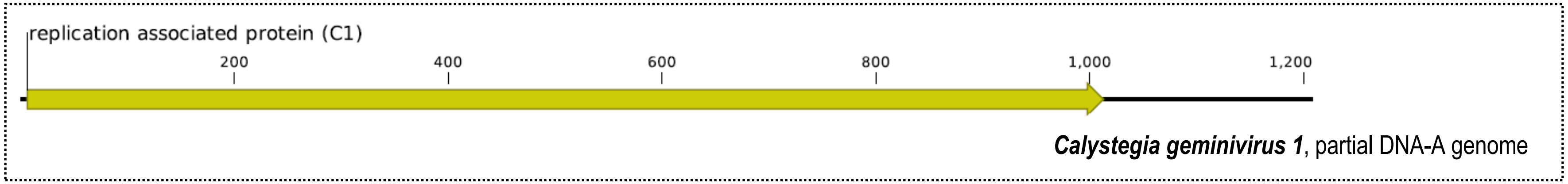

Supplementary Figure 1-02. Genomes of new virus species discovered under *Geminiviridae* (order *Geplafuvirales*).

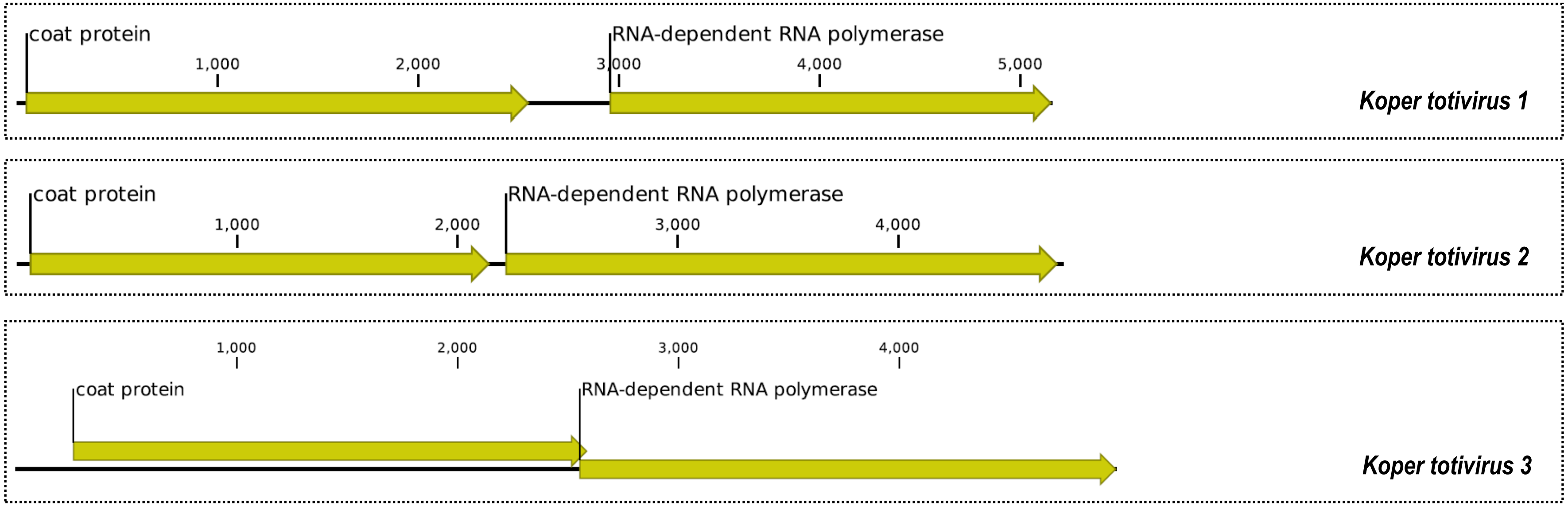

Supplementary Figure 1-03. Genomes of new virus species discovered under *Totiviridae* (order *Ghabrivirales*).

Supplementary Figure 1 (continued)**.** Genome organization of novel viruses or first full genomes of known viruses, showing known and putative open reading frames and the protein it codes for, and predicted secondary structures of selected viroid-like circular RNAs detected in this study. **Note**: Genome length in number of bases are shown with a scale. For full information on genome length, protein domains, *etc.,* please refer to Supplementary Table 5, and the corresponding accession in GenBank.

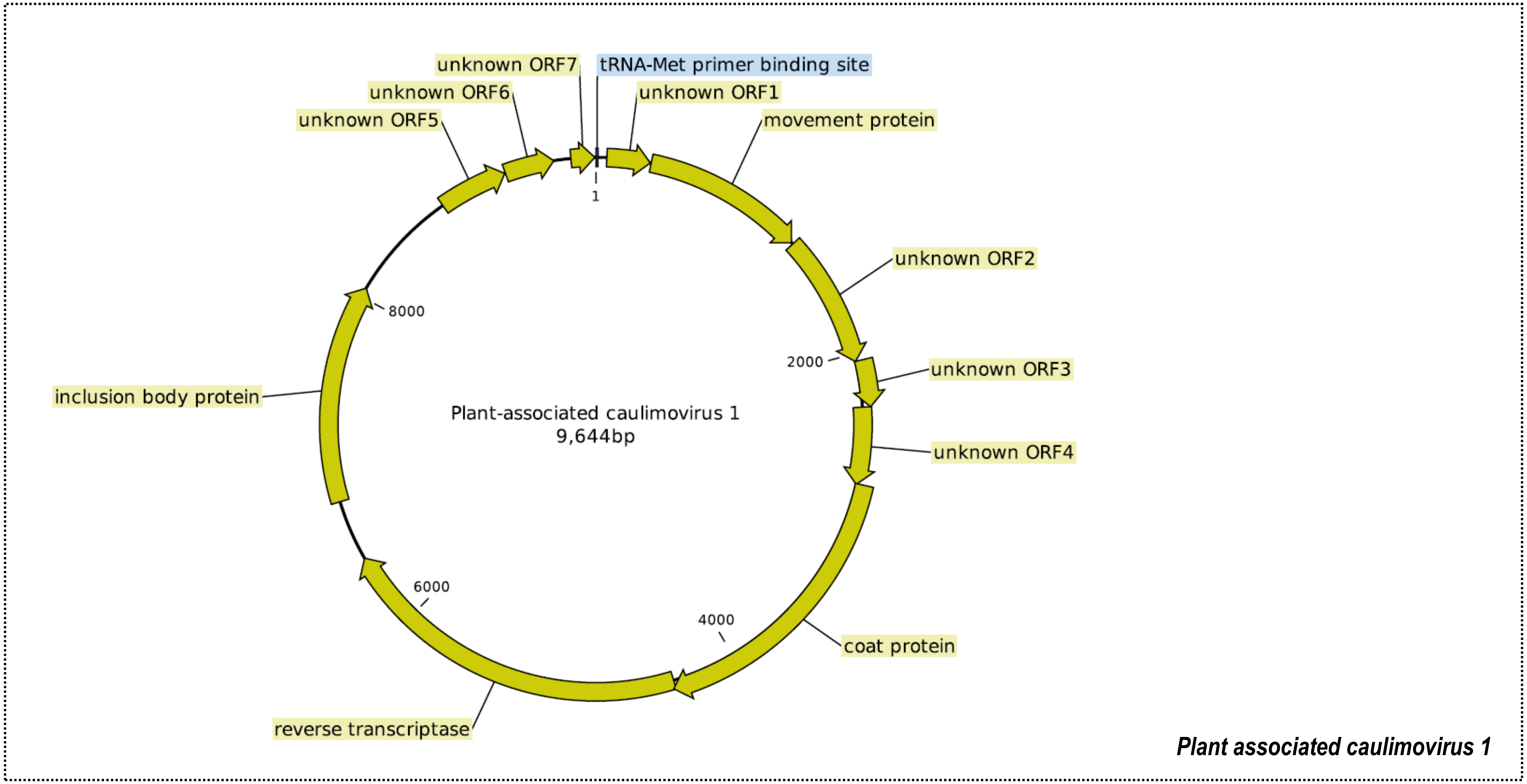

Supplementary Figure 1-04. Genomes of new virus species discovered under family *Caulimoviridae* (order *Ortelivirales*).

Supplementary Figure 1 (continued). Genome organization of novel viruses or first full genomes of known viruses, showing known and putative open reading frames and the protein it codes for, and predicted secondary structures of selected viroid-like circular RNAs detected in this study. **Note**: Genome length in number of bases are shown with a scale. For full information on genome length, protein domains, *etc.,* please refer to Supplementary Table 5, and the corresponding accession in GenBank.

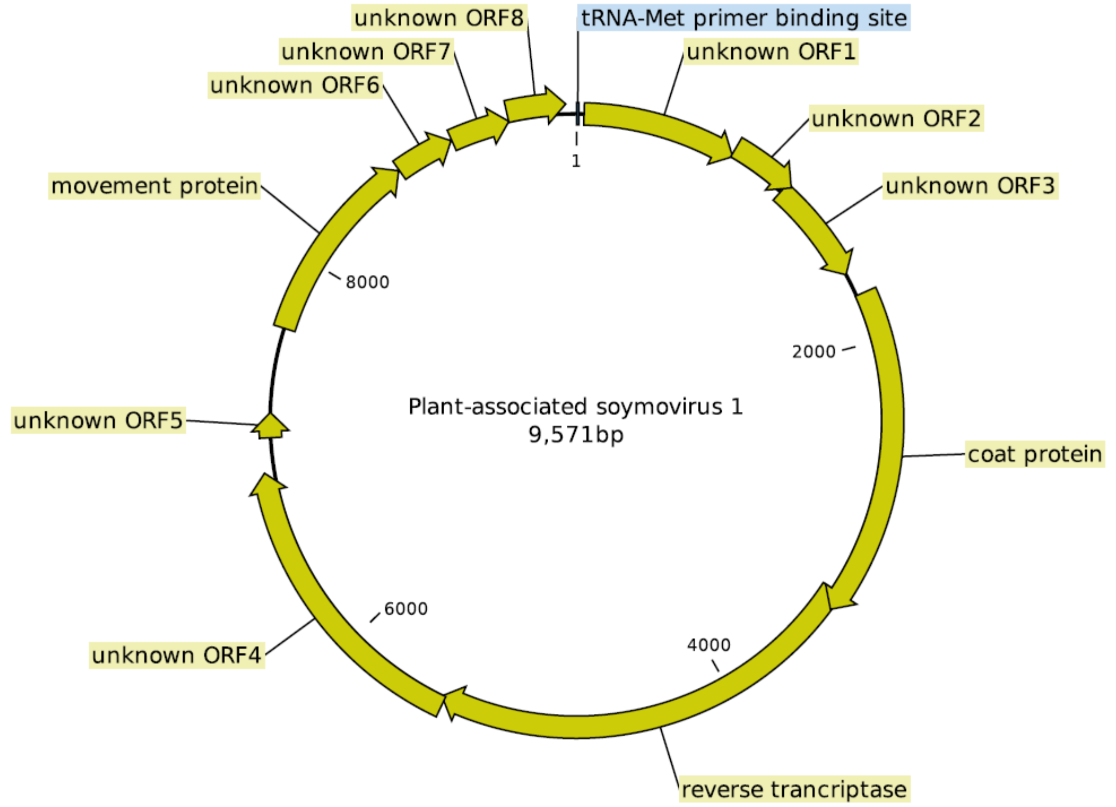

Plant associated soymovirus 1

Supplementary Figure 1-04 (continued)**.** Genomes of new virus species discovered under family *Caulimoviridae* (order *Ortelivirales*).

Supplementary Figure 1 (continued)**.** Genome organization of novel viruses or first full genomes of known viruses, showing known and putative open reading frames and the protein it codes for, and predicted secondary structures of selected viroid-like circular RNAs detected in this study. **Note**: Genome length in number of bases are shown with a scale. For full information on genome length, protein domains, *etc.,* please refer to Supplementary Table 5, and the corresponding accession in GenBank.

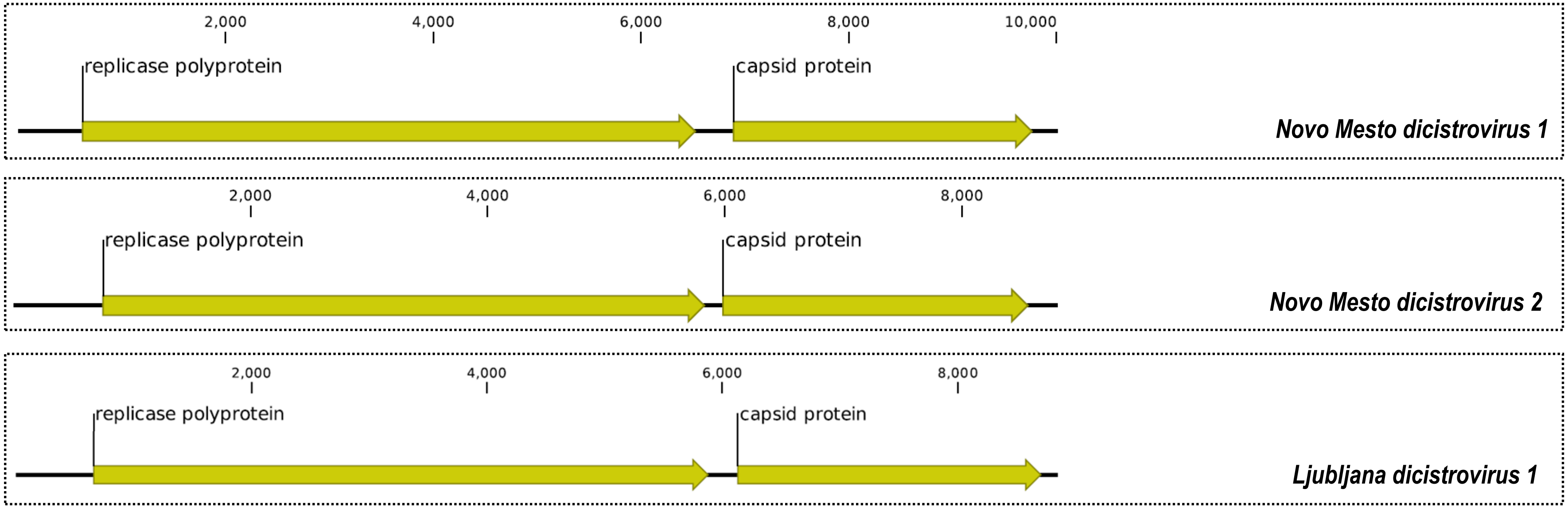

Supplementary Figure 1-05. Genomes of new virus species discovered under family *Dicistroviridae* (order *Picornavirales*).

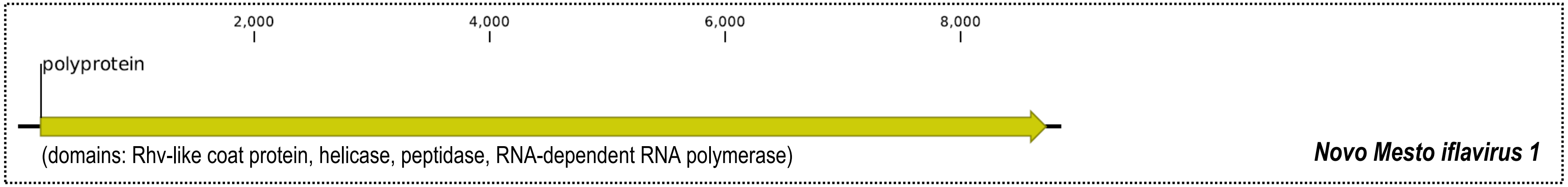

Supplementary Figure 1-06. Genomes of new virus species discovered under family *Iflaviridae* (order *Picornavirales*).

Supplementary Figure 1 (continued)**.** Genome organization of novel viruses or first full genomes of known viruses, showing known and putative open reading frames and the protein it codes for, and predicted secondary structures of selected viroid-like circular RNAs detected in this study. **Note**: Genome length in number of bases are shown with a scale. For full information on genome length, protein domains, *etc.,* please refer to Supplementary Table 5, and the corresponding accession in GenBank.

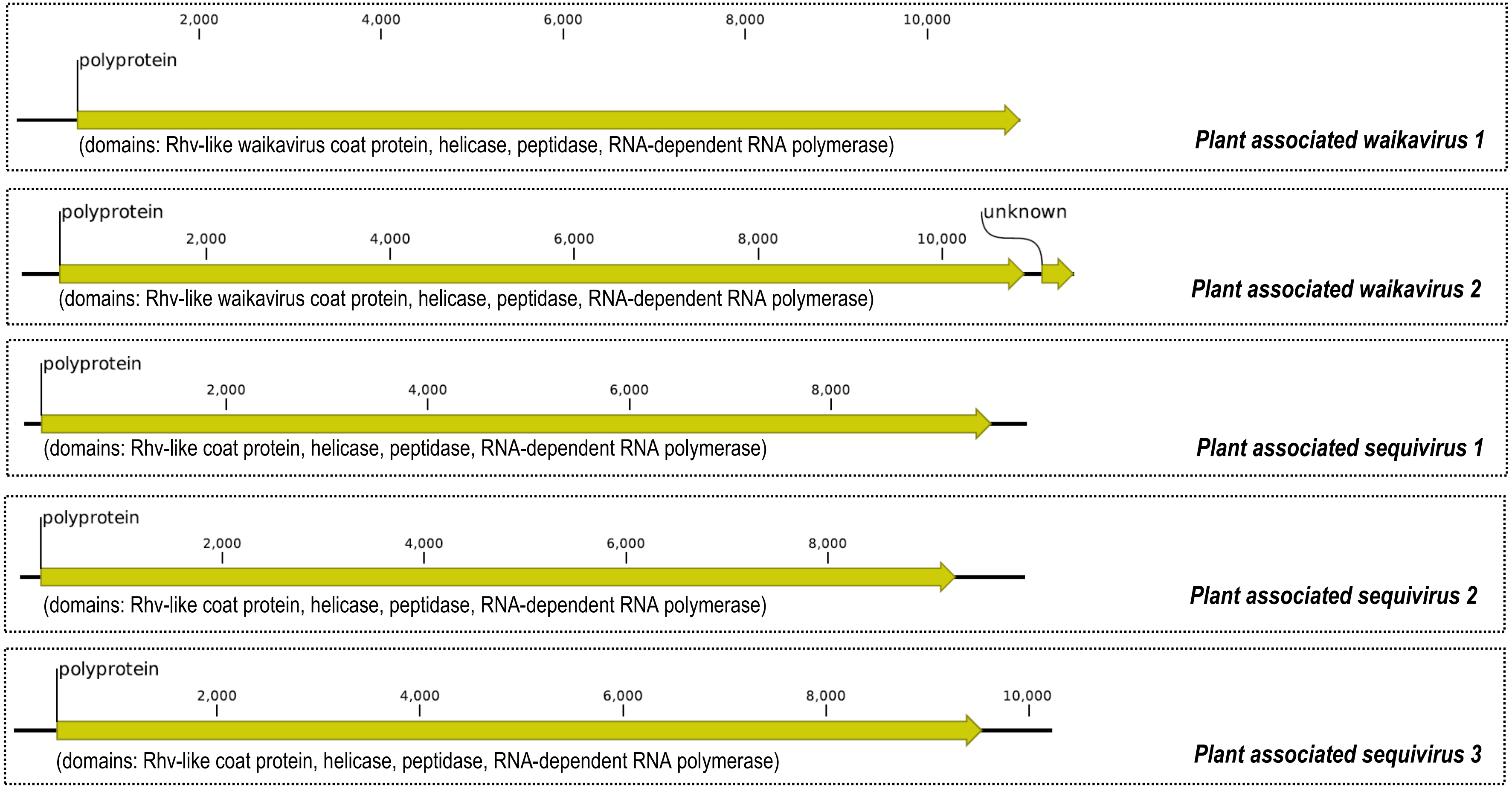

Supplementary Figure 1-07. Genomes of new virus species discovered under family *Secoviridae* (order *Picornavirales*).

Supplementary Figure 1 (continued)**.** Genome organization of novel viruses or first full genomes of known viruses, showing known and putative open reading frames and the protein it codes for, and predicted secondary structures of selected viroid-like circular RNAs detected in this study. **Note**: Genome length in number of bases are shown with a scale. For full information on genome length, protein domains, *etc.,* please refer to Supplementary Table 5, and the corresponding accession in GenBank.

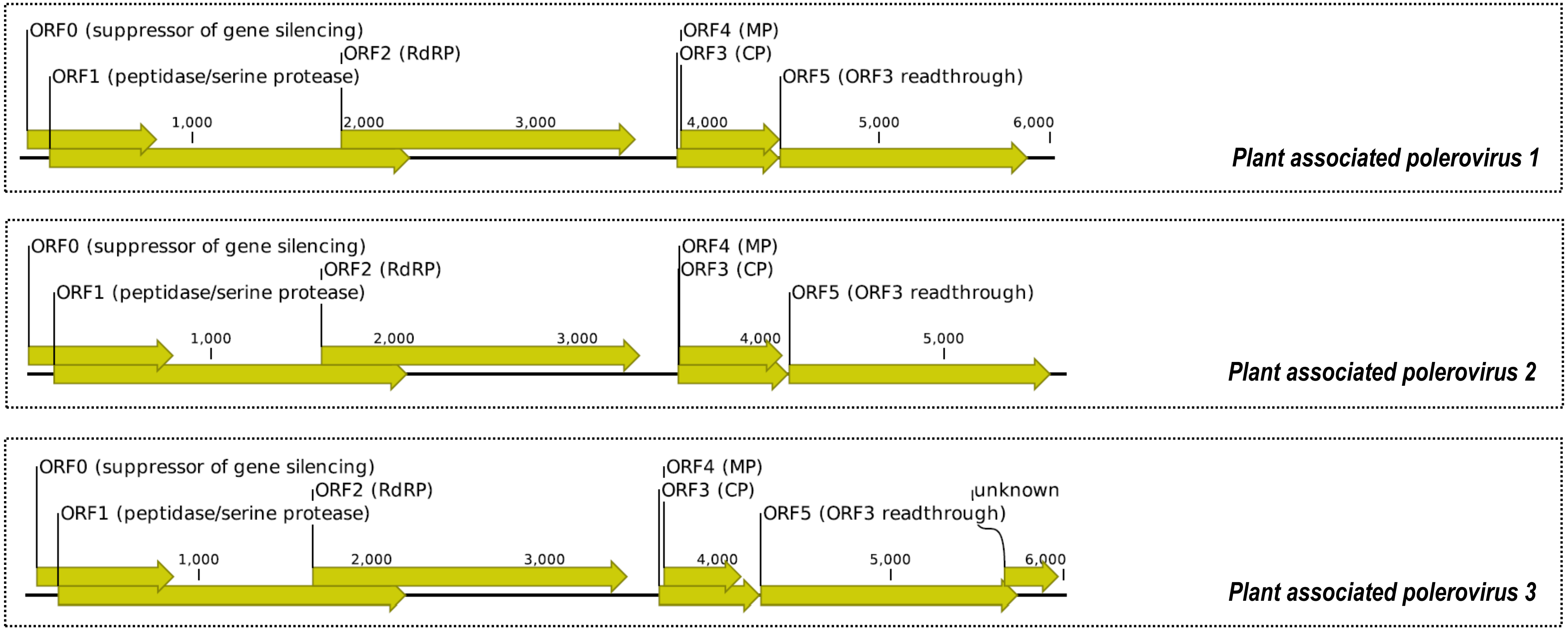

Supplementary Figure 1-08. Genomes of new virus species discovered under genus *Polerovirus,* family *Solemoviridae* (order *Sobelivirales*)*.* **Note:** RdRP - RNA-dependent RNA polymerase, MP - movement protein, CP - coat protein.

Supplementary Figure 1 (continued)**.** Genome organization of novel viruses or first full genomes of known viruses, showing known and putative open reading frames and the protein it codes for, and predicted secondary structures of selected viroid-like circular RNAs detected in this study. **Note**: Genome length in number of bases are shown with a scale. For full information on genome length, protein domains, *etc.,* please refer to Supplementary Table 5, and the corresponding accession in GenBank.

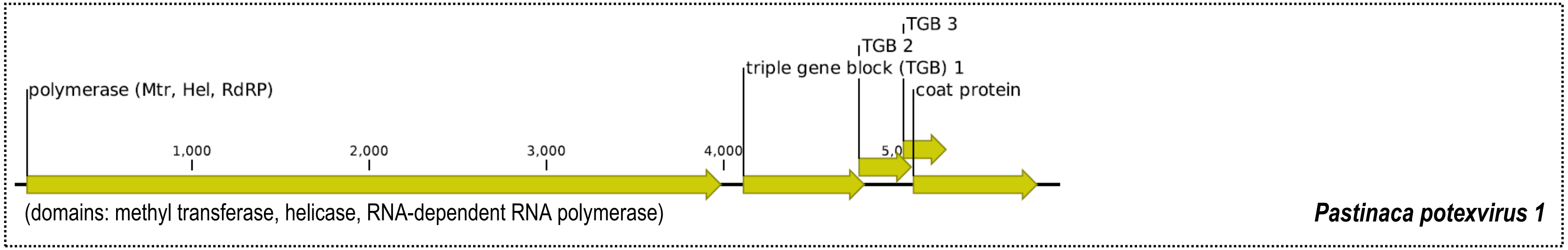

Supplementary Figure 1-09. Genome of new virus species discovered under genus *Potexvirus,* family *Alphaflexiviridae* (order *Tymovirales*).

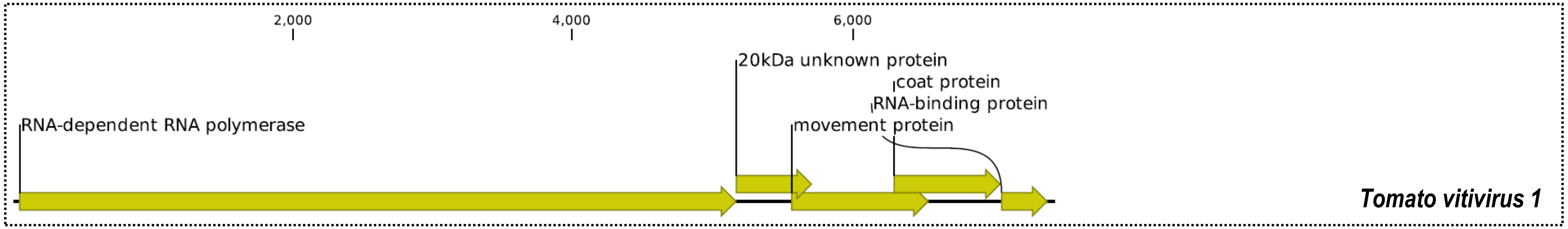

Supplementary Figure 1-10. Genome of new virus species discovered under genus *Vitivirus,* family *Betaflexiviridae* (order *Tymovirales*).

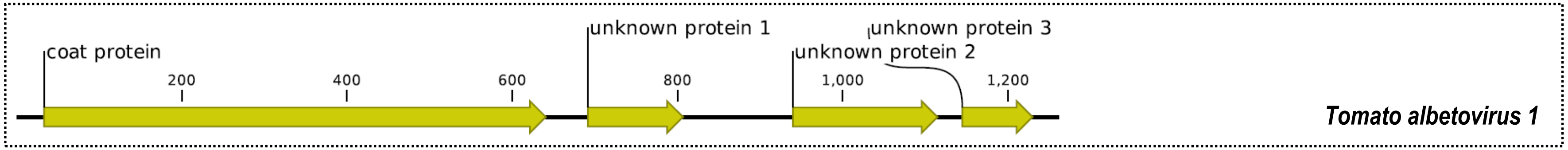

Supplementary Figure 1-11. Genome of new virus species discovered under genus *Albetovirus,* under unclassified satellite viruses.

Supplementary Figure 1 (continued)**.** Genome organization of novel viruses or first full genomes of known viruses, showing known and putative open reading frames and the protein it codes for, and predicted secondary structures of selected viroid-like circular RNAs detected in this study. **Note**: Genome length in number of bases are shown with a scale. For full information on genome length, protein domains, *etc.,* please refer to Supplementary Table 5, and the corresponding accession in GenBank.

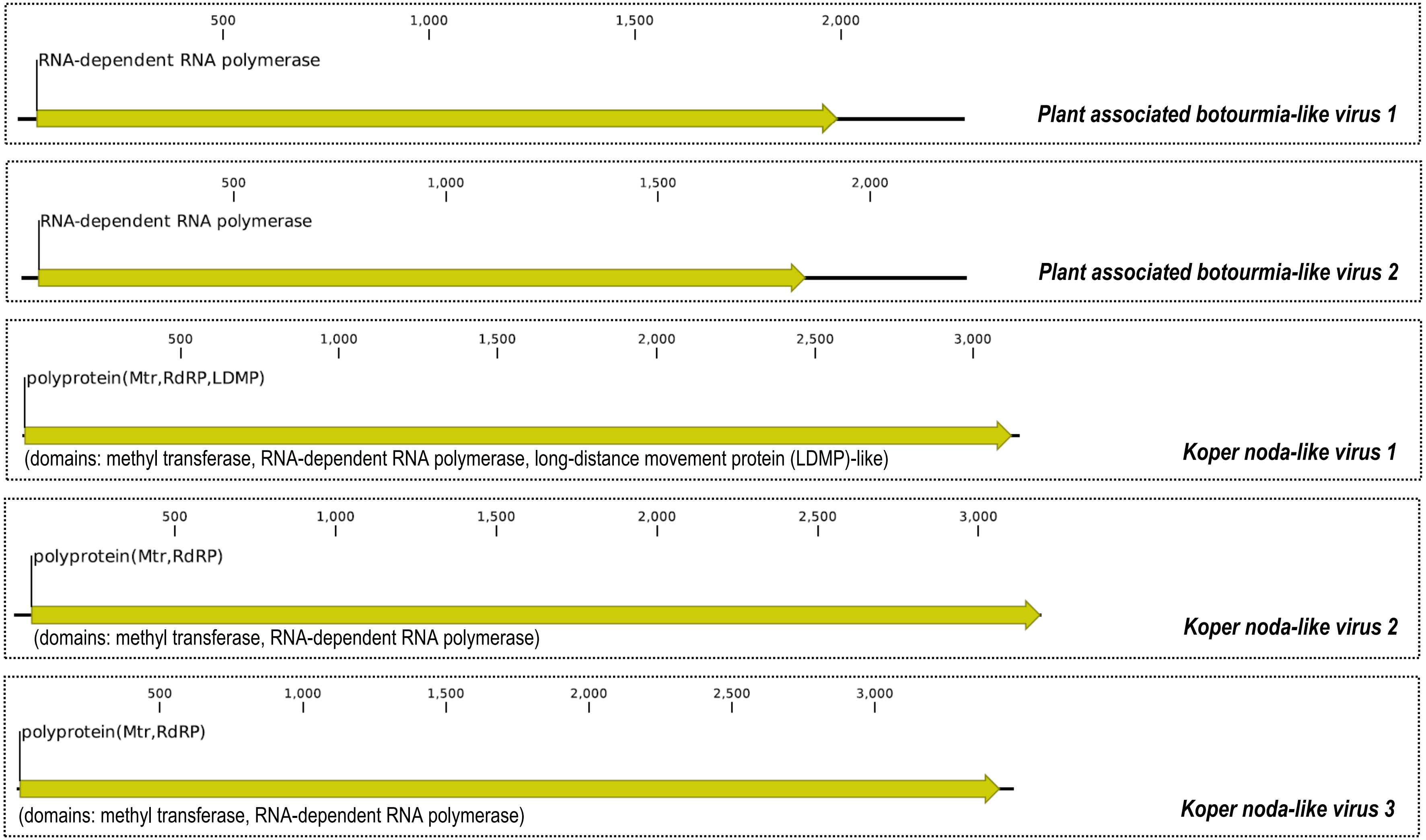

Supplementary Figure 1-12. Genome of new but unclassified virus species discovered under realm *Riboviria.*

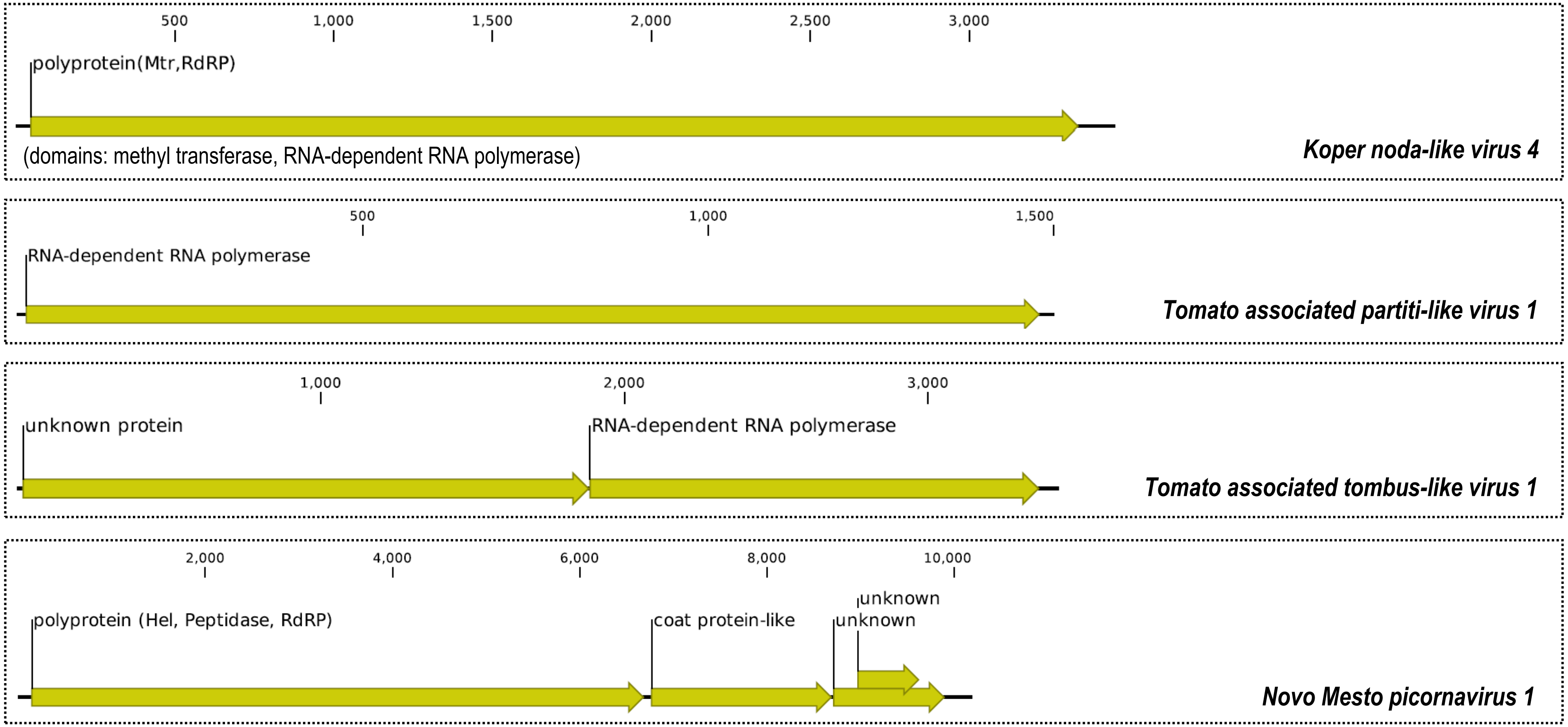

Supplementary Figure 1-12 (continued)**.** Genome of new but unclassified virus species discovered under Realm *Riboviria.*

Supplementary Figure 1 (continued)**.** Genome organization of novel viruses or first full genomes of known viruses, showing known and putative open reading frames and the protein it codes for, and predicted secondary structures of selected viroid-like circular RNAs detected in this study. **Note**: Genome length in number of bases are shown with a scale. For full information on genome length, protein domains, *etc.,* please refer to Supplementary Table 5, and the corresponding accession in GenBank.

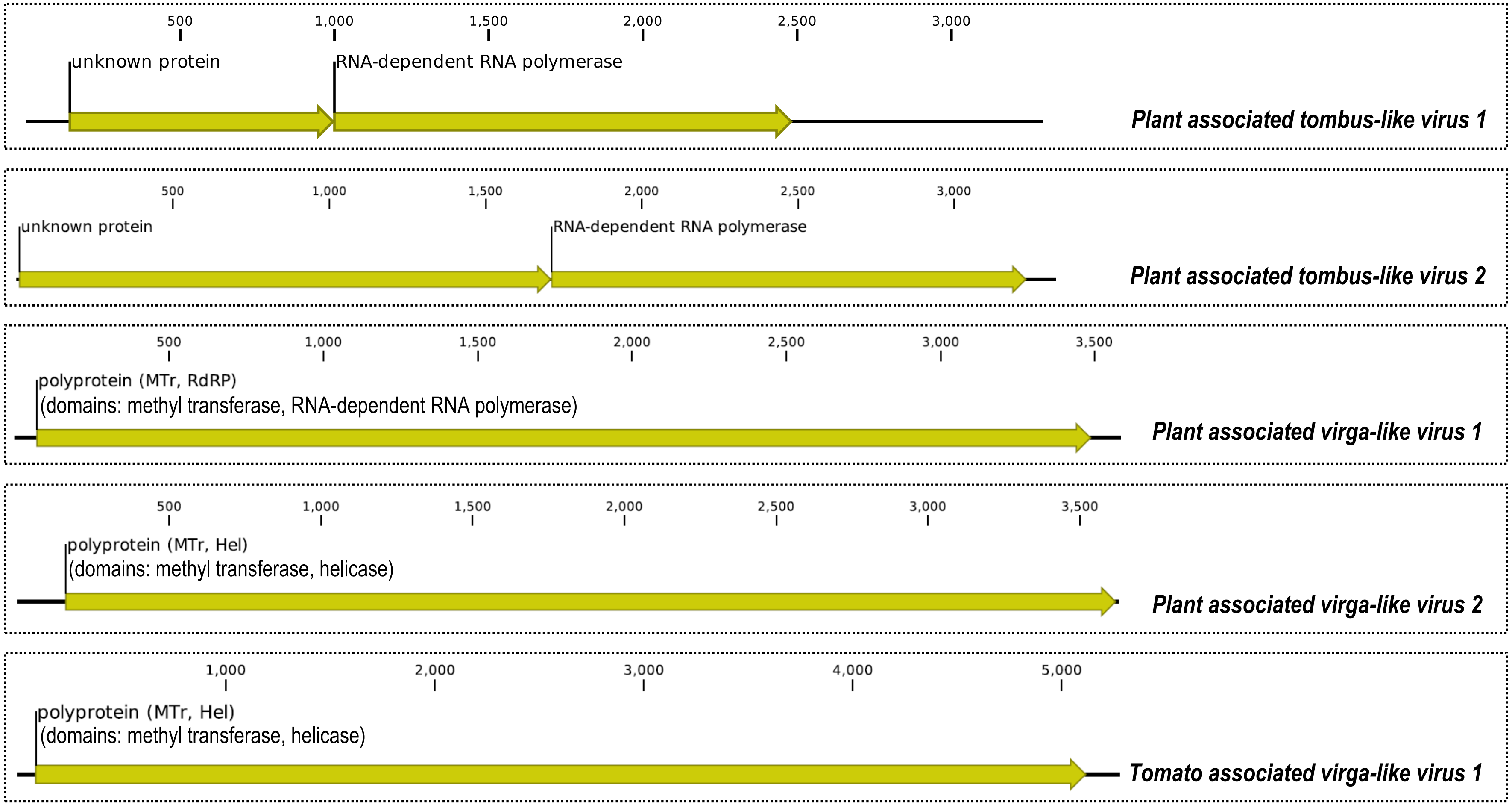

Supplementary Figure 1-12 (continued)**.** Genome of new but unclassified virus species discovered under Realm *Riboviria.*

Supplementary Figure 1 (continued)**.** Genome organization of novel viruses or first full genomes of known viruses, showing known and putative open reading frames and the protein it codes for, and predicted secondary structures of selected viroid-like circular RNAs detected in this study. **Note**: Genome length in number of bases are shown with a scale. For full information on genome length, protein domains, *etc.,* please refer to Supplementary Table 5, and the corresponding accession in GenBank.

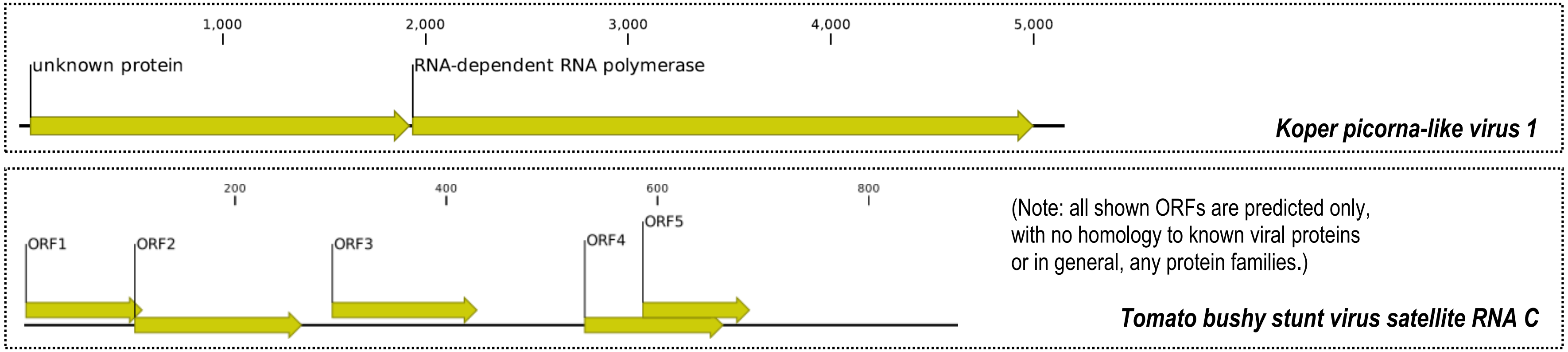

Supplementary Figure 1-12 (continued)**.** Genome of new but unclassified virus species discovered under Realm *Riboviria.*

Supplementary Figure 1 (continued)**.** Genome organization of novel viruses or first full genomes of known viruses, showing known and putative open reading frames and the protein it codes for, and predicted secondary structures of selected viroid-like circular RNAs detected in this study. **Note**: Genome length in number of bases are shown with a scale. For full information on genome length, protein domains, *etc.,* please refer to Supplementary Table 5, and the corresponding accession in GenBank.

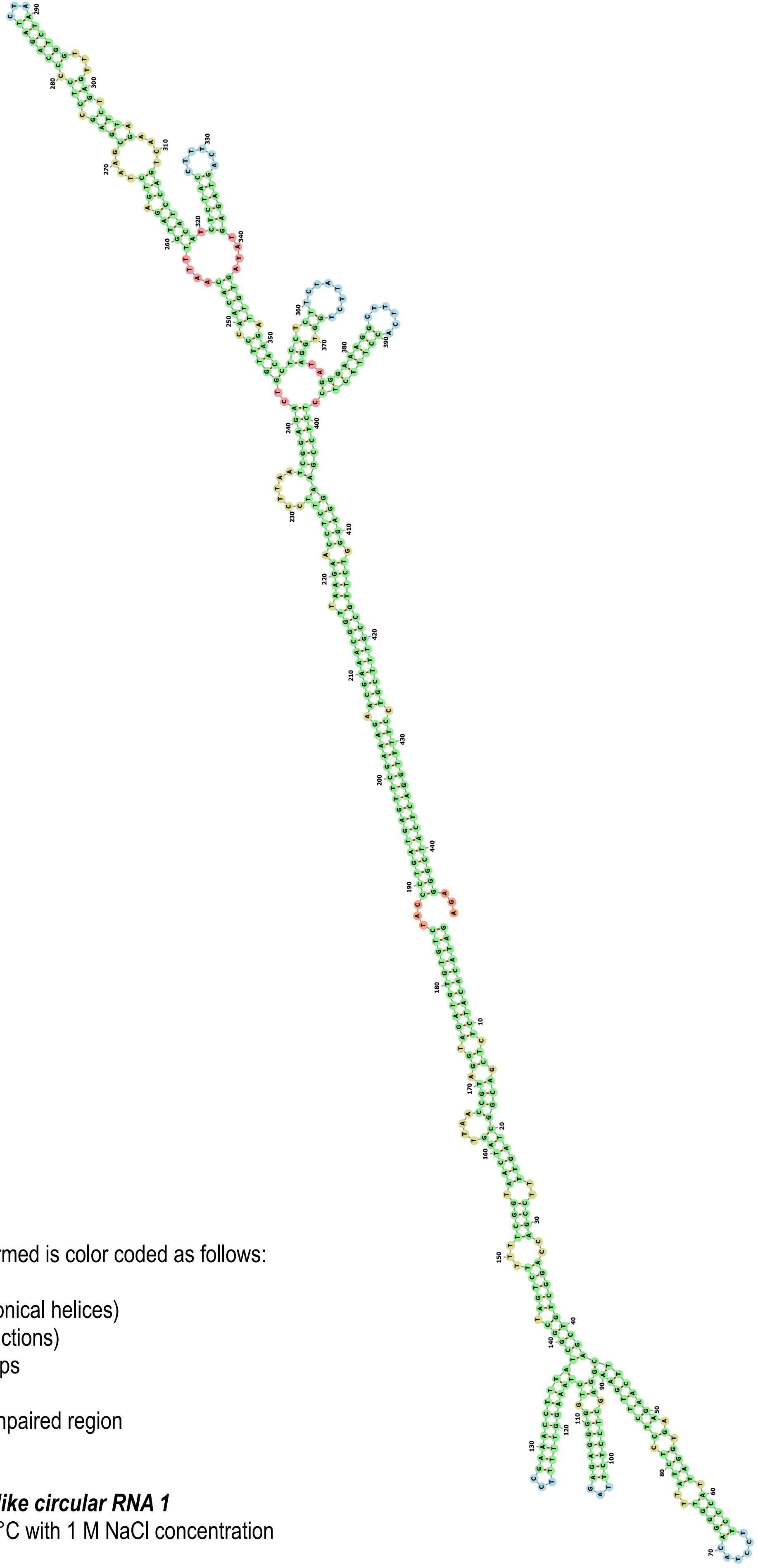

Type of structure formed is color coded as follows:

Green: Stems (canonical helices)
Red: Multiloops (junctions)
Yellow: Interior Loops
Blue: Hairpin loops
Orange: 5' and 3' unpaired region

Taraxacum viroid-like circular RNA 1
dG = -194.35, at 37°C with 1 M NaCl concentration

Supplementary Figure 1-13. Predicted secondary structures of selected viroid-like circular RNAs detected in this study.

Supplementary Figure 1 (continued)**.** Genome organization of novel viruses, or first full genomes of known viruses showing known and putative open reading frames and the protein it codes for, and predicted secondary stuctures of viroid-like circular RNAs detected in this study. **Note**: Genome length in number of bases are shown with a scale. For full information on genome length, protein domains, etc., please refer to Supplementary Table 5 (of the Supplementary Information), and the corresponding accession in GenBank.

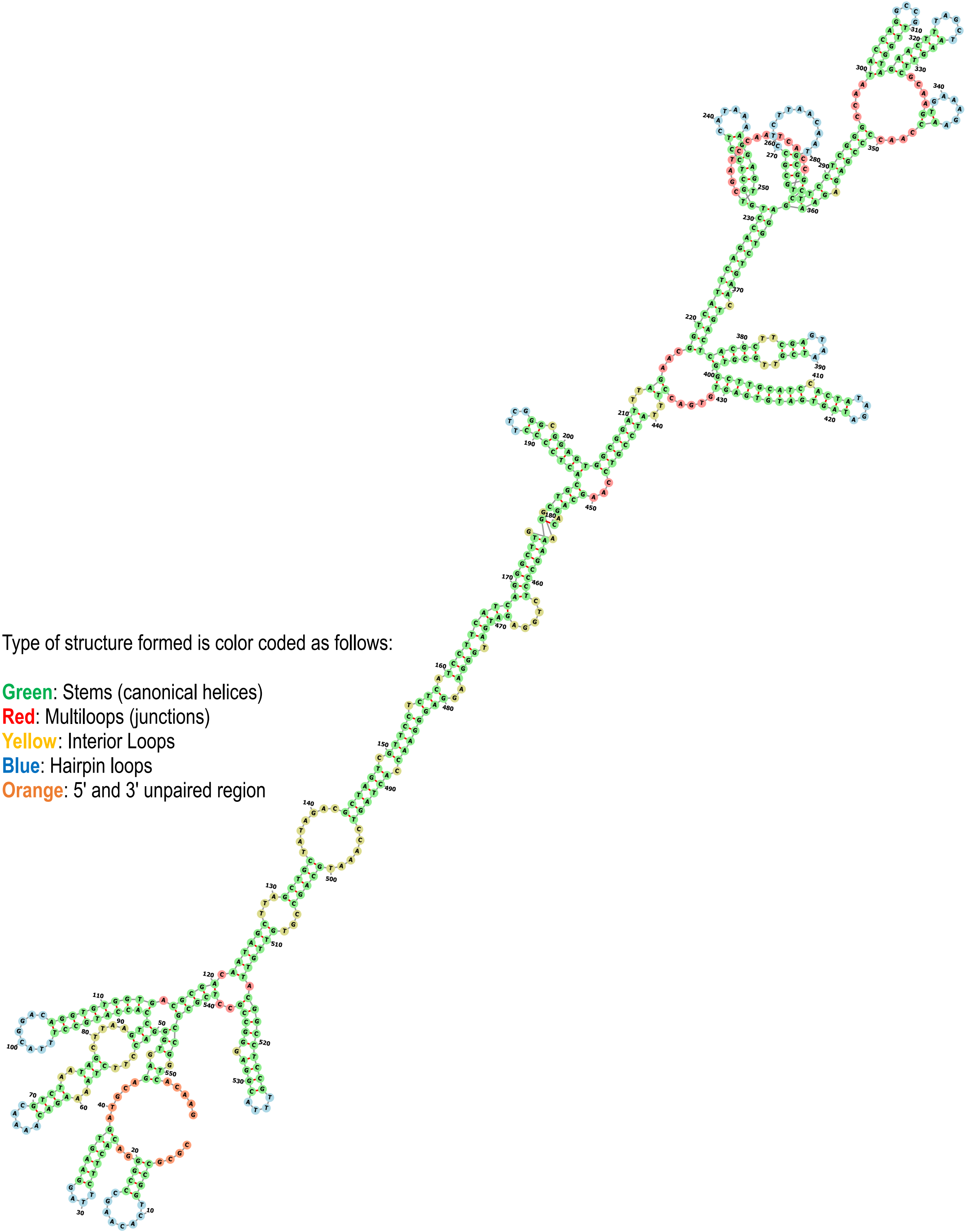

Type of structure formed is color coded as follows:

Green: Stems (canonical helices)
Red: Multiloops (junctions)
Yellow: Interior Loops
Blue: Hairpin loops
Orange: 5' and 3' unpaired region

Plant associated viroid-like circular RNA 1 dG = -181.90, at 37°C with 1 M NaCl concentration

Supplementary Figure 1-13 (continued). Predicted secondary structures of selected viroid-like circular RNAs detected in this study.

Supplementary Figure 1 (continued)**.** Genome organization of novel viruses, or first full genomes of known viruses showing known and putative open reading frames and the protein it codes for, and predicted secondary stuctures of viroid-like circular RNAs detected in this study. **Note**: Genome length in number of bases are shown with a scale. For full information on genome length, protein domains, etc., please refer to Supplementary Table 5 (of the Supplementary Information), and the corresponding accession in GenBank.

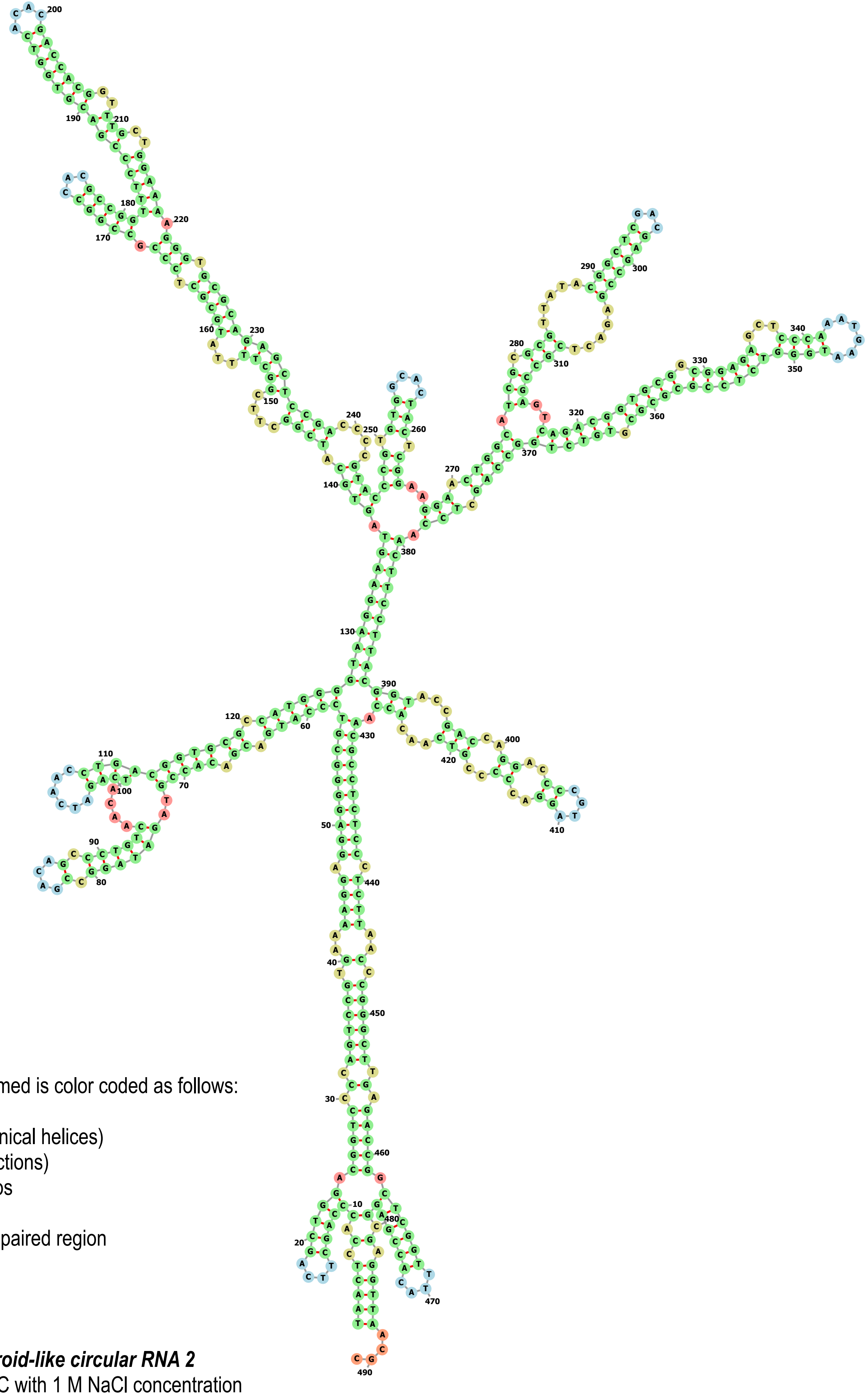

Type of structure formed is color coded as follows:

Green: Stems (canonical helices)
Red: Multiloops (junctions)
Yellow: Interior Loops
Blue: Hairpin loops
Orange: 5' and 3' unpaired region

Plant associated viroid-like circular RNA 2
dG = -247.60, at 37°C with 1 M NaCl concentration

Supplementary Figure 1-13 (continued). Predicted secondary structures of selected viroid-like circular RNAs detected in this study.

Supplementary Figure 1 (continued)**.** Genome organization of novel viruses, or first full genomes of known viruses showing known and putative open reading frames and the protein it codes for, and predicted secondary stuctures of viroid-like circular RNAs detected in this study. **Note**: Genome length in number of bases are shown with a scale. For full information on genome length, protein domains, etc., please refer to Supplementary Table 5 (of the Supplementary Information), and the corresponding accession in GenBank.

Type of structure formed is color coded as follows:

Green: Stems (canonical helices)
Red: Multiloops (junctions)
Yellow: Interior Loops
Blue: Hairpin loops
Orange: 5' and 3' unpaired region

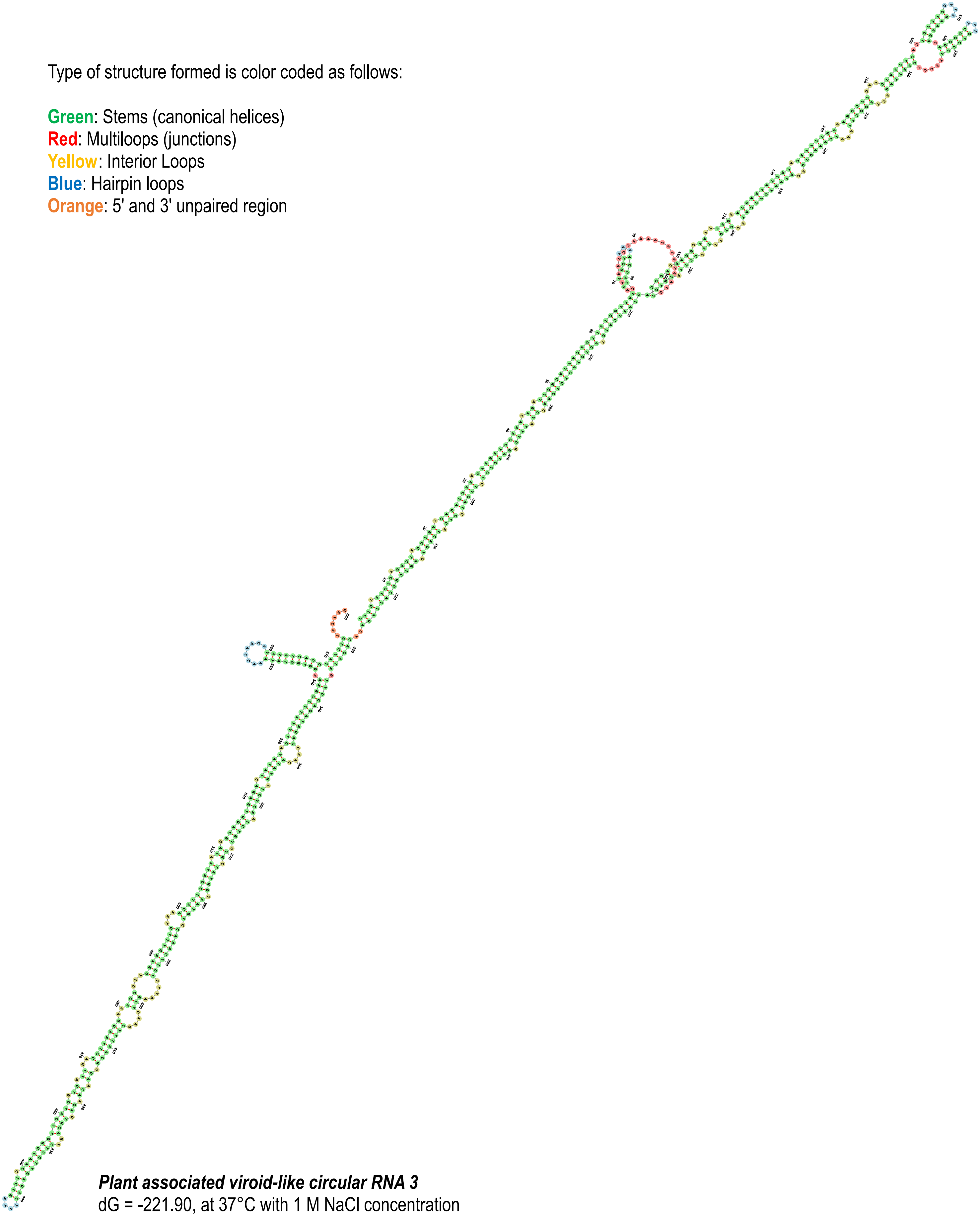

Plant associated viroid-like circular RNA 3
dG = -221.90, at 37°C with 1 M NaCl concentration

Supplementary Figure 1-13 (continued). Predicted secondary structures of selected viroid-like circular RNAs detected in this study.

Supplementary Figure 1 (continued**)**. Genome organization of novel viruses, or first full genomes of known viruses showing known and putative open reading frames and the protein it codes for, and predicted secondary stuctures of viroid-like circular RNAs detected in this study. **Note**: Genome length in number of bases are shown with a scale. For full information on genome length, protein domains, etc., please refer to Supplementary Table 5 (of the Supplementary Information), and the corresponding accession in GenBank.

Type of structure formed is color coded as follows:

Green: Stems (canonical helices)
Red: Multiloops (junctions)
Yellow: Interior Loops
Blue: Hairpin loops
Orange: 5' and 3' unpaired region

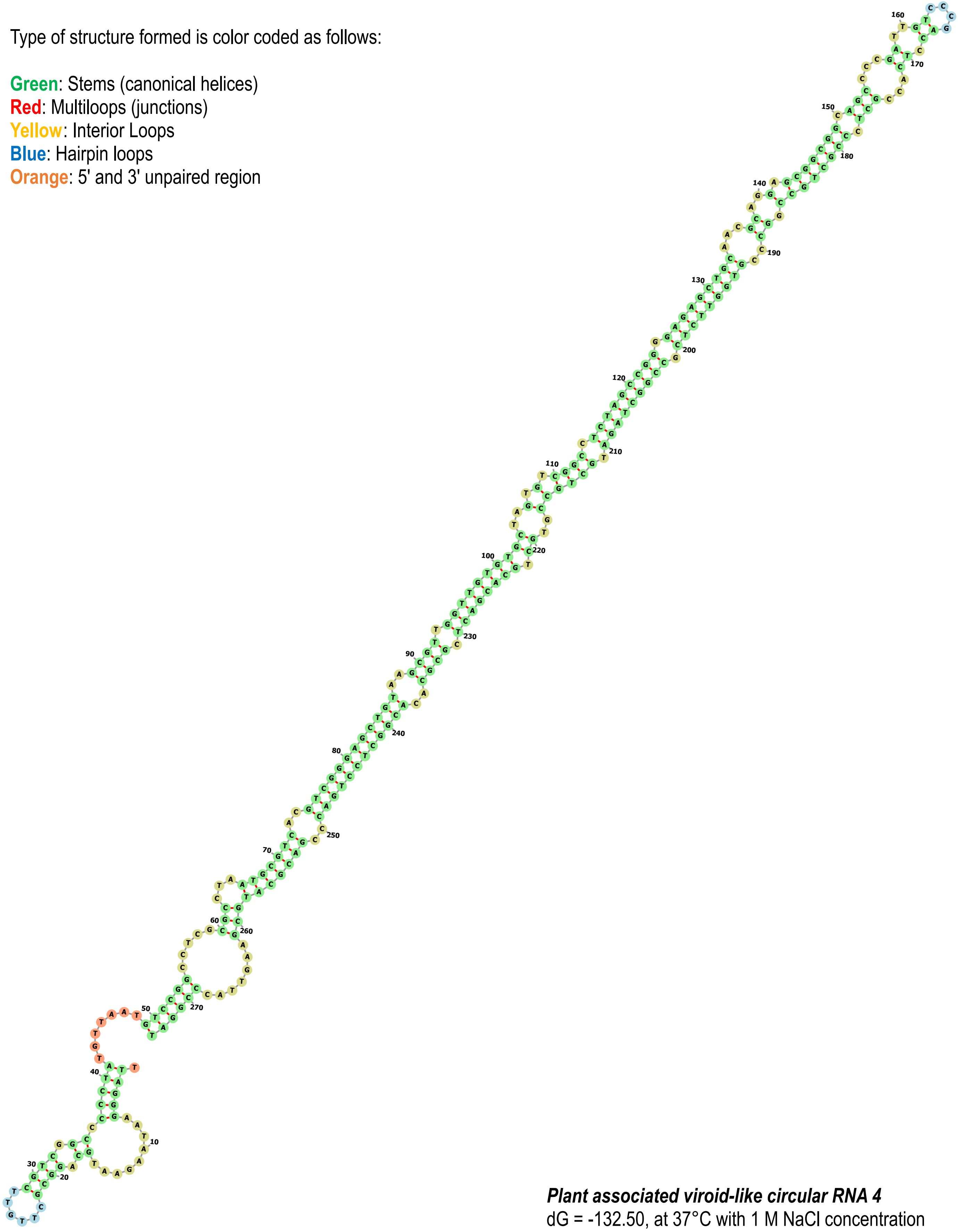

Plant associated viroid-like circular RNA 4
dG = -132.50, at 37°C with 1 M NaCl concentration

Supplementary Figure 1-13 (continued). Predicted secondary structures of selected viroid-like circular RNAs detected in this study.

Supplementary Figure 1 (continued)**.** Genome organization of novel viruses, or first full genomes of known viruses showing known and putative open reading frames and the protein it codes for, and predicted secondary stuctures of viroid-like circular RNAs detected in this study. **Note**: Genome length in number of bases are shown with a scale. For full information on genome length, protein domains, etc., please refer to Supplementary Table 5 (of the Supplementary Information), and the corresponding accession in GenBank.
