# Supplementary material for: In-depth study of tomato and weed viromes reveals undiscovered plant virus diversity in an agroecosystem
Source: Microbiome. 2023 Mar 28;11:60. doi: 10.1186/s40168-023-01500-6 (PMC10042675; doi:10.1186/s40168-023-01500-6)
Supplement: Supplementary file 5 — Additional file 4: Supplementary Table 7. RT-PCR primers and PCR conditions used in confirmation of associated plant hosts of selected viruses and putative viroids. Supplementary Table 8. RT-PCR thermocycling conditions used in the detection of selected viruses and putative viroids in associated plant hosts. Supplementary Figure 2. Orientation and annealing sites of the primers designed for the amplification of the circular genome of Taraxacum viroid-like circular RNA 1, and results of RT-PCR confirmation of the circular genome. Supplementary Table 9. List of confirmed associated plant hosts of selected viruses and putative viroids. [file 40168_2023_1500_MOESM4_ESM.pdf]

In-depth study of tomato and weed viromes reveals undiscovered plant virus diversity in an agroecosystem

Rivarez, MPS[1,2,*], Pecman, A[1,2], Bačnik, K[1,2], Maksimović Carvalho Ferreira, O[1,2], Vučurović, A[1], Seljak, G[1], Mehle, N[1,3], Gutiérrez-Aguirre, I[1], Ravnikar, M[1], and Kutnjak, D[1,*]

[1]Department of Biotechnology and Systems Biology, National Institute of Biology, Večna pot 111, Ljubljana, 1000 Slovenia. [2]Jožef Stefan International Postgraduate School, Jamova cesta 39, Ljubljana, 1000 Slovenia. [3]School for Viticulture and Enology, University of Nova Gorica, Dvorec Lanthieri Glavni trg 8, Vipava, 5271 Slovenia. *for correspondence email Denis Kutnjak (denis.kutnjak@nib.si) and Mark Paul Selda Rivarez (mpsrivarez@gmail.com)

SUPPLEMENTARY INFORMATION

Additional File 04

Supplementary Table 7. RT-PCR primers and PCR conditions used in confirmation of associated plant hosts of selected viruses and putative viroids. **Supplementary Table 8.** RT-PCR thermocycling conditions used in the detection of selected viruses and putative viroids in associated plant hosts. **Supplementary Figure 2.** Orientation and annealing sites of the primers designed for the amplification of the circular genome of Taraxacum viroid-like circular RNA 1, and results of RT-PCR confirmation of the circular genome. **Supplementary Table 9**. List of confirmed associated plant hosts of selected viruses and putative viroids.

Supplementary Table 7. RT-PCR primers and PCR conditions used in confirmation of associated plant hosts of selected viruses and a putative viroid. Note: GenBank accession numbers of the species indicated above are in Supplementary Table 5.

No.	Target virus name	Primer ID	Strand	Sequence (5'→3')	Length (bases)	Target genome region (start, end position / gene(s))			Annealing tempera-ture. (°C)	Amplicon size (bases)
						Start	End	Gene		
01	Artemesia fimovirus 1	NFimo1-F<br>NFimo1-R	forward<br>reverse	ACATGGAAGTGACAGGTTCTCC<br>ATAGTTGACGCCTACCCATTGC	22<br>22	5889	6435	RdRP	57	547
02	Calystegia geminivirus 1	NGemi1-F<br>NGemi1-R	forward<br>reverse	CAGAACCTCAACCTTCCATTCC<br>CCAATCAGCTCTTTCCAGTGC	22<br>21	248	817	replicase	57	570
03	Plantago potyvirus 1	NPoty1-F<br>NPoty1-R	forward<br>reverse	AGGCTAGAGATCGCAAACTTGG<br>CAACTTTATCCGTGCTCTGTGG	22<br>22	5687	6550	polyprotein	57	864
04	Mentha macluravirus 1	NPoty2-F<br>NPoty2-R	forward<br>reverse	ACATACGGCTCAGCTTTCTTCC<br>GGCACAGAGAACTTCAACATCG	22<br>22	2474	3306	polyprotein	57	833
05	Rumex potyvirus 1	NPoty3-F<br>NPoty3-R	forward<br>reverse	GTTGACCTAACCCCTCACAACC<br>CACTACTGGTAGCCCACACTGC	22<br>22	7098	7733	polyprotein	57	636
06	broad-leafed dock virus A, isolate 2	NPoty4-F<br>NPoty4-R	forward<br>reverse	GGATGAGGAATATGGAGCTTGG<br>TACTAGGCGGTGGAAGAAAACC	22<br>22	7502	8178	polyprotein	57	677
07	Pastinaca umbravirus 1	NTomb1-F<br>NTomb1-R	forward<br>reverse	GCGAGACTGTCTGTACCACTGC<br>CAGTCGTACCCCCTCTAACTGG	22<br>22	2504	3354	RdRP-MP	57	851
08	Picris umbravirus 1	NTomb2-F<br>NTomb2-R	forward<br>reverse	TCGTGCTGTATAGGGTTCATGG<br>CAAACTCCCCAAATGGACTACC	22<br>22	1846	2839	RdRP	57	994
09	Convolvulus aureusvirus 1	NTomb3-F<br>NTomb3-R	forward<br>reverse	GAAAGTCAGCCGAATTGTAGGG<br>CAATCCAAGTTGGTCCTTCTCC	22<br>22	1088	1814	RdRP	57	727
10	Calystegia pelarspovirus 1	NTomb4-F<br>NTomb4-R	forward<br>reverse	GCCGAATGAGGGTATGTTTAGG<br>GTTCCGCTGTTCTTCAACTGG	22<br>21	1019	1594	RdRP	57	576
11	Cichorium alphacarmovirus 1	NTomb6-F<br>NTomb6-R	forward<br>reverse	GGAGAAACATGAGGAACGAACC<br>AGAAAACCCTTTCCAGGAGACC	22<br>22	322	989	RdRP	57	668
12	Pastinaca potexvirus 1	NAflex1-F<br>NAflex1-R	forward<br>reverse	CACAGTGGATGAGGATGTAGCC<br>TTCGTAAGCTGAGCTGAGTTGG	22<br>22	3565	4542	polypro-tein-TGB	57	978
13	plant associated tobamo-like virus 1	NVirga1-F<br>NVirga1-R	forward<br>reverse	CTTCACCTGTCTCAGTGAGGAC<br>TATGAGTTGCGATGGGTAGACG	22<br>22	115	522	replicase	55	408
14	Plantago tobamovirus 1	NVirga2-F<br>NVirga2-R	forward<br>reverse	AACGCACTATCCGAGCTATCTG<br>TACAGCCACCCTAAACCATGTC	22<br>22	1720	2421	replicase	55	702

15	Mercurialis orthotospovirus 1	NTospo1-F<br>NTospo1-R	forward<br>reverse	TAGAGCCGAAGATGTTGTGGAC<br>GTCAGCGACCATTAAGCCTTTG	22<br>22	1661	2291	L gene (RdRP)	55	631
16	tomato associated bunya-like virus 1	NTospo2-F<br>NTospo2-R	forward<br>reverse	CGAAAGGAGGCGATAGTGATGC<br>CTGCGTCATCCCTACCTGATAC	22<br>22	7109	7615	L gene (RdRP)	56	507
17	tomato vitivirus 1	NBflexi1-F<br>NBflexi1-R	forward<br>reverse	TCTTTCCCCTCTTGATCTGTGC<br>GTGAACCCTGAATTGGTTGAGC	22<br>22	1057	1540	movement protein	55	484
18	Prunus virus I	NBromo1-F<br>NBromo1-R	forward<br>reverse	AAGTTTCGAGACCTTTGCGTTG<br>CTCAAACACACTTCCGCTTCAG	22<br>22	826	1699	movement protein	55	874
19	tomato ilarvirus 1	NBromo2-F<br>NBromo2-R	forward<br>reverse	ACATGGCGTTAGATGGTAGGTC<br>AAATTCGCAGACAAGGTTCGTG	22<br>22	945	1824	movement protein	55	880
20	Ranunculus white mottle ophiovirus	RWMV-F<br>RWMV-R	forward<br>reverse	TGTGTGTTCATCTCTCTTCTGC<br>ACAGGGAAGTGAATCACACCTA	22<br>22	804	1296	coat protein	54	493
21	tomato betanucleorhabdo-virus 1	NRhabdo1-F<br>NRhabdo1-R	forward<br>reverse	GACGGTAGGTTACAATCTCC<br>TGATAGGGCTAGGATATGGG	20<br>20	12078	12589	L gene (RdRP)	55	512
22	Pastinaca cytorhabdovirus 1	NRhab3-F<br>NRhab3-R	forward<br>reverse	GAGGAAAAAGTCTGTCATGGAC<br>GCAAGGTAATAATAGCACTCGG	22<br>22	1395	1925	L gene (RdRP)	52	531
23	tomato betanucleorhabdo-virus 2	NRhab4-F<br>NRhab4-R	forward<br>reverse	TTCCTGTTCATTATCACAATGC<br>GTTAGTTGACCAAGAGTACCAG	22<br>22	4279	4874	L gene (RdRP)	51	596
24	Picris betanucleorhabdo-virus 1	NRhab5-F<br>NRhab5-R	forward<br>reverse	ATTGTTACACGATATTGCTGGG<br>TTCCTCATATCTCCACCTCAAC	22<br>22	3300	3816	L gene (RdRP)	52	517
25	Cirsium cytorhabdovirus 1	NRhab6-F<br>NRhab6-R	forward<br>reverse	TTTAGTTAGATCATTCACGGCG<br>TGAGGTCCCTTGATAATCGATC	22<br>22	2204	2742	L gene (RdRP)	52	539
26	Taraxacum betanucleorhabdovirus 1	NRhab7-F<br>NRhab7-R	forward<br>reverse	ATAGTTCGGACAGATCAAGGAG<br>ATCTCAAATGTTTGCCACTCTC	22<br>22	5196	5757	L gene (RdRP)	52	562
27	Picris cytorhabdovirus 1	NRhab8-F<br>NRhab8-R	forward<br>reverse	TCGACCAAAAGATAACAACGAC<br>CTTTGAAAATCACTAGTCCGGG	22<br>22	5798	6389	L gene (RdRP)	52	592
28	Taraxacum cytorhabdovirus 1	NRhab9-F<br>NRhab9-R	forward<br>reverse	CTGTATGTGGTGAAGTCAATGG<br>TCTCATTCTTTTCGCTTCTTCG	22<br>22	2799	3347	L gene (RdRP)	52	549
29	tomato alphanucleorhabdo-virus 1	NRhab10-F<br>NRhab10-R	forward<br>reverse	GATTGTATTTCCCACTACGGACAAC<br>CATACCATCATCACATAGTGTTGGC	25<br>25	3182	4230	L gene (RdRP)	55	1049
30	Leveillula taurica associated rhabdo-like virus 1	NRhab2-F<br>NRhab2-R	forward<br>reverse	CCACATTATGACACAAGACCAG<br>TAAGCTTTGTTACCTAACGCAC	22<br>22	330	844	L gene (RdRP)	52	515
31	eggplant mottled dwarf alphanucleorhabdovirus	EMDV-F<br>EMDV-R	forward<br>reverse	TACTCATTACACAAAGAGAAGC<br>CGGTATAGTTATACTAGCAGCA	22<br>22	143	699	L gene (RdRP)	51	557

32	Physostegia chlorotic mottle alphanucleorhabdovirus	PhCMoV-F<br>PhCMoV-R	forward<br>reverse	ATAGTGACATTCTGTTTGACCG<br>CCCATACTACCCATTATTCTGC	22<br>22	2810	3463	L gene (RdRP)	52	654
33	tomato fruit blotch virus	ToFBV-R3-F<br>ToFBV-R3-R	forward<br>reverse	GTGGTTATTATGGATATACCTGCG<br>GAGAGAACACAAAACAAGAAGC	24<br>22	654	1371	coat protein	52	718
34	Solanum nigrum ilarvirus 1	SnIV-R3-F<br>SnIV-R3-R	forward<br>reverse	GTATGAAAACTTCAACCTCTCC<br>ATATAGCTACCCAGAAATCAGC	22<br>22	1248	1916	coat protein	52	669
35	tomato matilda virus	TMaV-F<br>TMaV-R	forward<br>reverse	ACTAGCCGTTATATTTAGTGGG<br>CTACTATACTGAGAACTCCTTTCC	22<br>24	5293	5900	polyprotein	52	608
36	Taraxacum viroid-like circular RNA 1	NVrd1-F-LP<br>NVrd1-R-LP	forward<br>reverse	TCGGCTAGTCTTTTTCGGTAAC<br>AGGTGAAAGCCTTTCCCTATCC	22<br>22	138	392	not applicable	55	255
	(Note: LP - linear amplification, C - circular amplification)	NVrd1-F-C<br>NVrd1-R-C	primer 1<br>primer 2	GGCTCGCTTAGACTCTACAAATTG<br>CTGGTTTGAGTCTTAGAACTGACC	24<br>24	277<br>(+) strand	291<br>(-) strand	not applicable	55	433

Supplementary Table 8. RT-PCR thermocycling conditions used in the detection of selected viruses and putative viroids in associated plant hosts.

Reverse transcription step (2 sub-steps):	30 min at 50ºC<br>15 min at 95ºC	
PCR step (3 sub-steps):	0.5 min at 94ºC<br>0.5 min at annealing temperature (ºC) in **Supplementary Table 7**<br>1.0 min at 72ºC	35 cycles
Extension step:	10 min at 72ºC	

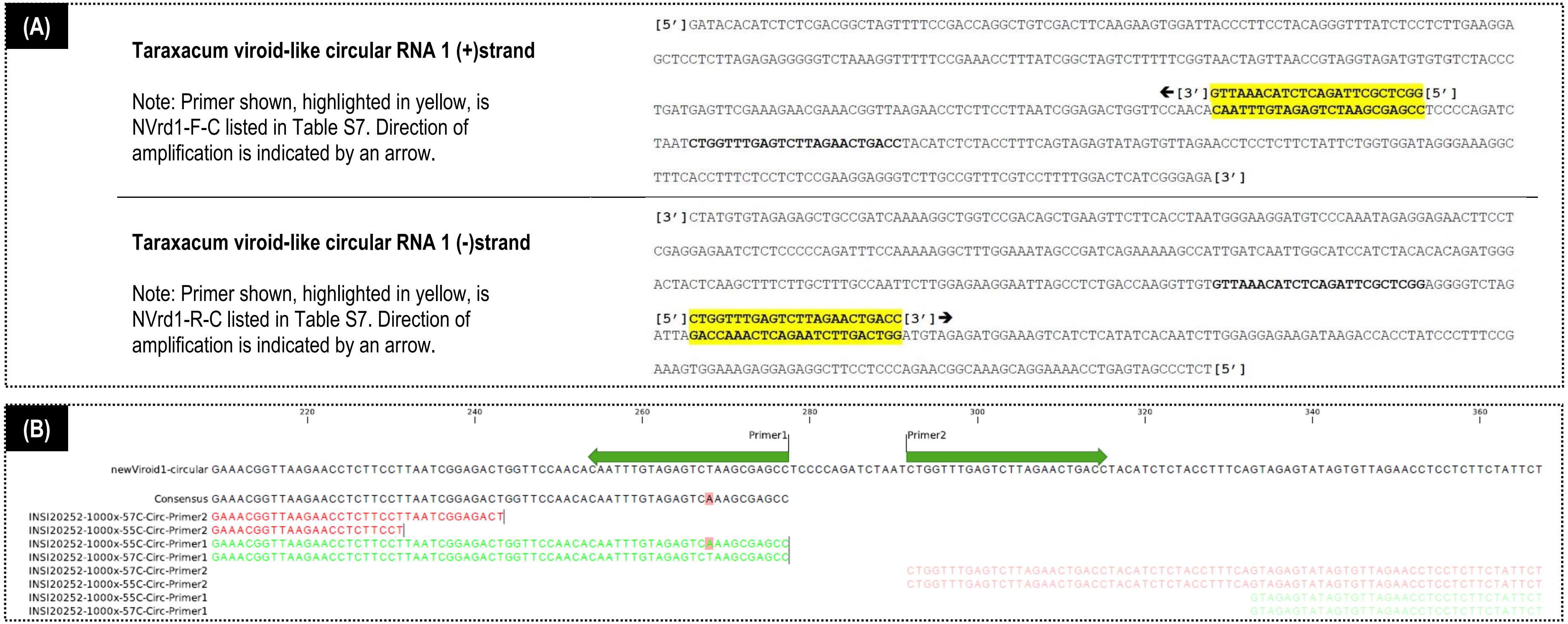

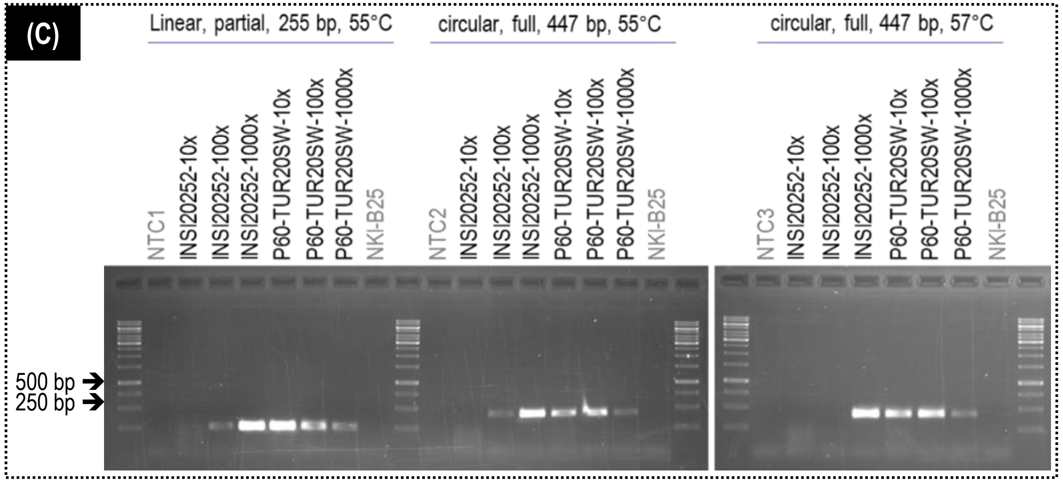

Supplementary Figure 2. Primer design and RT-PCR tests for Taraxacum viroid-like circular RNA 1. (A) Orientation and annealing sites of the primers designed to amplify the circular genome of Taraxacum viroid-like circular RNA 1. (B-C) Reassembly of selected sequenced amplicons form the RT-PCR tests (B) in samples with increasing dilution factors shown in (C). Abutting primers are shown in green arrows, demarcating the 14 nucleotide gap between them (B). Note: In (C): NTC1, NTC2 – non-template controls; NKI-B25 - negative control of isolation in the RNA extraction, INSI20252 - *Taraxacum officinale* sample, P60-TUR20SW - sequenced composite sample where INSI20252 belong.

Supplementary Table 9. List of confirmed associated plant hosts of selected viruses. For information related to the individual plant samples and the composite samples, please refer to Supplementary Table 1 and 2. Note: In most of the cases infection of multiple viruses in individual plants with cannot be excluded, except if stated otherwise.

No.	Virus name	Sample ID	Tissue	Plant species name	Plant family	Symptoms (if any)
01	Artemesia fimovirus 1	INSI20094	leaf	*Artemisia verlotiorum*	Asteraceae	mild leaf yellowing
02	Calystegia geminivirus 1	INSI20122	leaf	*Calystegia* sp.	Convolvulaceae	leaf yellowing with crumpling deformation
03	Plantago potyvirus 1	INSI19064	leaf	*Plantago lanceolata*	Plantaginaceae	interveinal leaf yellowing with deformation
04	Mentha macluravirus 1	INSI20169	leaf	*Mentha spicata*	Lamiaceae	mild leaf yellowing with a few necrotic spots
05	Rumex potyvirus 1	INSI20188	leaf	*Rumex* sp.	Polygonaceae	ring-like chlorotic lesions and red spots on leaves
		INSI20199	leaf	*Convolvulus* sp.	Convolvulaceae	leaf chlorosis
06	Broad-leafed dock virus A, isolate 2	INSI20253	leaf	*Rumex crispus*	Polygonaceae	leaf chlorosis with mottling
07	Pastinaca umbravirus 1	INSI19137	leaf	*Pastinaca sativa*	Apiaceae	necrotic spots, mosaic on leaves
		INSI19156	leaf	*Pastinaca sativa*	Apiaceae	irregular leaf chlorosis
		INSI20168	leaf	*Pastinaca sativa*	Apiaceae	irregular leaf chlorosis
08	Picris umbravirus 1	INSI19136	leaf	*Picris echoides*	Asteraceae	necrotic leaf lesions
		INSI20082	leaf	*Picris echoides*	Asteraceae	chlorotic leaf spots
09	Convolvulus aureusvirus 1	INSI20072	leaf	*Convolvulus arvensis*	Convolvulaceae	necrotic leaf lesions
		INSI20073	leaf	*Convolvulus arvensis*	Convolvulaceae	leaf curling with mild yellowing
10	Calystegia pelarspovirus 1	INSI20122	leaf	*Calystegia* sp.	Convolvulaceae	leaf yellowing with crumpling deformation
		INSI20163	leaf	*Calystegia* sp.	Convolvulaceae	necrotic leaf lesions with yellow halo
11	Cichorium alphacarmovirus 1	INSI19123	leaf	*Cichorium intybus*	Asteraceae	leaf mosaic, leaf dwarfing and deformation
		INSI20074	leaf	*Cichorium intybus*	Asteraceae	necrotic leaf lesions
		INSI20075	leaf	*Picris echoides*	Asteraceae	leaf mosaic
		INSI20078	leaf	*Picris echoides*	Asteraceae	redness of leaves, necrosis on flowers
		INSI20082	leaf	*Picris echoides*	Asteraceae	chlorotic leaf spots
12	Pastinaca potexvirus 1	INSI19137	leaf	*Pastinaca sativa*	Apiaceae	necrotic spots, mosaic on leaves
13	Plant associated tobamo-like virus 1	INSI19082	leaf	*Convolvulus arvensis*	Convolvulaceae	with powdery mildew colonization / infection
		INSI20147	leaf	*Solanum lycopersicum*	Solanaceae	none (asymptomatic)
		INSI20152	leaf	*Solanum lycopersicum*	Solanaceae	none (asymptomatic)
		INSI20157	leaf	*Solanum lycopersicum*	Solanaceae	none (asymptomatic)
		INSI20158	leaf	*Solanum lycopersicum*	Solanaceae	none (asymptomatic)

		INSI20159	leaf	*Solanum lycopersicum*	Solanaceae	none (asymptomatic)
		INSI20146	leaf	*Solanum lycopersicum*	Solanaceae	interveinal leaf yellowing
		INSI20148	leaf	*Solanum lycopersicum*	Solanaceae	interveinal leaf yellowing
		INSI20149	leaf	*Solanum lycopersicum*	Solanaceae	interveinal leaf yellowing
		INSI20150	leaf	*Solanum lycopersicum*	Solanaceae	interveinal leaf yellowing
		INSI20151	leaf	*Solanum lycopersicum*	Solanaceae	interveinal leaf yellowing
		INSI20154	fruit	*Solanum lycopersicum*	Solanaceae	irregular yellow discolorations on fruits
		INSI20155	fruit	*Solanum lycopersicum*	Solanaceae	irregular yellow discolorations on fruits
		INSI20156	fruit	*Solanum lycopersicum*	Solanaceae	irregular yellow discolorations on fruits
		INSI20156F	fruit	*Solanum lycopersicum*	Solanaceae	irregular yellow discolorations on fruits
14	Plantago tobamovirus 1	INSI19124	leaf	*Plantago major*	Plantaginaceae	leaf mosaic
15	Mercurialis orthotospovirus 1	INSI19080	leaf	*Mercurialis annua*	Euphorbiaceae	leaf chlorosis with slight deformation
		INSI19098	leaf	*Mercurialis annua*	Euphorbiaceae	leaf chlorosis with deformation and necrotic lesions
		INSI19121	leaf	*Mercurialis annua*	Euphorbiaceae	none (asymptomatic)
		INSI20111	leaf	*Mercurialis annua*	Euphorbiaceae	leaf deformation
		INSI20112	leaf	*Mercurialis annua*	Euphorbiaceae	necrotic leaf spots, shot-hole symtpoms
16	Tomato associated bunya-like virus 1	INSI20083	leaf	*Solanum lycopersicum*	Solanaceae	leaf yellowing with necrotic lesions
		INSI20084	leaf	*Solanum lycopersicum*	Solanaceae	leaf yellowing
		INSI20085	leaf	*Solanum lycopersicum*	Solanaceae	leaf yellowing with necrotic lesions
		INSI20088	leaf	*Solanum lycopersicum*	Solanaceae	leaf deformation
		INSI20088F	fruit	*Solanum lycopersicum*	Solanaceae	yellow discoloration
		INSI20089	leaf	*Solanum lycopersicum*	Solanaceae	leaf yellowing with necrotic lesions
		INSI20090	leaf	*Solanum lycopersicum*	Solanaceae	leaf yellowing with necrotic lesions
17	Tomato vitivirus 1	INSI20124	leaf	*Solanum lycopersicum*	Solanaceae	necrotic leaf lesions
		INSI20124F	fruit	*Solanum lycopersicum*	Solanaceae	yellow discoloration on fruits, necrosis on sepals
18	Prunus virus I	INSI20078	leaf	*Picris echoides*	Asteraceae	red discolorations on leaf and sepal tips
19	Tomato ilarvirus 1	INSI20087	leaf	*Solanum lycopersicum*	Solanaceae	none (asymptomatic)
20	Ranunculus white mottle ophiovirus	INSI19037	leaf	*Solanum lycopersicum*	Solanaceae	necrotic leaf spots
		INSI19040	leaf	*Solanum lycopersicum*	Solanaceae	necrotic leaf spots, discoloration on fruits
		INSI19042	leaf	*Solanum lycopersicum*	Solanaceae	necrotic leaf spots
		INSI19043	leaf	*Solanum lycopersicum*	Solanaceae	mild leaf chlorosis
		INSI19073	leaf	*Solanum lycopersicum*	Solanaceae	none (asymptomatic)
		INSI19074	leaf	*Solanum lycopersicum*	Solanaceae	leaf yellowing with necrotic lesions

		INSI19102	leaf	*Solanum nigrum*	Solanaceae	none (asymptomatic)
		INSI20177	leaf	*Solanum lycopersicum*	Solanaceae	leaf folding
		INSI20177F	fruit	*Solanum lycopersicum*	Solanaceae	fruit deformation, cracking
21	Tomato betanucleorhabdovirus 1	INSI19008	leaf	*Solanum lycopersicum*	Solanaceae	leaf folding
22	Pastinaca cytorhabdovirus 1	INSI19137	leaf	*Pastinaca sativa*	Apiaceae	necrotic spots, mosaic on leaves
23	Tomato betanucleorhabdovirus 2	INSI20041	leaf	*Solanum lycopersicum*	Solanaceae	none (asymptomatic)
		INSI20068	leaf	*Solanum lycopersicum*	Solanaceae	none (asymptomatic)
		INSI20123	leaf	*Solanum lycopersicum*	Solanaceae	leaf yellowing and deformation, uneven fruit ripening
		INSI20132	leaf	*Solanum lycopersicum*	Solanaceae	leaf yellowing and deformation
		INSI20133	leaf	*Solanum lycopersicum*	Solanaceae	none (asymptomatic)
24	Picris betanucleorhabdovirus 1	INSI20078	leaf	*Picris echoides*	Asteraceae	red discolorations on leaf and sepal tips
		INSI20080	leaf	*Picris echoides*	Asteraceae	red discolorations on leaf and sepal tips
		INSI20138	leaf	*Picris echoides*	Asteraceae	cholorotic and necrotic spots
25	Cirsium cytorhabdovirus 1	INSI20161	leaf	*Cirsium arvense*	Asteraceae	leaf yellowing (near leaf lamina)
26	Taraxacum betanucleorhabdovirus 1	INSI20194	leaf	*Taraxacum officinale*	Asteraceae	systemic leaf chlorosis
		INSI20252	leaf	*Taraxacum officinale*	Asteraceae	interveinal leaf chlorosis
27	Picris cytorhabdovirus 1	INSI20080	leaf	*Picris echoides*	Asteraceae	red discolorations on leaf and sepal tips
		INSI20081	leaf	*Picris echoides*	Asteraceae	leaf mosaic
		INSI20082	leaf	*Picris echoides*	Asteraceae	red lesions on leaves and midribs
28	Taraxacum cytorhabdovirus 1	INSI20194	leaf	*Taraxacum officinale*	Asteraceae	systemic leaf chlorosis
29	Tomato alphanucleorhabdovirus 1	INSI20029	leaf	*Solanum lycopersicum*	Solanaceae	fruit mottling, showing large irregular shape yellow dents
		INSI20030	leaf	*Solanum lycopersicum*	Solanaceae	fruit mottling, showing large irregular shape yellow dents
		INSI20030F	fruit	*Solanum lycopersicum*	Solanaceae	fruit mottling, showing large irregular shape yellow dents
30	Leveillula taurica associated rhabdo-like virus 1	INSI19051	leaf	*Solanum lycopersicum*	Solanaceae	leaves with powdery mildew colonization, necrotic spots
		INSI19053	leaf	*Solanum lycopersicum*	Solanaceae	leaves with powdery mildew colonization, necrotic spots
		INSI19054	leaf	*Solanum lycopersicum*	Solanaceae	leaves with powdery mildew colonization, necrotic spots
		INSI19055	leaf	*Solanum lycopersicum*	Solanaceae	leaves with powdery mildew colonization, necrotic spots
		INSI19056	leaf	*Solanum lycopersicum*	Solanaceae	leaves with powdery mildew colonization, necrotic spots
		INSI19058	leaf	*Solanum lycopersicum*	Solanaceae	leaves with powdery mildew colonization, necrotic spots
31	Eggplant mottled dwarf alphanucleorhabdovirus	INSI20038	leaf	*Solanum lycopersicum*	Solanaceae	necrotic spots on leaves
		INSI20038F	fruit	*Solanum lycopersicum*	Solanaceae	fruit mottling, showing circular yellow dents
		INSI20039	leaf	*Solanum lycopersicum*	Solanaceae	necrotic spots on leaves
		INSI20039F	fruit	*Solanum lycopersicum*	Solanaceae	fruit mottling, showing circular yellow dents

32	Physostegia chlorotic mottle alphanucleorhabdovirus	INSI19009	leaf	*Solanum lycopersicum*	Solanaceae	fruit and leaf mottling and yellowing, leaf folding
		INSI20177	leaf	*Solanum lycopersicum*	Solanaceae	leaf folding
		INSI20177F	fruit	*Solanum lycopersicum*	Solanaceae	fruit deformation, cracking
		INSI20239F	fruit	*Solanum lycopersicum*	Solanaceae	fruit and leaf mottling and yellowing
		INSI20242F	fruit	*Solanum lycopersicum*	Solanaceae	fruit and leaf mottling and yellowing
33	Tomato fruit blotch virus	INSI19101	leaf	*Solanum lycopersicum*	Solanaceae	leaf yellowing with necrotic lesions
		INSI19122	leaf	*Solanum lycopersicum*	Solanaceae	leaf yellowing with necrotic lesions
34	Solanum nigrum ilarvirus 1	INSI19127	leaf	*Solanum lycopersicum*	Solanaceae	none (asymptomatic)
		INSI19133	leaf	*Solanum lycopersicum*	Solanaceae	none (asymptomatic)
		INSI19134	leaf	*Solanum lycopersicum*	Solanaceae	mild yellowing and leaf twisting and deformations
		INSI20216	leaf	*Physalis* sp.	Solanaceae	uneven leaf yellowing
35	Tomato matilda virus	INSI19073	leaf	*Solanum lycopersicum*	Solanaceae	none (asymptomatic)
		INSI19085	leaf	*Solanum lycopersicum*	Solanaceae	none (asymptomatic)
		INSI19086	leaf	*Solanum lycopersicum*	Solanaceae	none (asymptomatic)
		INSI19087	leaf	*Solanum lycopersicum*	Solanaceae	none (asymptomatic)
		INSI19088	leaf	*Solanum lycopersicum*	Solanaceae	none (asymptomatic)
		INSI19089	leaf	*Solanum lycopersicum*	Solanaceae	none (asymptomatic)
		INSI19091	leaf	*Solanum lycopersicum*	Solanaceae	none (asymptomatic)
		INSI19092	leaf	*Solanum lycopersicum*	Solanaceae	none (asymptomatic)
		INSI19093	leaf	*Solanum lycopersicum*	Solanaceae	none (asymptomatic)
		INSI19081	leaf	*Solanum lycopersicum*	Solanaceae	corky symptoms on fruits, leaf yellwoing
		INSI19094	leaf	*Solanum lycopersicum*	Solanaceae	yellow spots on leaves
		INSI19103	leaf	*Solanum lycopersicum*	Solanaceae	none (asymptomatic)
		INSI19104	leaf	*Solanum lycopersicum*	Solanaceae	none (asymptomatic)
		INSI19106	leaf	*Solanum lycopersicum*	Solanaceae	none (asymptomatic)
		INSI19108	leaf	*Solanum lycopersicum*	Solanaceae	none (asymptomatic)
		INSI19110	leaf	*Solanum lycopersicum*	Solanaceae	none (asymptomatic)
		INSI19111	leaf	*Solanum lycopersicum*	Solanaceae	none (asymptomatic)
		INSI19112	leaf	*Solanum lycopersicum*	Solanaceae	none (asymptomatic)
		INSI19113	leaf	*Solanum lycopersicum*	Solanaceae	none (asymptomatic)
		INSI19100	leaf	*Solanum lycopersicum*	Solanaceae	leaf yellowing with necrotic lesions
		INSI19101	leaf	*Solanum lycopersicum*	Solanaceae	leaf yellowing with necrotic lesions
		INSI19114	leaf	*Solanum lycopersicum*	Solanaceae	necrotic leaf lesions
		INSI19115	leaf	*Solanum lycopersicum*	Solanaceae	leaf chlorosis with necrotic lesions

INSI19116	leaf	*Solanum lycopersicum*	Solanaceae	leaf yellowing with necrotic lesions
INSI19117	leaf	*Solanum lycopersicum*	Solanaceae	leaf yellowing with necrotic lesions
INSI19122	leaf	*Solanum lycopersicum*	Solanaceae	leaf yellowing with necrotic lesions
INSI19126	leaf	*Solanum lycopersicum*	Solanaceae	leaf yellowing with necrotic lesions
INSI19128	leaf	*Solanum lycopersicum*	Solanaceae	leaf yellowing with necrotic lesions
INSI19129	leaf	*Solanum lycopersicum*	Solanaceae	yellow spots on leaves
INSI19134	leaf	*Solanum lycopersicum*	Solanaceae	mild yellowing and leaf twisting and deformations
INSI19135	leaf	*Solanum lycopersicum*	Solanaceae	leaf yellowing with necrotic lesions
INSI19127	leaf	*Solanum lycopersicum*	Solanaceae	none (asymptomatic)
INSI19130	leaf	*Solanum lycopersicum*	Solanaceae	none (asymptomatic)
INSI19131	leaf	*Solanum lycopersicum*	Solanaceae	none (asymptomatic)
INSI19132	leaf	*Solanum lycopersicum*	Solanaceae	none (asymptomatic)
INSI19133	leaf	*Solanum lycopersicum*	Solanaceae	none (asymptomatic)
INSI19141	leaf	*Solanum lycopersicum*	Solanaceae	none (asymptomatic)
INSI19142	leaf	*Solanum lycopersicum*	Solanaceae	none (asymptomatic)
INSI19143	leaf	*Solanum lycopersicum*	Solanaceae	none (asymptomatic)
INSI19144	leaf	*Solanum lycopersicum*	Solanaceae	none (asymptomatic)
INSI19147	leaf	*Solanum lycopersicum*	Solanaceae	none (asymptomatic)
INSI19148	leaf	*Solanum lycopersicum*	Solanaceae	none (asymptomatic)
INSI19139	leaf	*Solanum lycopersicum*	Solanaceae	leaf yellowing with necrotic lesions
INSI19149	leaf	*Solanum lycopersicum*	Solanaceae	leaf yellowing
INSI19151	leaf	*Solanum lycopersicum*	Solanaceae	none (asymptomatic)
INSI19154	leaf	*Solanum lycopersicum*	Solanaceae	leaf mottling, dwarfing and twisting
INSI19155	leaf	*Solanum lycopersicum*	Solanaceae	leaf mottling, dwarfing and twisting
INSI19140	leaf	*Chenopodium* sp.	Chenopodiaceae	yellow spots on leaves
INSI19150	leaf	*Chenopodium* sp.	Chenopodiaceae	yellow spots on leaves
INSI19157	leaf	*Erigeron annuus*	Asteraceae	necrotic lesions on leaves
INSI19158	leaf	*Ranunculus repens*	Ranunculaceae	leaf mosaic
INSI20040	leaf	*Solanum lycopersicum*	Solanaceae	none (asymptomatic)
INSI20041	leaf	*Solanum lycopersicum*	Solanaceae	none (asymptomatic)
INSI20042	leaf	*Solanum lycopersicum*	Solanaceae	none (asymptomatic)
INSI20043	leaf	*Solanum lycopersicum*	Solanaceae	none (asymptomatic)
INSI20044	leaf	*Solanum lycopersicum*	Solanaceae	none (asymptomatic)

		INSI20030	leaf	*Solanum lycopersicum*	Solanaceae	necrotic leaf spots
		INSI20031	leaf	*Solanum lycopersicum*	Solanaceae	necrotic leaf spots
		INSI20033	leaf	*Solanum lycopersicum*	Solanaceae	necrotic leaf spots
		INSI20035	leaf	*Solanum lycopersicum*	Solanaceae	purpling of leaf lamina
		INSI20036	leaf	*Solanum lycopersicum*	Solanaceae	interveinal leaf yellowing
		INSI20037	leaf	*Solanum lycopersicum*	Solanaceae	necrotic leaf spots, necrosis on leaf lamina
		INSI20038	leaf	*Solanum lycopersicum*	Solanaceae	necrotic leaf spots, necrosis on leaf lamina
		INSI20055	leaf	*Solanum lycopersicum*	Solanaceae	purpling of leaf lamina
		INSI20108	leaf	*Solanum lycopersicum*	Solanaceae	leaf deformation
		INSI20126	leaf	*Solanum lycopersicum*	Solanaceae	necrotic leaf spots
		INSI20127	leaf	*Solanum lycopersicum*	Solanaceae	necrotic leaf spots, necrosis on leaf lamina
		INSI20128	leaf	*Solanum lycopersicum*	Solanaceae	necrotic leaf spots, necrosis on leaf lamina
		INSI20129	leaf	*Solanum lycopersicum*	Solanaceae	necrotic leaf spots, necrosis on leaf lamina
		INSI20132	leaf	*Solanum lycopersicum*	Solanaceae	leaf deformation and dwarfing
36	Taraxacum viroid-like circular RNA 1	INSI20252	leaf	*Taraxacum officinale*	Asteraceae	interveinal leaf chlorosis
