# Supplementary material for: In-depth study of tomato and weed viromes reveals undiscovered plant virus diversity in an agroecosystem
Source: Microbiome. 2023 Mar 28;11:60. doi: 10.1186/s40168-023-01500-6 (PMC10042675; doi:10.1186/s40168-023-01500-6)
Supplement: Supplementary file 6 — Additional file 5: Supplementary Figure 3. Representative field photos of confirmed associated plant hosts of selected viruses and putative viroids. [file 40168_2023_1500_MOESM5_ESM.pdf]

In-depth study of tomato and weed viromes reveals undiscovered plant virus diversity in an agroecosystem

Rivarez, MPS[1,2,*], Pecman, A[1,2], Bačnik, K[1,2], Maksimović Carvalho Ferreira, O[1,2], Vučurović, A[1], Seljak, G[1], Mehle, N[1,3], Gutiérrez-Aguirre, I[1], Ravnikar, M[1], and Kutnjak, D[1,*]

[1]Department of Biotechnology and Systems Biology, National Institute of Biology, Večna pot 111, Ljubljana, 1000 Slovenia. [2]Jožef Stefan International Postgraduate School, Jamova cesta 39, Ljubljana, 1000 Slovenia. [3]School for Viticulture and Enology, University of Nova Gorica, Dvorec Lanthieri Glavni trg 8, Vipava, 5271 Slovenia. *for correspondence email Denis Kutnjak (denis.kutnjak@nib.si) and Mark Paul Selda Rivarez (mpsrivarez@gmail.com)

SUPPLEMENTARY INFORMATION

Additional File 05

Supplementary Figure 3. Representative field photos of confirmed associated plant hosts of selected viruses and putative viroids.

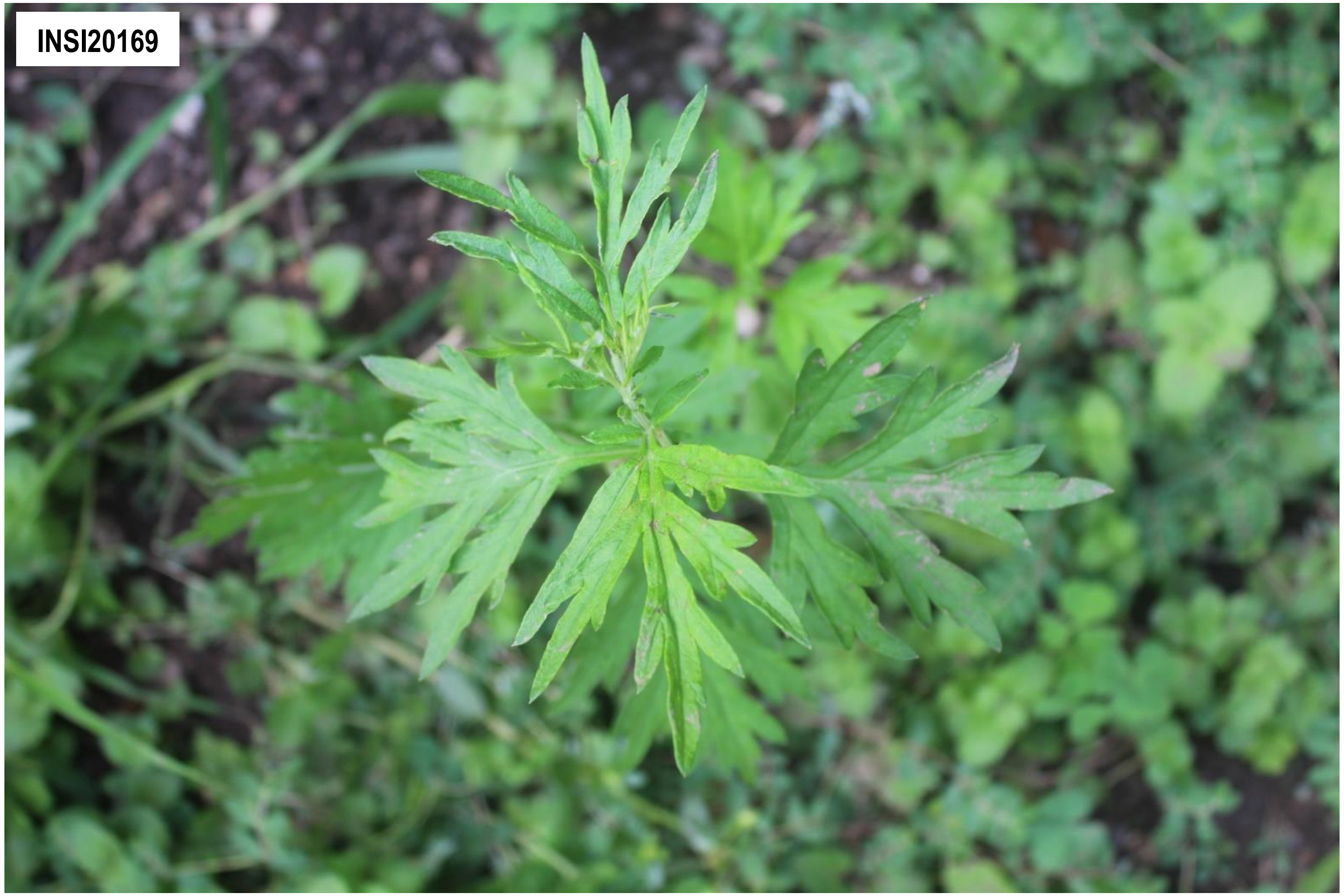

Supplementary Figure 3-01. Artermisia fimovirus 1. The confirmed associated plant host(s) shown is/are *Artemisia verlotiorum* (Asteraceae).

Supplementary Figure 3. Representative field photos of confirmed associated plant hosts of selected viruses and putative viroids. **Note**: There are other viruses detected in the sequencing pools (*i.e.* composite sample) where the samples shown belong, which implies possible co-infection of viruses on the specific sample shown. Thus, it is difficult to associate the symptom phenotype with the detection of a single specific virus. Complete information on the individual samples can be found in Supplementary Table 1 and the description of symptoms of the associated plant hosts are summarized in Supplementary Table 9.

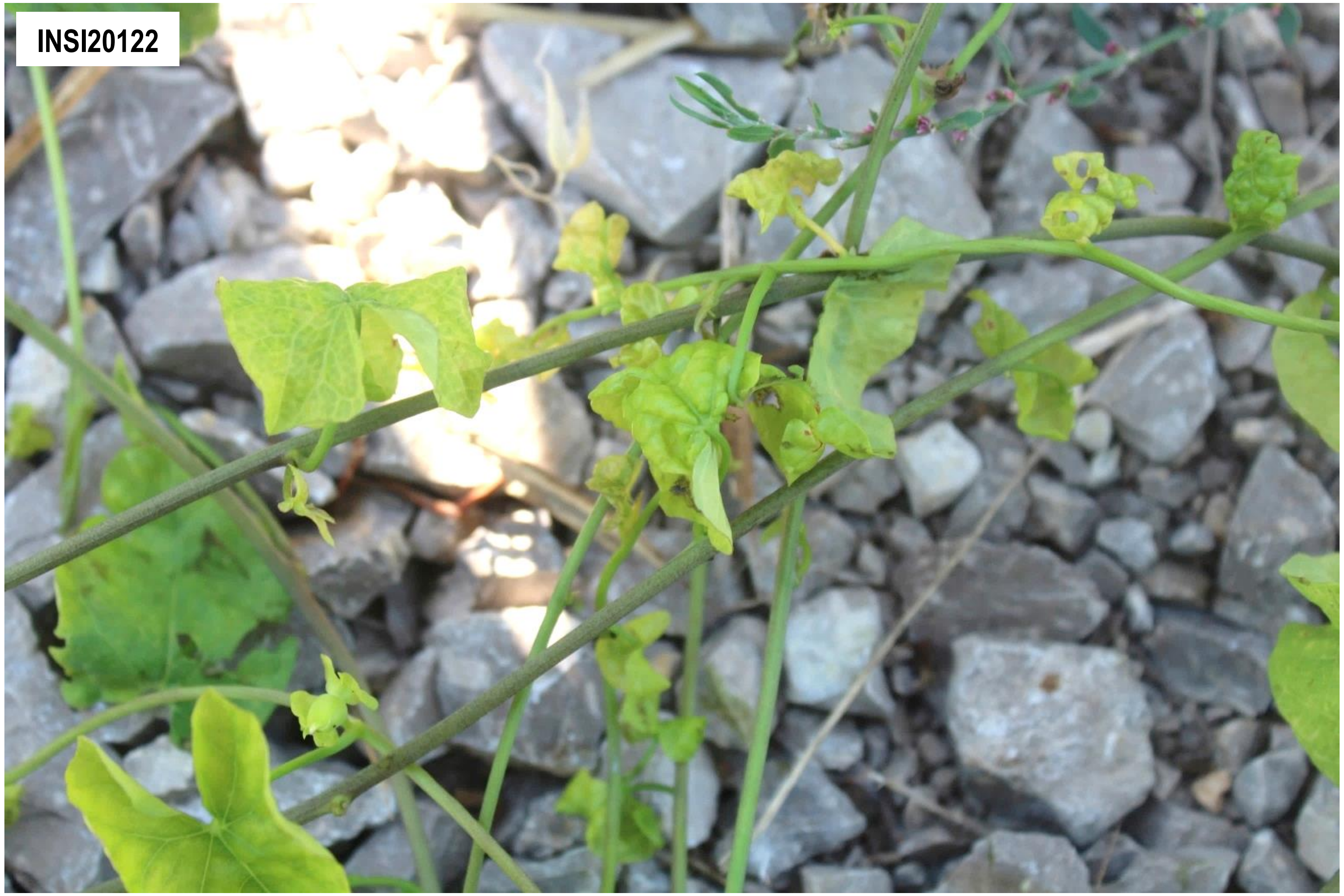

Supplementary Figure 3-02. Calystegia geminivirus 1 and Calystegia pelarspovirus 1. The confirmed associated plant host(s) shown is/are *Calystegia sp.* (Convolvulaceae).

Supplementary Figure 3 (continued)**.** Representative field photos of confirmed associated plant hosts of selected viruses and putative viroids. **Note**: There are other viruses detected in the sequencing pools (*i.e.* composite sample) where the samples shown belong, which implies possible co-infection of viruses on the specific sample shown. Thus, it is difficult to associate the symptom phenotype with the detection of a single specific virus. Complete information on the individual samples can be found in Supplementary Table 1 and the description of symptoms of the associated plant hosts are summarized in Supplementary Table 9.

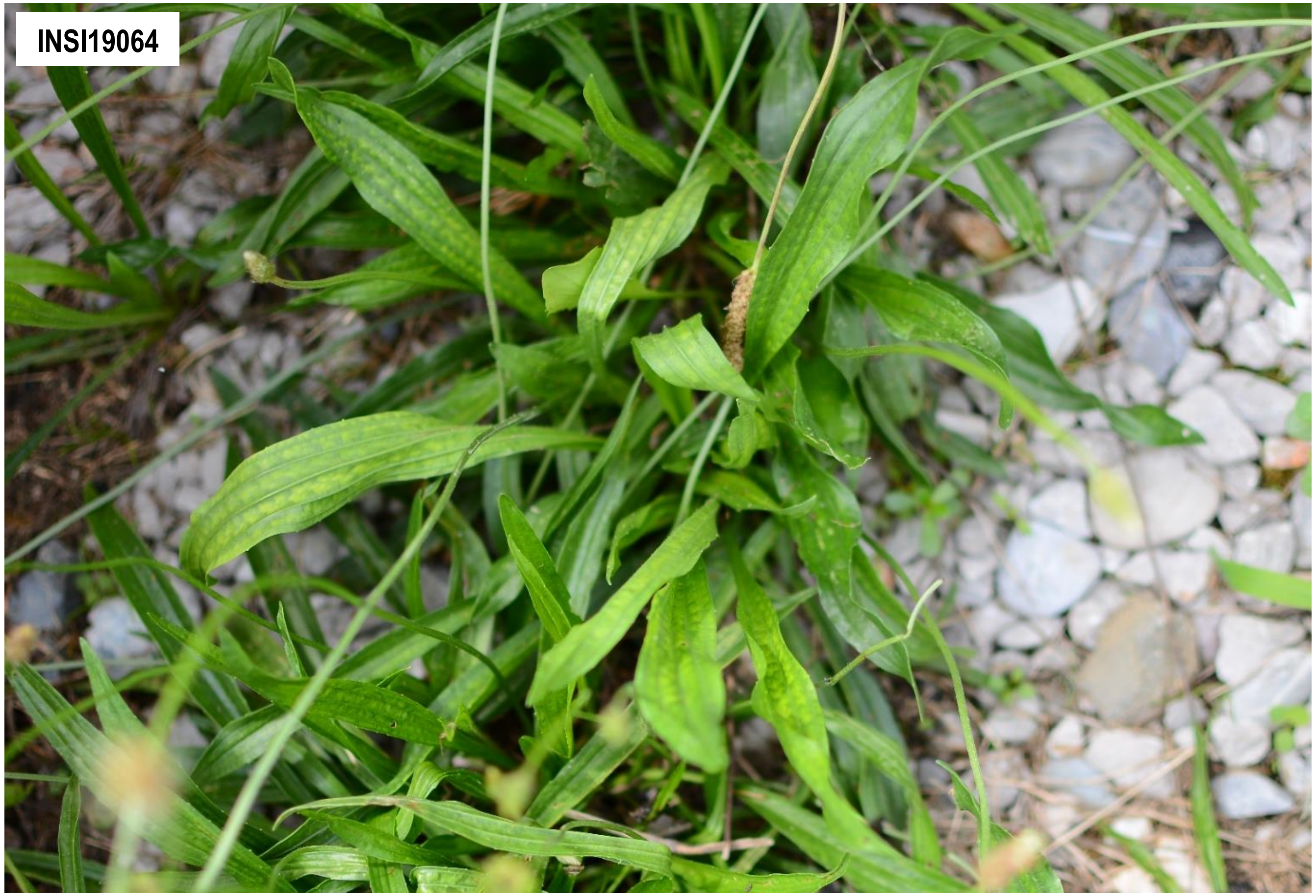

Supplementary Figure 3-03. Plantago potyvirus 1. The confirmed associated plant host(s) shown is/are *Plantago lanceolata* (Plantaginaceae).

Supplementary Figure 3 (continued)**.** Representative field photos of confirmed associated plant hosts of selected viruses and putative viroids. **Note**: There are other viruses detected in the sequencing pools (*i.e.* composite sample) where the samples shown belong, which implies possible co-infection of viruses on the specific sample shown. Thus, it is difficult to associate the symptom phenotype with the detection of a single specific virus. Complete information on the individual samples can be found in Supplementary Table 1 and the description of symptoms of the associated plant hosts are summarized in Supplementary Table 9.

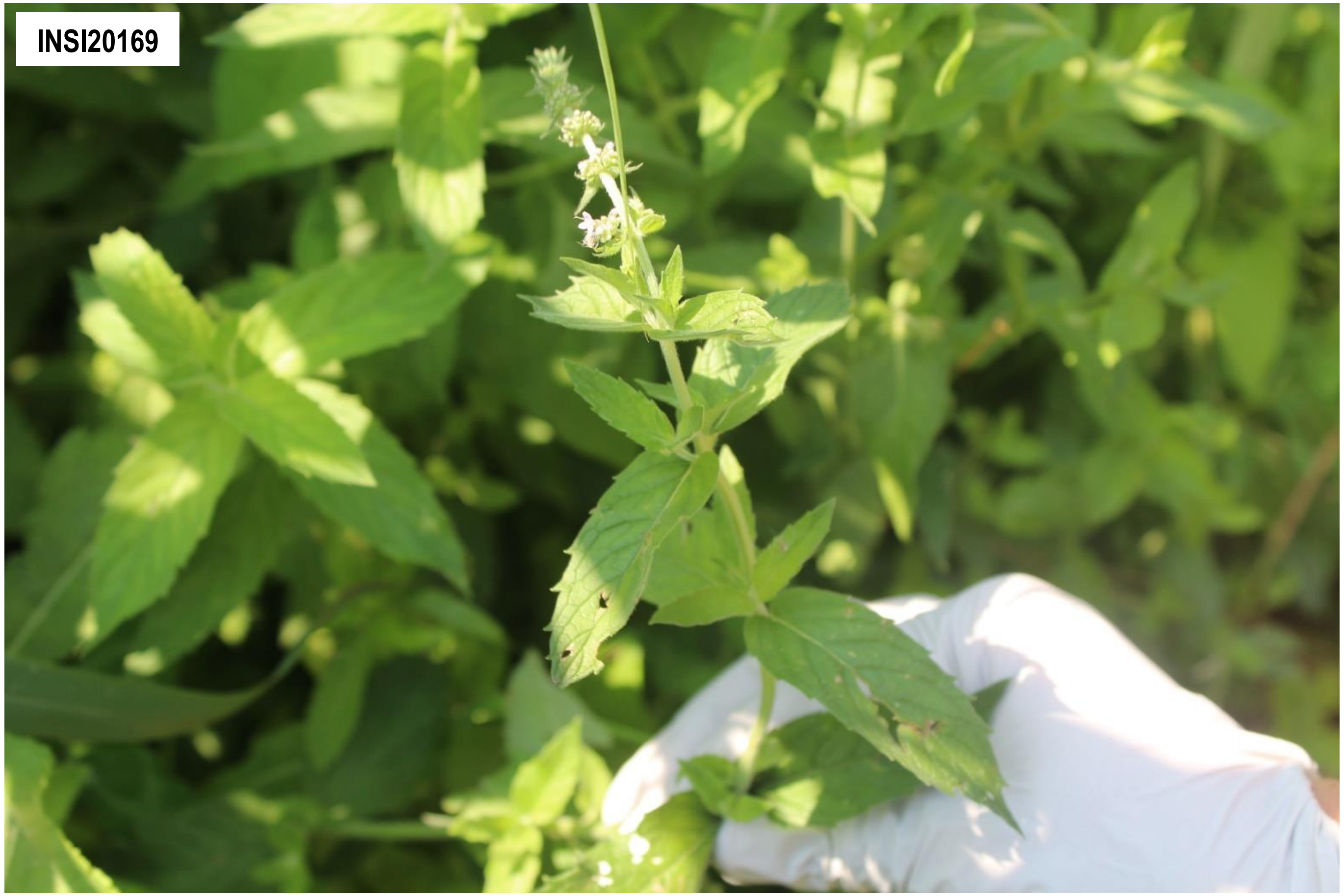

Supplementary Figure 3-04. Mentha macluravirus 1. The confirmed associated plant host(s) shown is/are *Mentha spicata* (Lamiaceae).

Supplementary Figure 3 (continued)**.** Representative field photos of confirmed associated plant hosts of selected viruses and putative viroids. **Note**: There are other viruses detected in the sequencing pools (*i.e.* composite sample) where the samples shown belong, which implies possible co-infection of viruses on the specific sample shown. Thus, it is difficult to associate the symptom phenotype with the detection of a single specific virus. Complete information on the individual samples can be found in Supplementary Table 1 and the description of symptoms of the associated plant hosts are summarized in Supplementary Table 9.

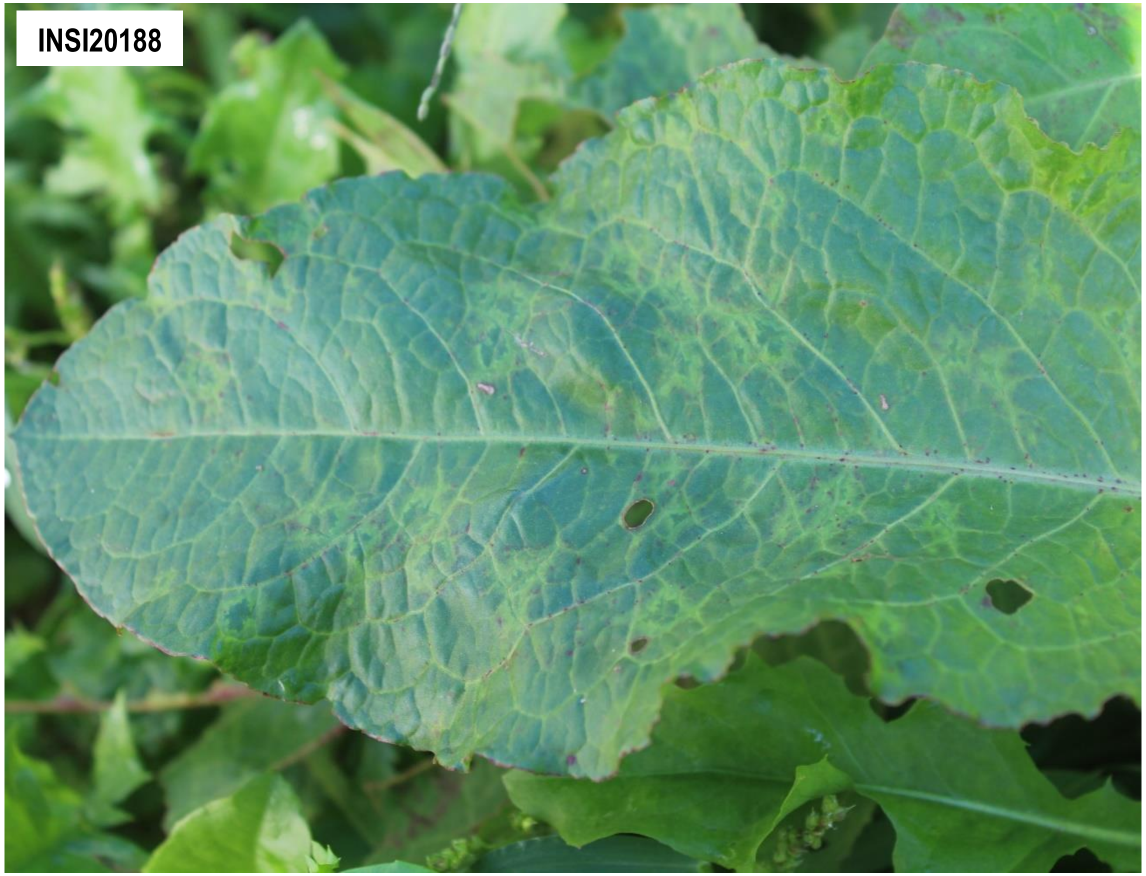

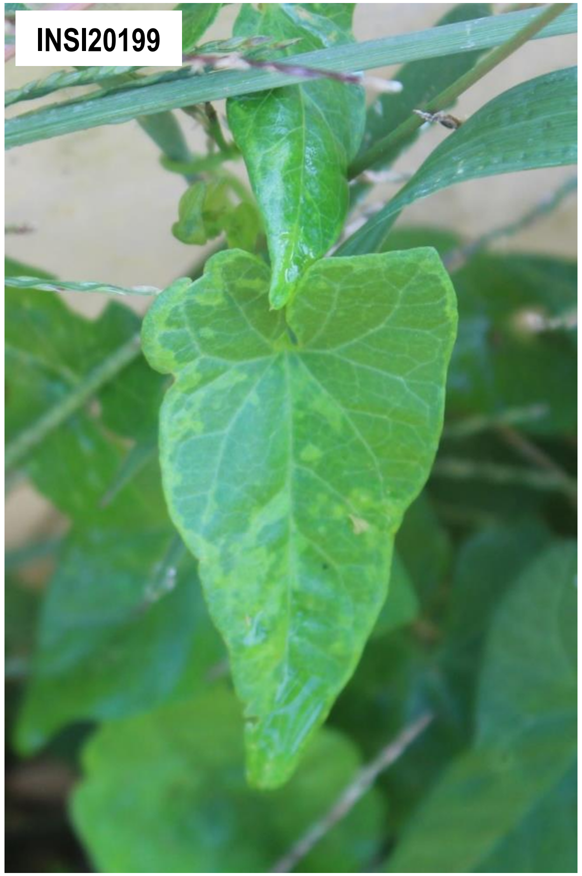

Supplementary Figure 3-05. Rumex potyvirus 1. The confirmed associated plant host(s) shown is/are *Rumex* sp. (Polygonaceae) (INSI20188) and *Convolvulus* sp. (Convolvulaceae) (INSI20199).

Supplementary Figure 3 (continued)**.** Representative field photos of confirmed associated plant hosts of selected viruses and putative viroids. **Note**: There are other viruses detected in the sequencing pools (*i.e.* composite sample) where the samples shown belong, which implies possible co-infection of viruses on the specific sample shown. Thus, it is difficult to associate the symptom phenotype with the detection of a single specific virus. Complete information on the individual samples can be found in Supplementary Table 1 and the description of symptoms of the associated plant hosts are summarized in Supplementary Table 9.

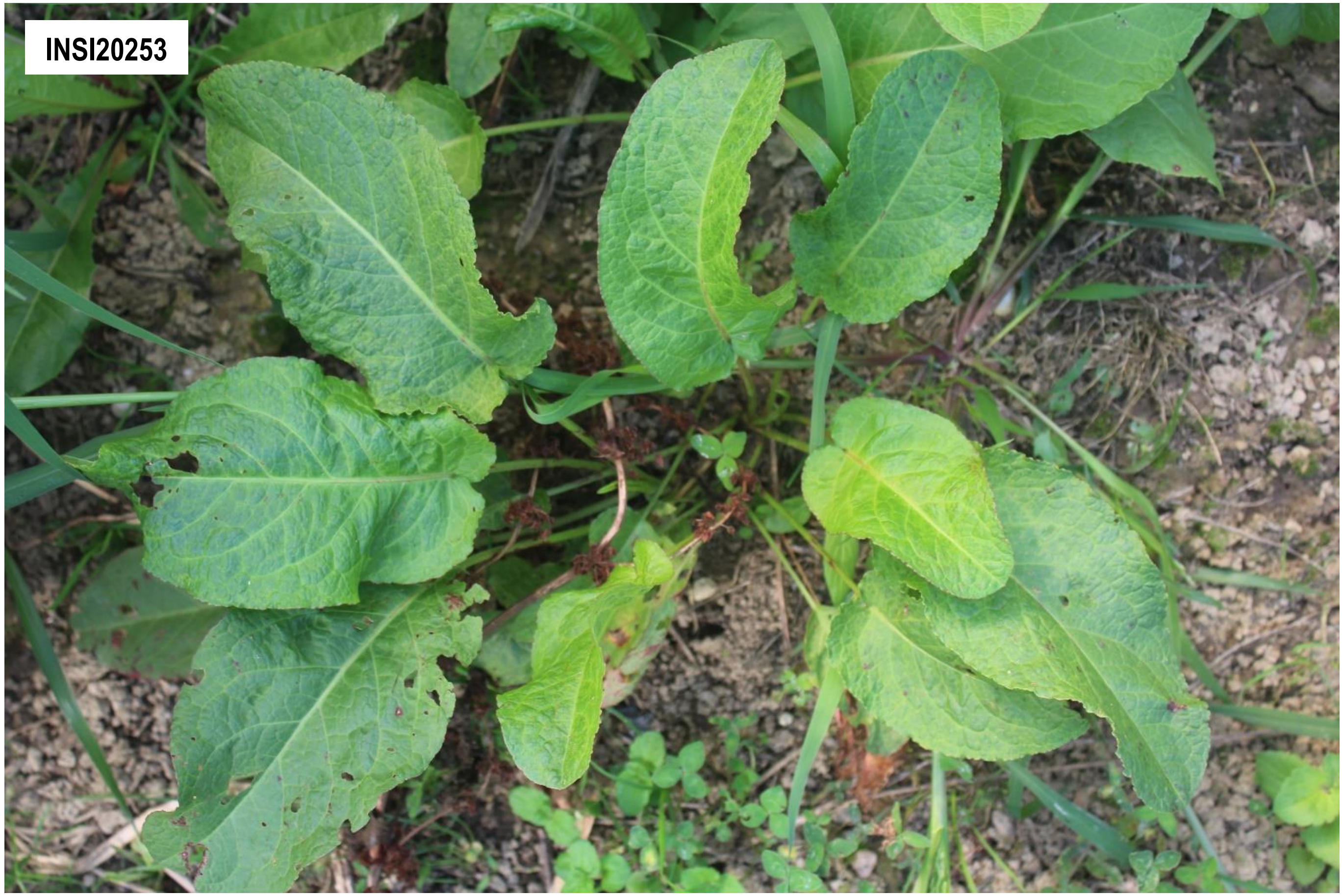

Supplementary Figure 3-06. Broad-leafed dock virus A, isolate 2. The confirmed associated plant host(s) shown is/are *Rumex crispus* (Polygonaceae).

Supplementary Figure 3 (continued)**.** Representative field photos of confirmed associated plant hosts of selected viruses and putative viroids. **Note**: There are other viruses detected in the sequencing pools (*i.e.* composite sample) where the samples shown belong, which implies possible co-infection of viruses on the specific sample shown. Thus, it is difficult to associate the symptom phenotype with the detection of a single specific virus. Complete information on the individual samples can be found in Supplementary Table 1 and the description of symptoms of the associated plant hosts are summarized in Supplementary Table 9.

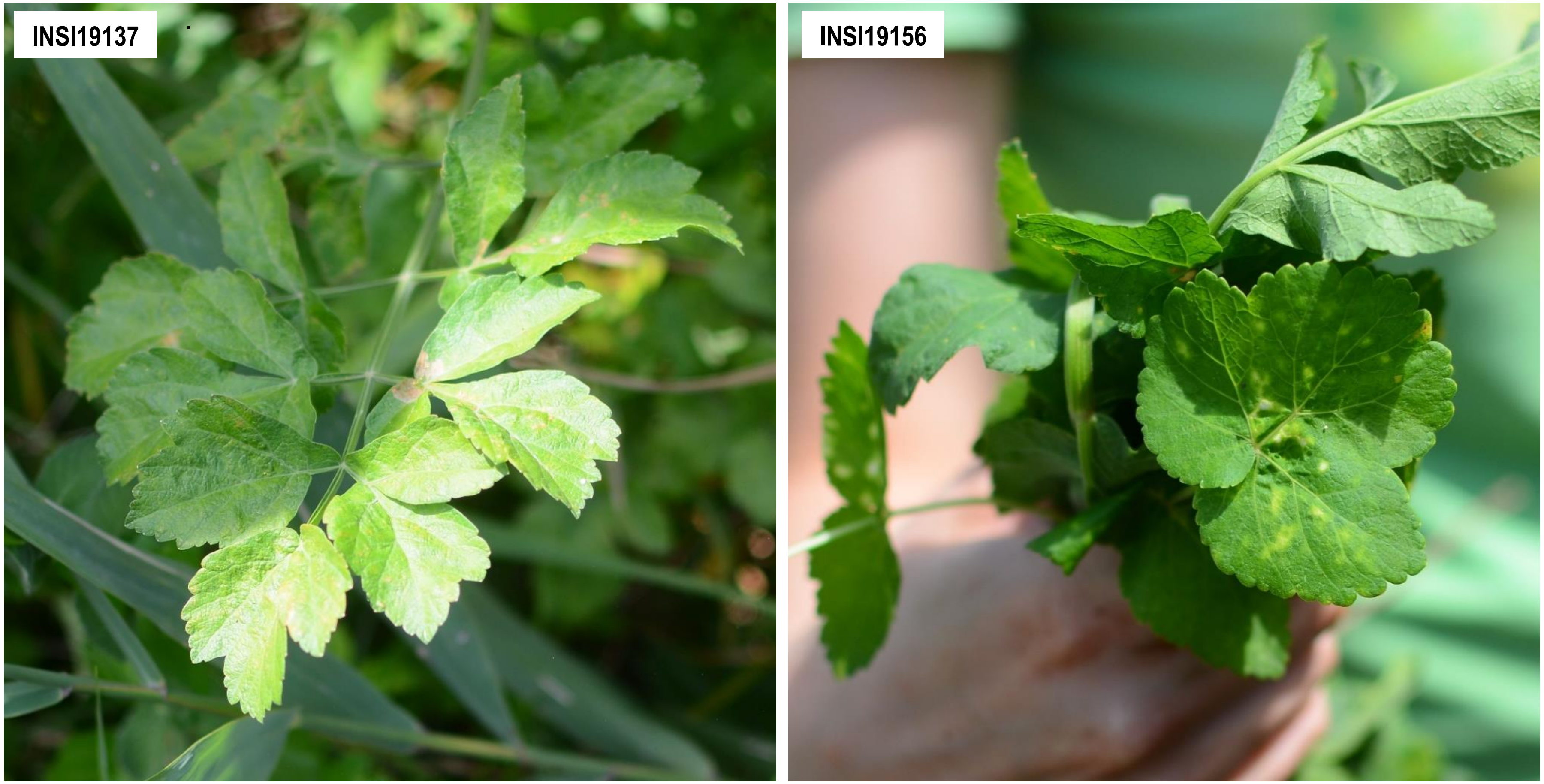

Supplementary Figure 3-07. Pastinaca umbravirus 1. The confirmed associated plant host(s) shown is/are *Pastinaca sativa* (Apiaceae). Sample INSI19137 is also the confirmed associated plant host of Pastinaca potexvirus 1 and Pastinaca cytorhabdovirus 1.

Supplementary Figure 3 (continued)**.** Representative field photos of confirmed associated plant hosts of selected viruses and putative viroids. **Note**: There are other viruses detected in the sequencing pools (*i.e.* composite sample) where the samples shown belong, which implies possible co-infection of viruses on the specific sample shown. Thus, it is difficult to associate the symptom phenotype with the detection of a single specific virus. Complete information on the individual samples can be found in Supplementary Table 1 and the description of symptoms of the associated plant hosts are summarized in Supplementary Table 9.

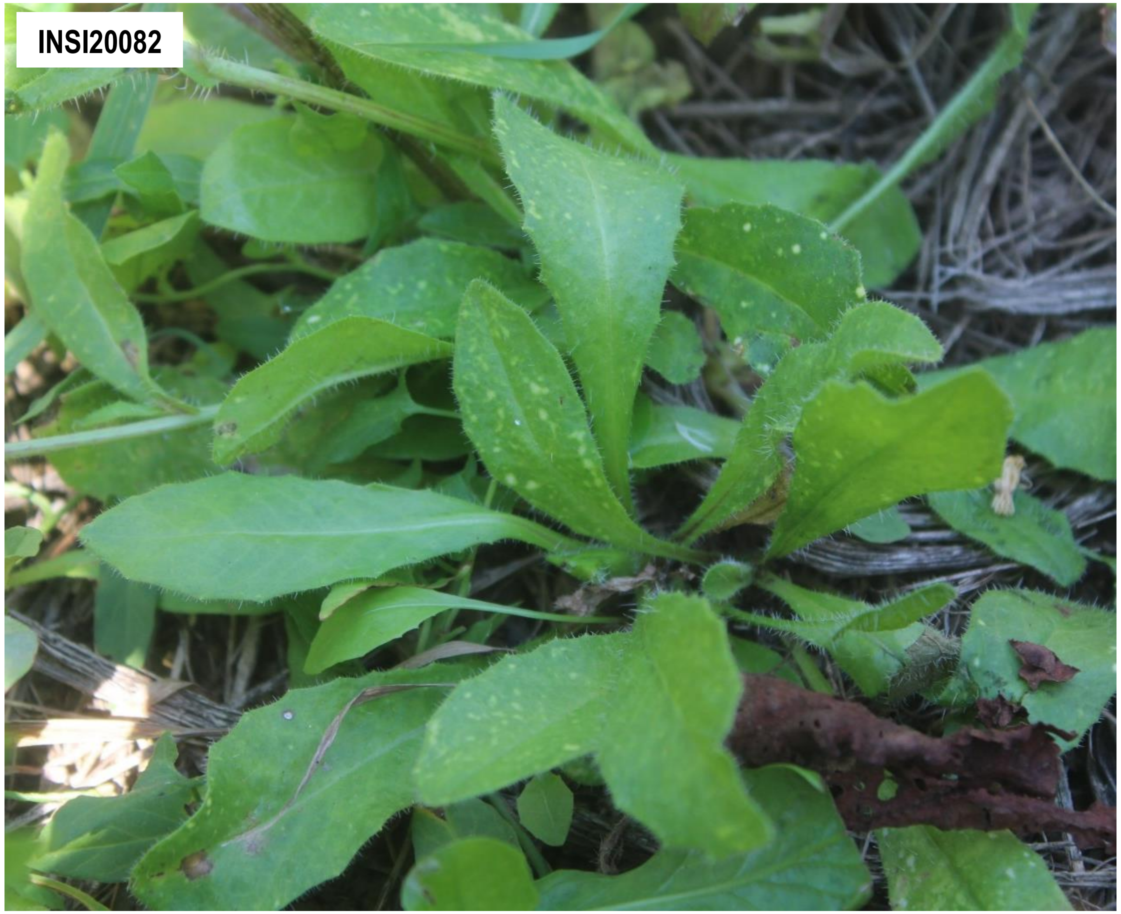

Supplementary Figure 3-08. Picris umbravirus 1. The confirmed associated plant host(s) shown is/are *Picris echoides* (Asteraceae).
The sample shown is also confirmed associated host of Cichorium alphacarmovirus 1 and Picris cytorhabdovirus 1.

Supplementary Figure 3 (continued)**.** Representative field photos of confirmed associated plant hosts of selected viruses and putative viroids. **Note**: There are other viruses detected in the sequencing pools (*i.e.* composite sample) where the samples shown belong, which implies possible co-infection of viruses on the specific sample shown. Thus, it is difficult to associate the symptom phenotype with the detection of a single specific virus. Complete information on the individual samples can be found in Supplementary Table 1 and the description of symptoms of the associated plant hosts are summarized in Supplementary Table 9.

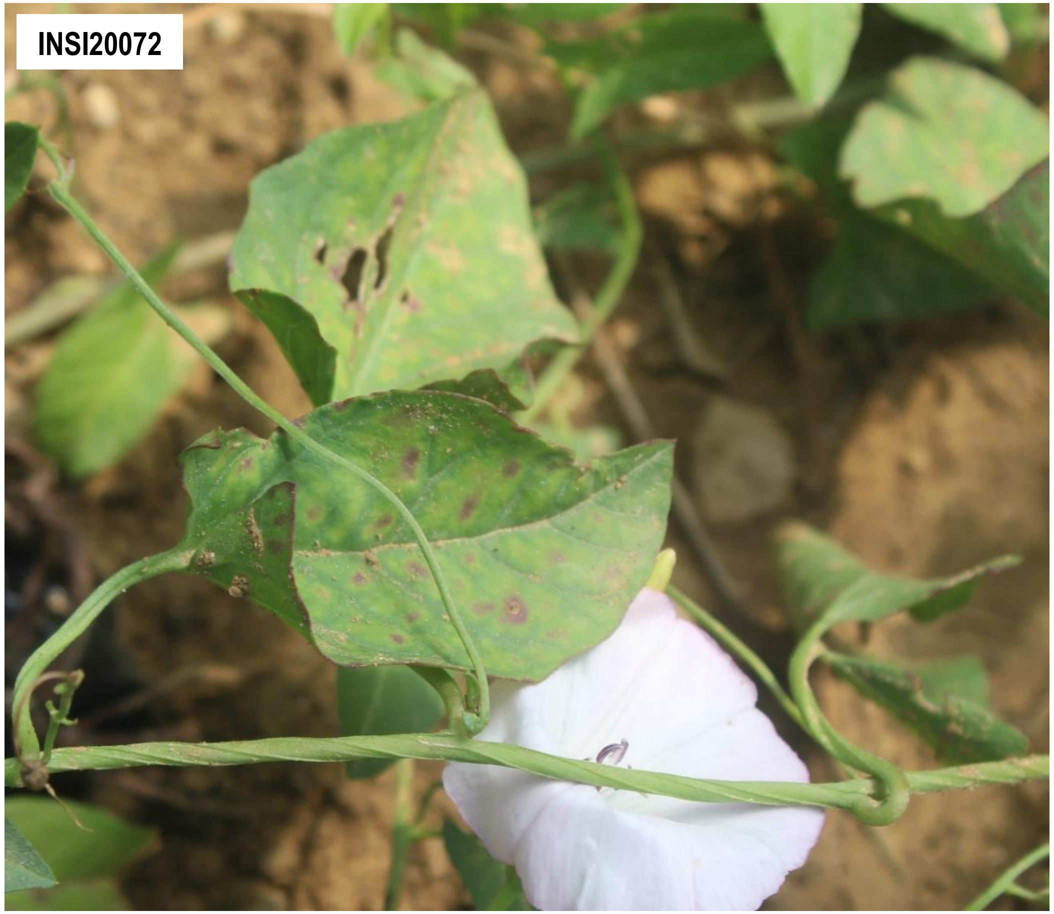

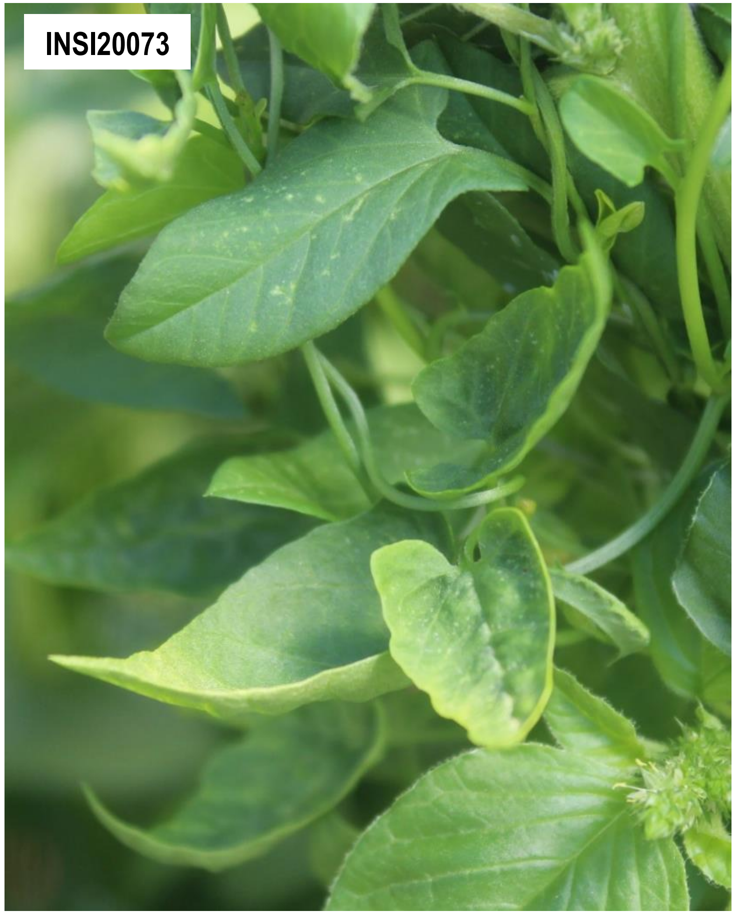

Supplementary Figure 3-09. Convolvulus aureusvirus 1. The confirmed associated plant host(s) shown is/are *Convolvulus arvensis* (Convolvulaceae).

Supplementary Figure 3 (continued)**.** Representative field photos of confirmed associated plant hosts of selected viruses and putative viroids. **Note**: There are other viruses detected in the sequencing pools (*i.e.* composite sample) where the samples shown belong, which implies possible co-infection of viruses on the specific sample shown. Thus, it is difficult to associate the symptom phenotype with the detection of a single specific virus. Complete information on the individual samples can be found in Supplementary Table 1 and the description of symptoms of the associated plant hosts are summarized in Supplementary Table 9.

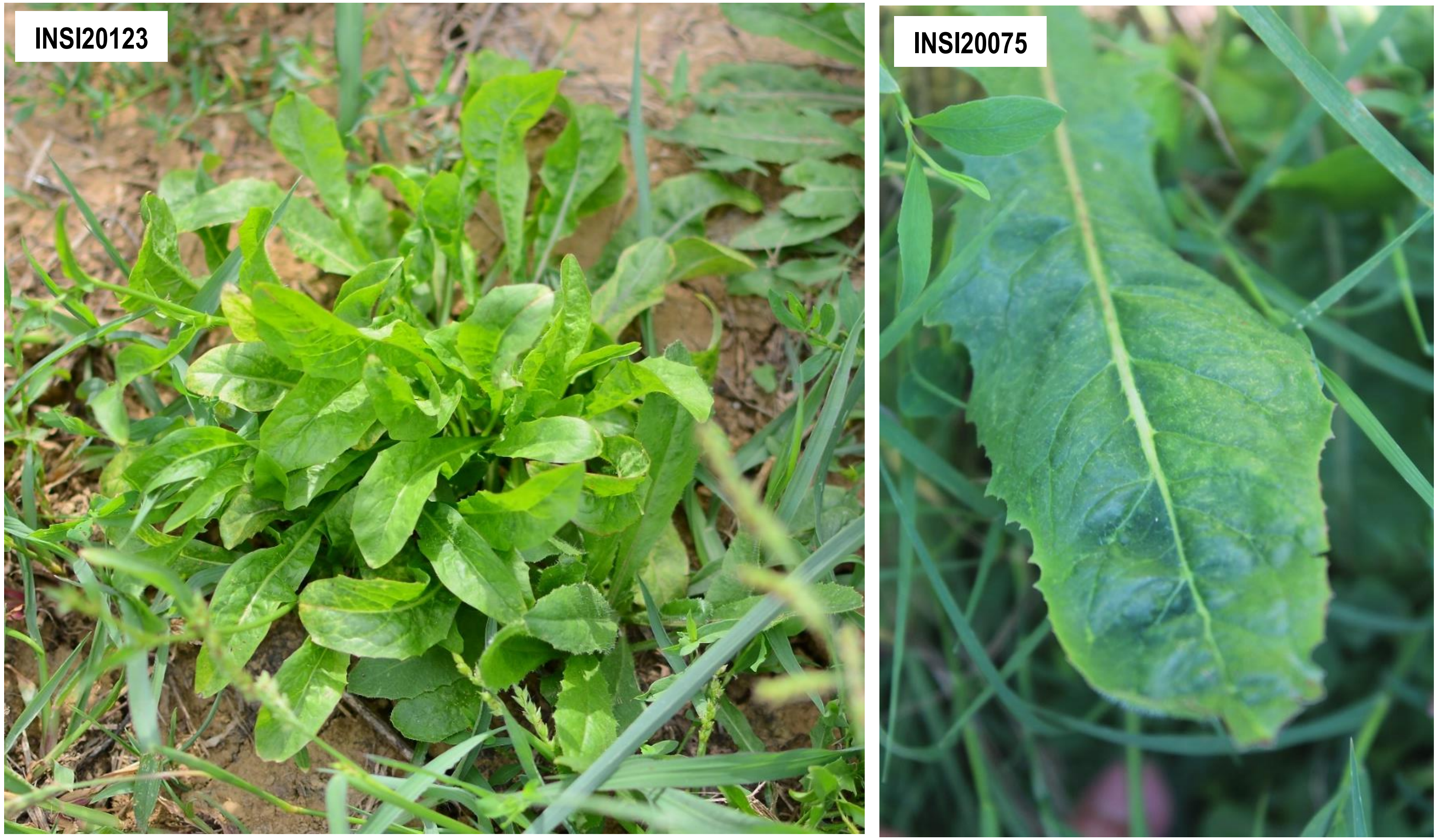

Supplementary Figure 3-10. Cichorium alphacarmovirus 1. The confirmed associated plant host(s) shown is/are *Calystegia* sp. (Convolvulaceae).

Supplementary Figure 3 (continued)**.** Representative field photos of confirmed associated plant hosts of selected viruses and putative viroids. **Note**: There are other viruses detected in the sequencing pools (*i.e.* composite sample) where the samples shown belong, which implies possible co-infection of viruses on the specific sample shown. Thus, it is difficult to associate the symptom phenotype with the detection of a single specific virus. Complete information on the individual samples can be found in Supplementary Table 1 and the description of symptoms of the associated plant hosts are summarized in Supplementary Table 9.

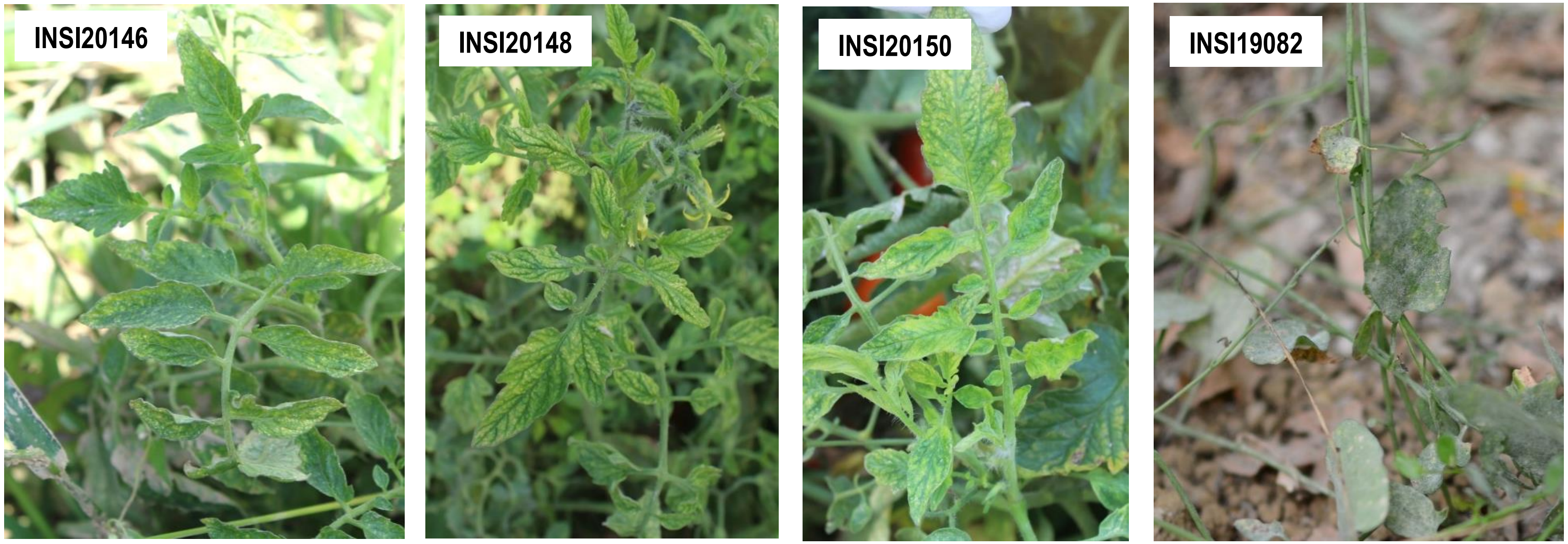

Supplementary FigureS3-11. Plant associated tobamo-like virus 1. The confirmed associated plant host(s) shown is/are *Solanum lycopersicum* (Solanaceae) (INSI20146, INSI20148, INSI20150) and *Convolvulus arvensis* (Convolvulaceae) (INSI19082).

Supplementary Figure 3 (continued)**.** Representative field photos of confirmed associated plant hosts of selected viruses and putative viroids. **Note**: There are other viruses detected in the sequencing pools (*i.e.* composite sample) where the samples shown belong, which implies possible co-infection of viruses on the specific sample shown. Thus, it is difficult to associate the symptom phenotype with the detection of a single specific virus. Complete information on the individual samples can be found in Supplementary Table 1 and the description of symptoms of the associated plant hosts are summarized in Supplementary Table 9.

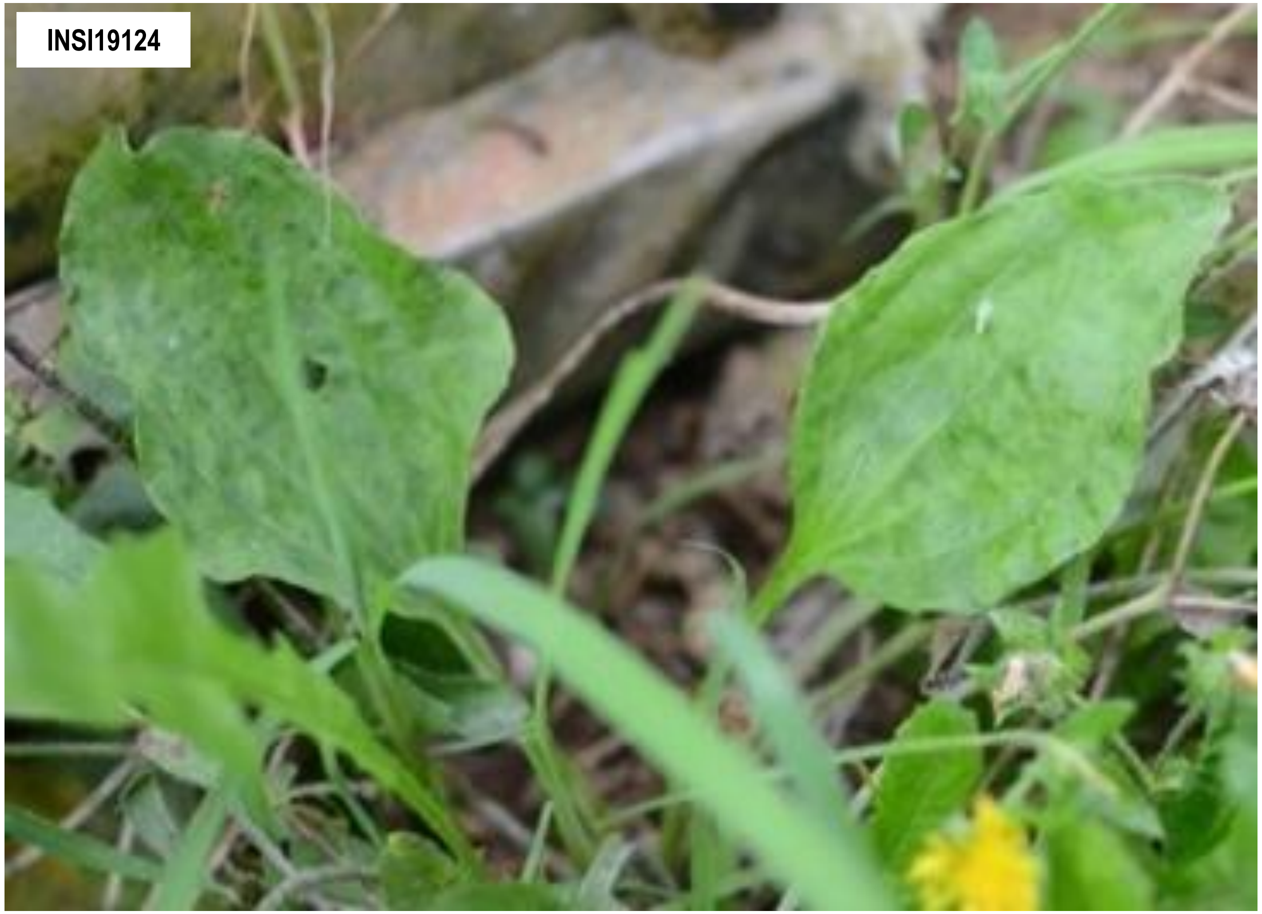

Supplementary Figure 3-12. Plantago tobamovirus 1. The confirmed associated plant host shown is *Plantago major* (Plantaginaceae).

Supplementary Figure 3 (continued)**.** Representative field photos of confirmed associated plant hosts of selected viruses and putative viroids. **Note**: There are other viruses detected in the sequencing pools (*i.e.* composite sample) where the samples shown belong, which implies possible co-infection of viruses on the specific sample shown. Thus, it is difficult to associate the symptom phenotype with the detection of a single specific virus. Complete information on the individual samples can be found in Supplementary Table 1 and the description of symptoms of the associated plant hosts are summarized in Supplementary Table 9.

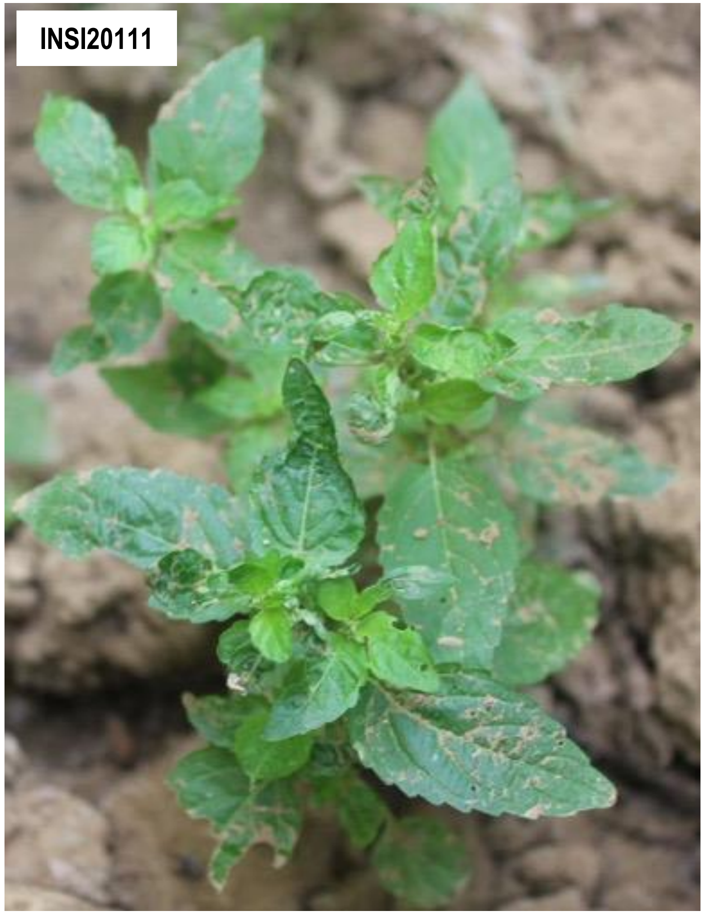

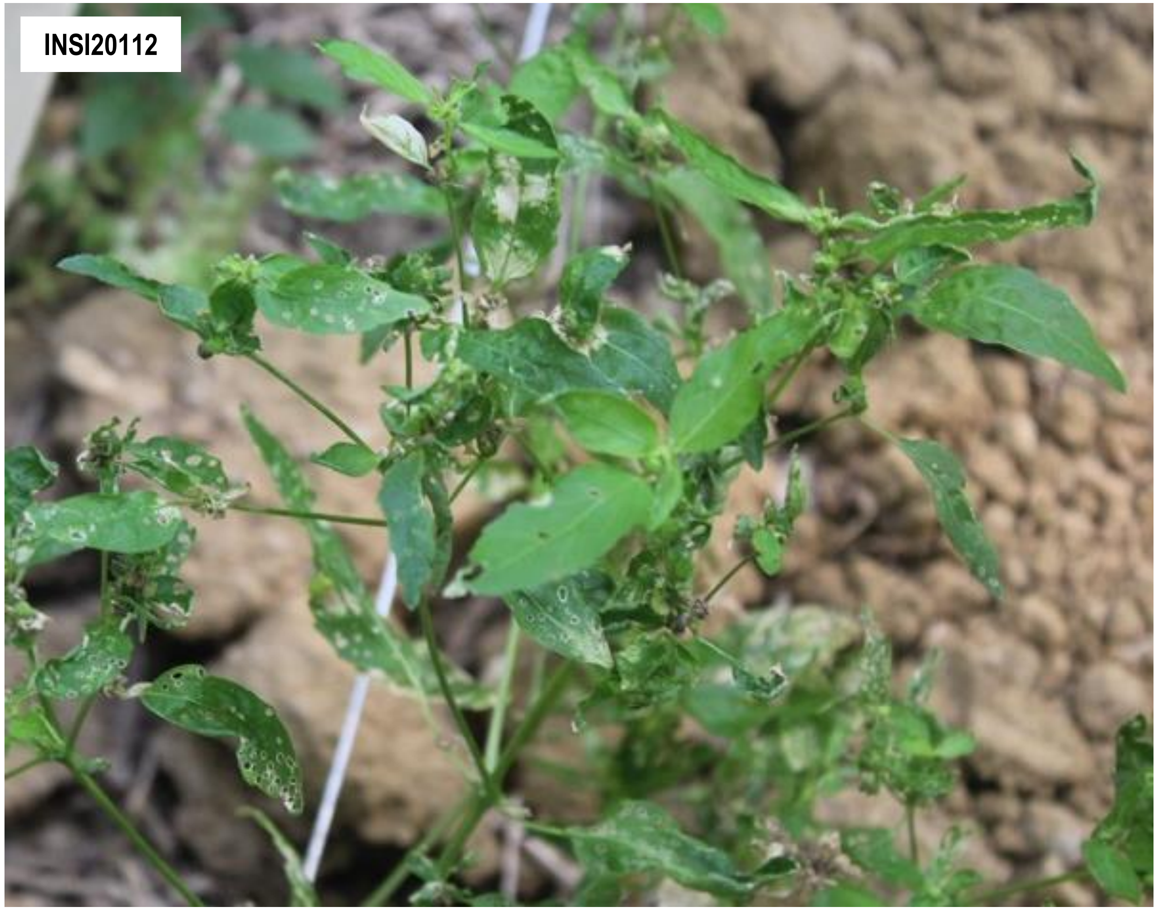

Supplementary Figure 3-13. Mercurialis orthotospovirus 1. The confirmed associated plant hosts shown are *Mercurialis annua*.

Supplementary Figure 3 (continued)**.** Representative field photos of confirmed associated plant hosts of selected viruses and putative viroids. **Note**: There are other viruses detected in the sequencing pools (*i.e.* composite sample) where the samples shown belong, which implies possible co-infection of viruses on the specific sample shown. Thus, it is difficult to associate the symptom phenotype with the detection of a single specific virus. Complete information on the individual samples can be found in Supplementary Table 1 and the description of symptoms of the associated plant hosts are summarized in Supplementary Table 9.

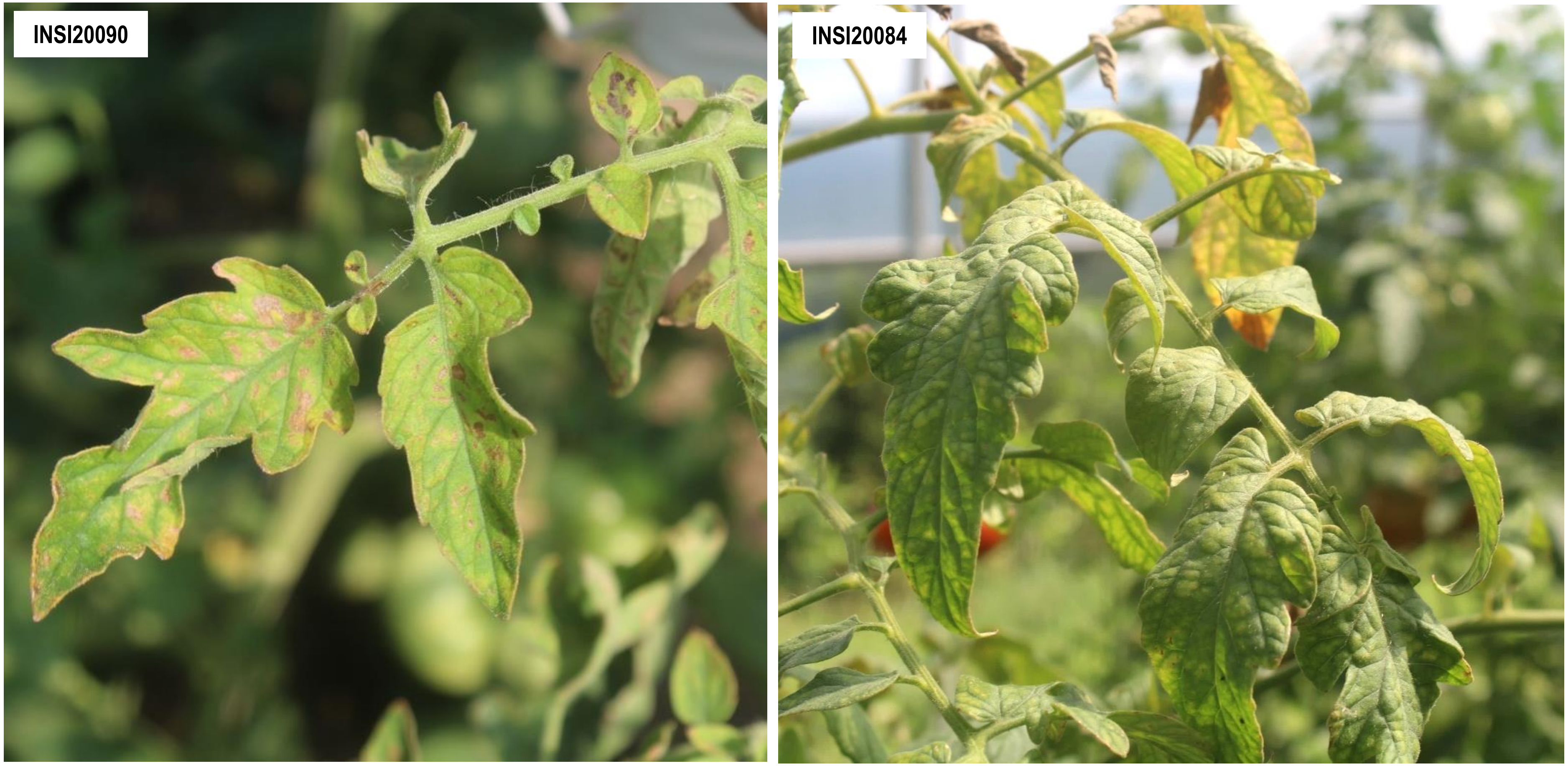

Supplementary Figure 3-14. Tomato associated bunya-like virus 1. The confirmed associated plant host shown is *Solanum lycopersicum*.

Supplementary Figure 3 (continued)**.** Representative field photos of confirmed associated plant hosts of selected viruses and putative viroids. **Note**: There are other viruses detected in the sequencing pools (*i.e.* composite sample) where the samples shown belong, which implies possible co-infection of viruses on the specific sample shown. Thus, it is difficult to associate the symptom phenotype with the detection of a single specific virus. Complete information on the individual samples can be found in Supplementary Table 1 and the description of symptoms of the associated plant hosts are summarized in Supplementary Table 9.

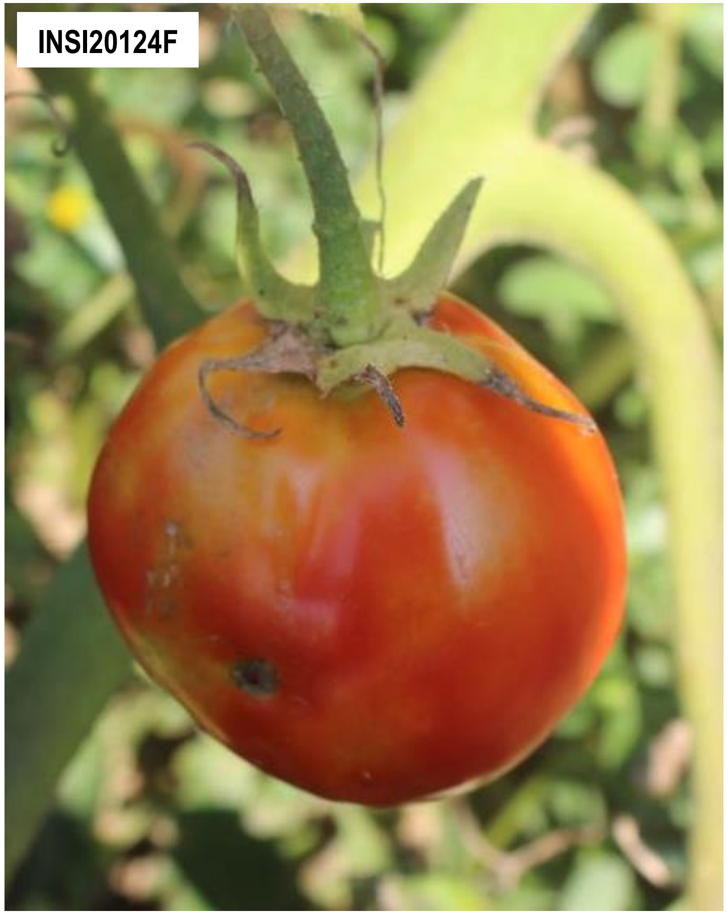

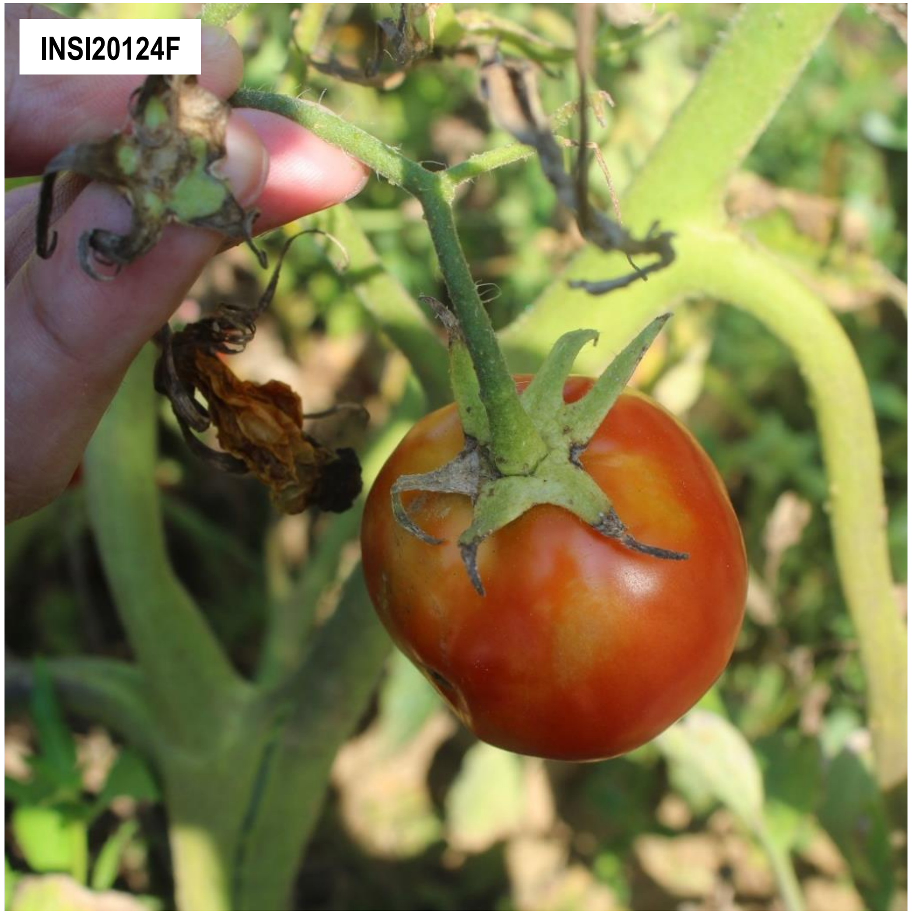

Supplementary Figure 3-15. Tomato vitivirus 1. The confirmed associated plant host(s) shown is/are *Solanum lycopersicum* (Solanaceae).

Supplementary Figure 3 (continued)**.** Representative field photos of confirmed associated plant hosts of selected viruses and putative viroids. **Note**: There are other viruses detected in the sequencing pools (*i.e.* composite sample) where the samples shown belong, which implies possible co-infection of viruses on the specific sample shown. Thus, it is difficult to associate the symptom phenotype with the detection of a single specific virus. Complete information on the individual samples can be found in Supplementary Table 1 and the description of symptoms of the associated plant hosts are summarized in Supplementary Table 9.

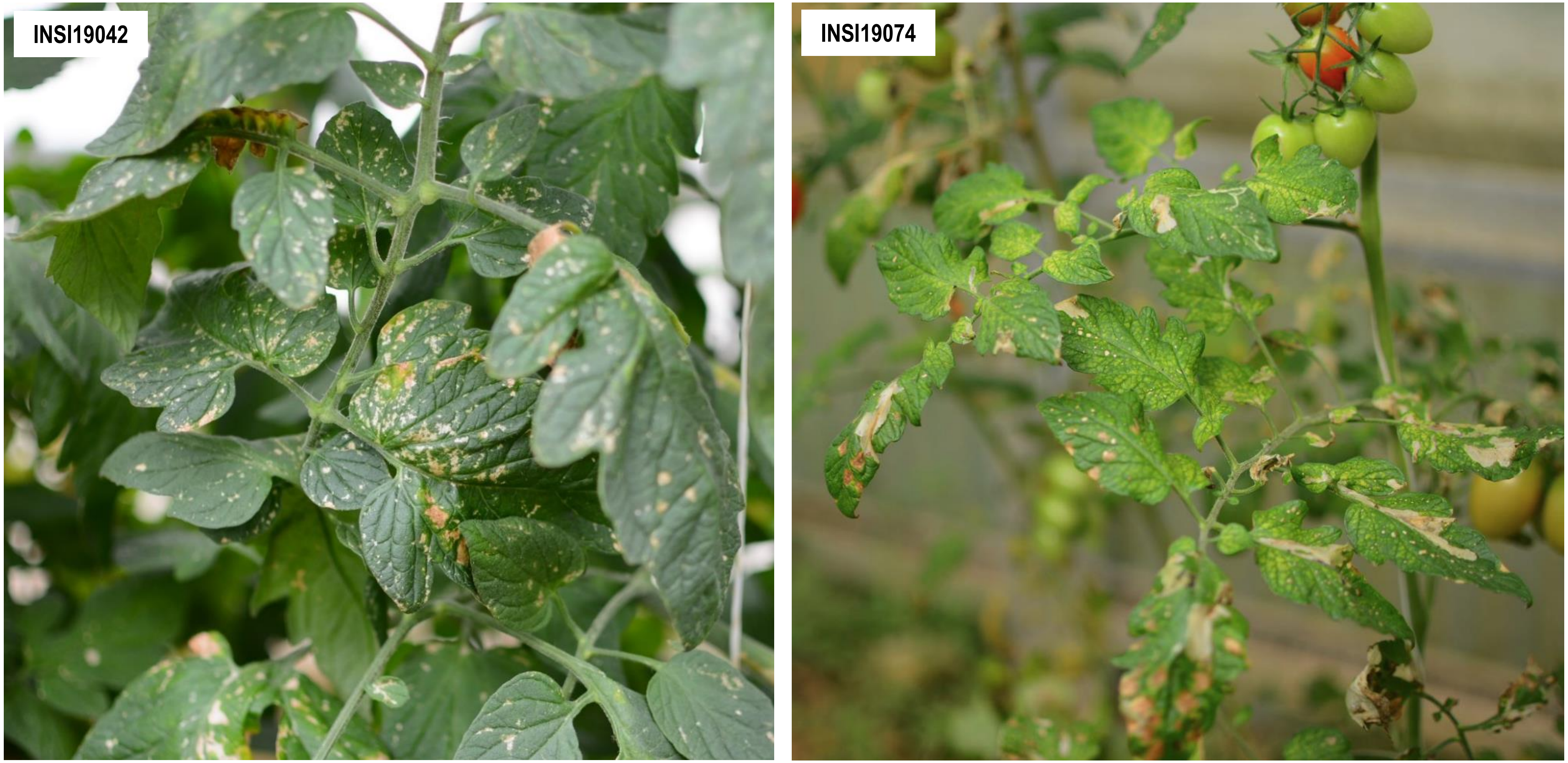

Supplementary Figure 3-16. Ranunculus white mottle ophiovirus. The confirmed associated plant hosts shown are tomatoes (*Solanum lycopersicum*).

Supplementary Figure 3 (continued)**.** Representative field photos of confirmed associated plant hosts of selected viruses and putative viroids. **Note**: There are other viruses detected in the sequencing pools (*i.e.* composite sample) where the samples shown belong, which implies possible co-infection of viruses on the specific sample shown. Thus, it is difficult to associate the symptom phenotype with the detection of a single specific virus. Complete information on the individual samples can be found in Supplementary Table 1 and the description of symptoms of the associated plant hosts are summarized in Supplementary Table 9.

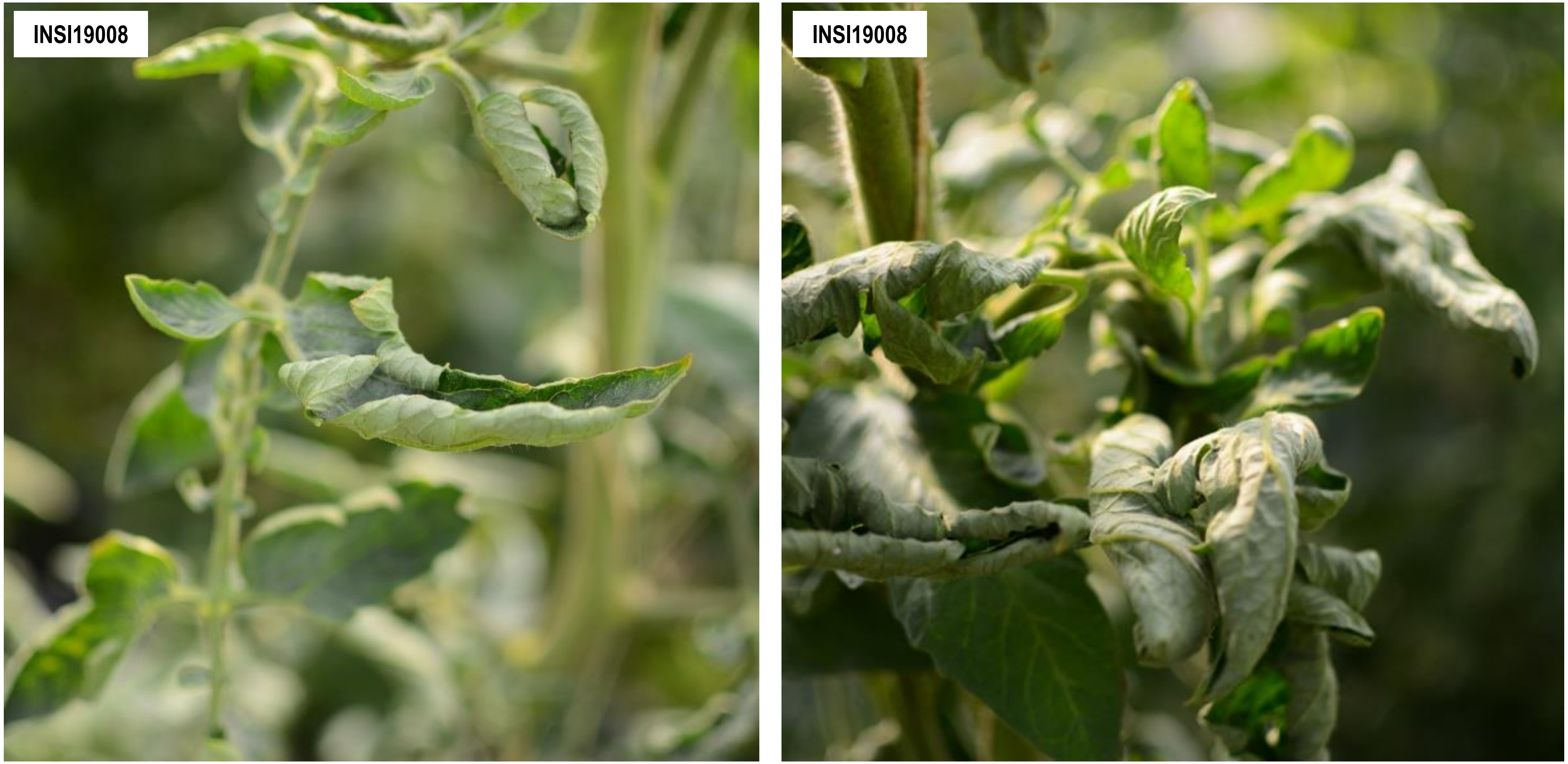

Supplementary Figure 3-17. Tomato betanucleorhabdovirus 1. The confirmed associated plant host shown is tomato (*Solanum lycopersicum*).

Supplementary Figure 3 (continued)**.** Representative field photos of confirmed associated plant hosts of selected viruses and putative viroids. **Note**: There are other viruses detected in the sequencing pools (*i.e.* composite sample) where the samples shown belong, which implies possible co-infection of viruses on the specific sample shown. Thus, it is difficult to associate the symptom phenotype with the detection of a single specific virus. Complete information on the individual samples can be found in Supplementary Table 1 and the description of symptoms of the associated plant hosts are summarized in Supplementary Table 9.

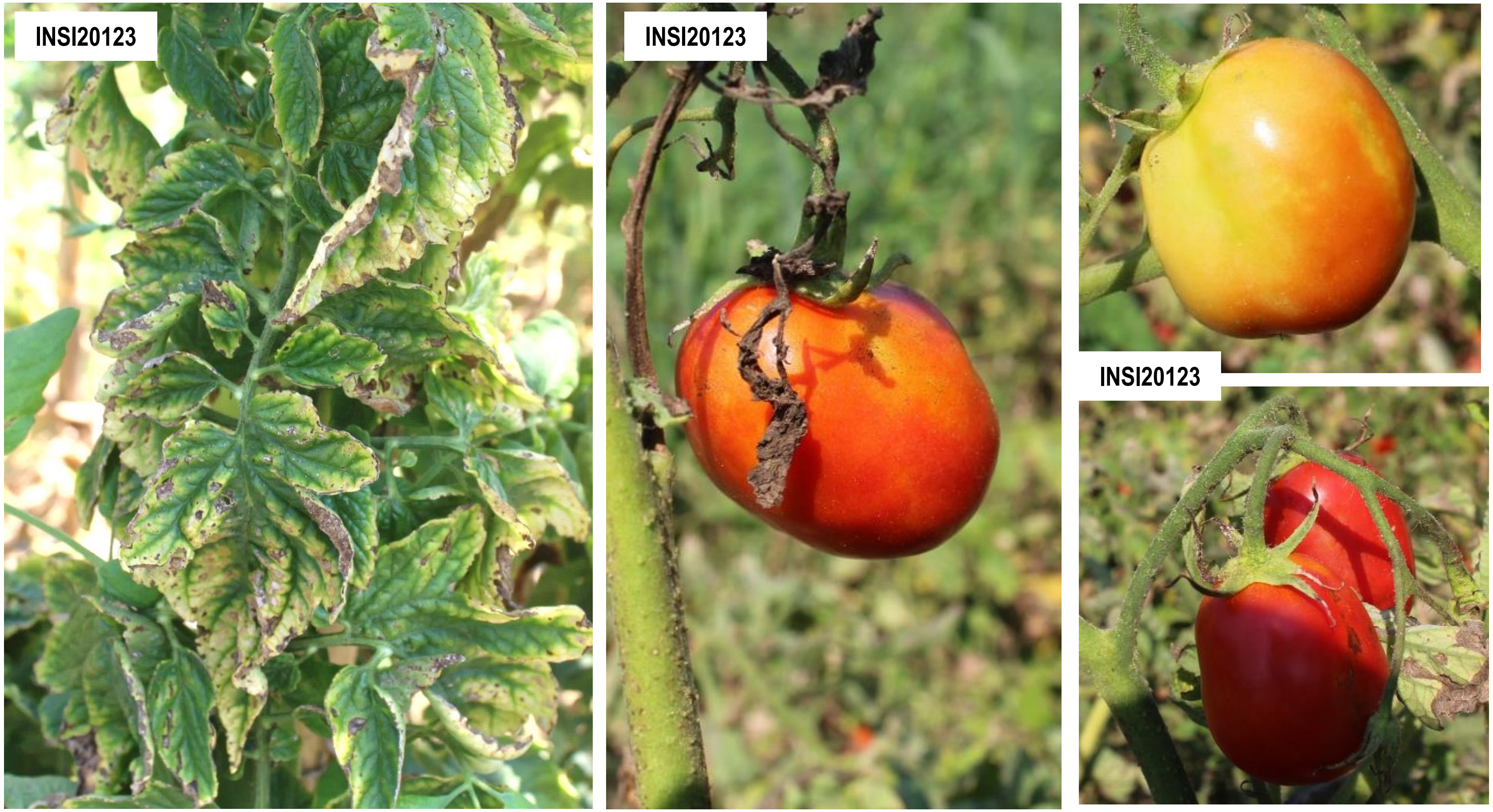

Supplementary Figure 3-18. Tomato betanucleorhabdovirus 2. The confirmed associated plant host shown is tomato (*Solanum lycopersicum*).

Supplementary Figure 3 (continued)**.** Representative field photos of confirmed associated plant hosts of selected viruses and putative viroids. **Note**: There are other viruses detected in the sequencing pools (*i.e.* composite sample) where the samples shown belong, which implies possible co-infection of viruses on the specific sample shown. Thus, it is difficult to associate the symptom phenotype with the detection of a single specific virus. Complete information on the individual samples can be found in Supplementary Table 1 and the description of symptoms of the associated plant hosts are summarized in Supplementary Table 9.

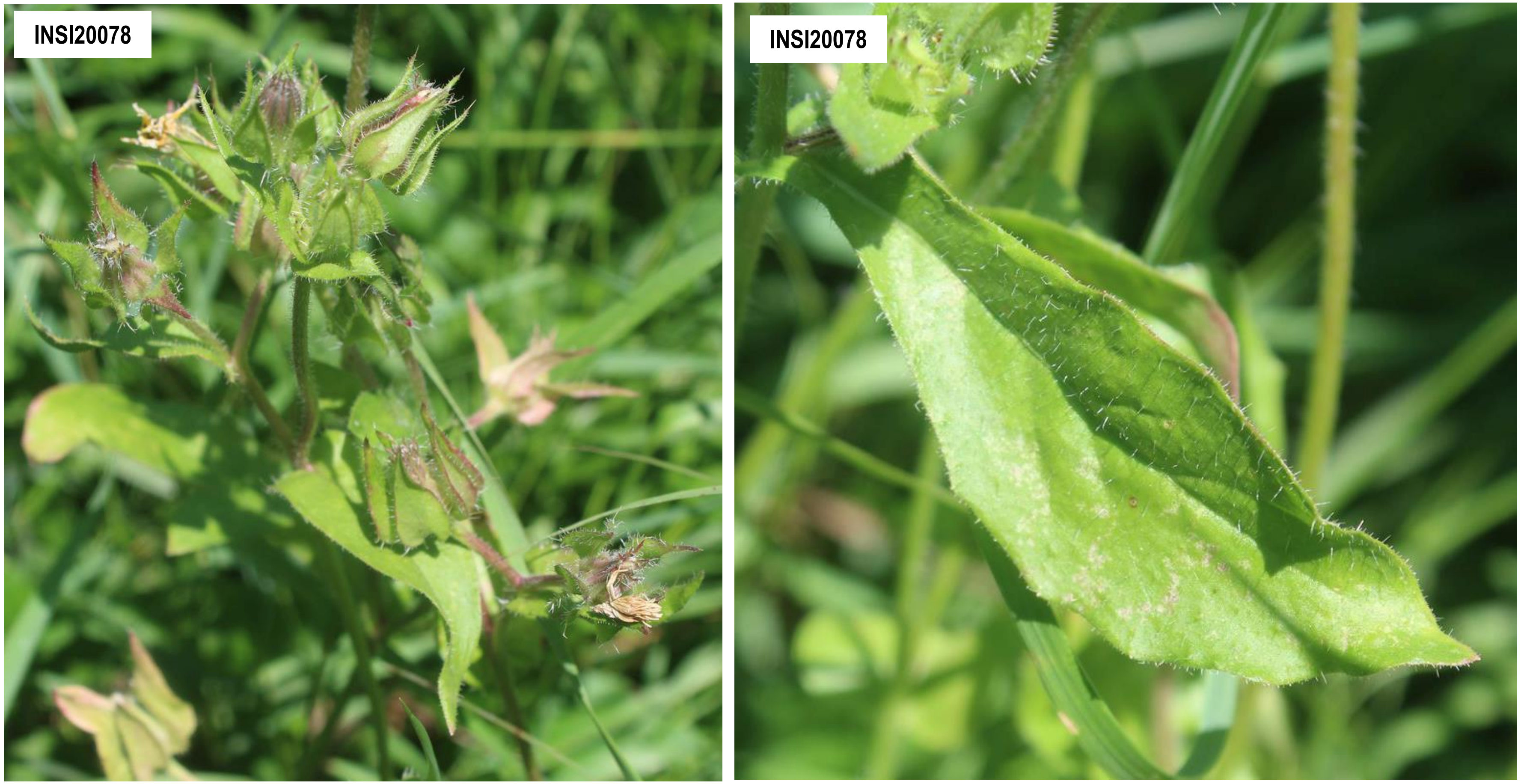

Supplementary Figure 3-19. Picris betanucleorhabdovirus 1. The confirmed associated plant host shown is *Picris echoides* (Asteraceae). The sample shown is also confirmed associated host of Cichorium alphacarmovirus 1 and Prunus virus I.

Supplementary Figure 3 (continued)**.** Representative field photos of confirmed associated plant hosts of selected viruses and putative viroids. **Note**: There are other viruses detected in the sequencing pools (*i.e.* composite sample) where the samples shown belong, which implies possible co-infection of viruses on the specific sample shown. Thus, it is difficult to associate the symptom phenotype with the detection of a single specific virus. Complete information on the individual samples can be found in Supplementary Table 1 and the description of symptoms of the associated plant hosts are summarized in Supplementary Table 9.

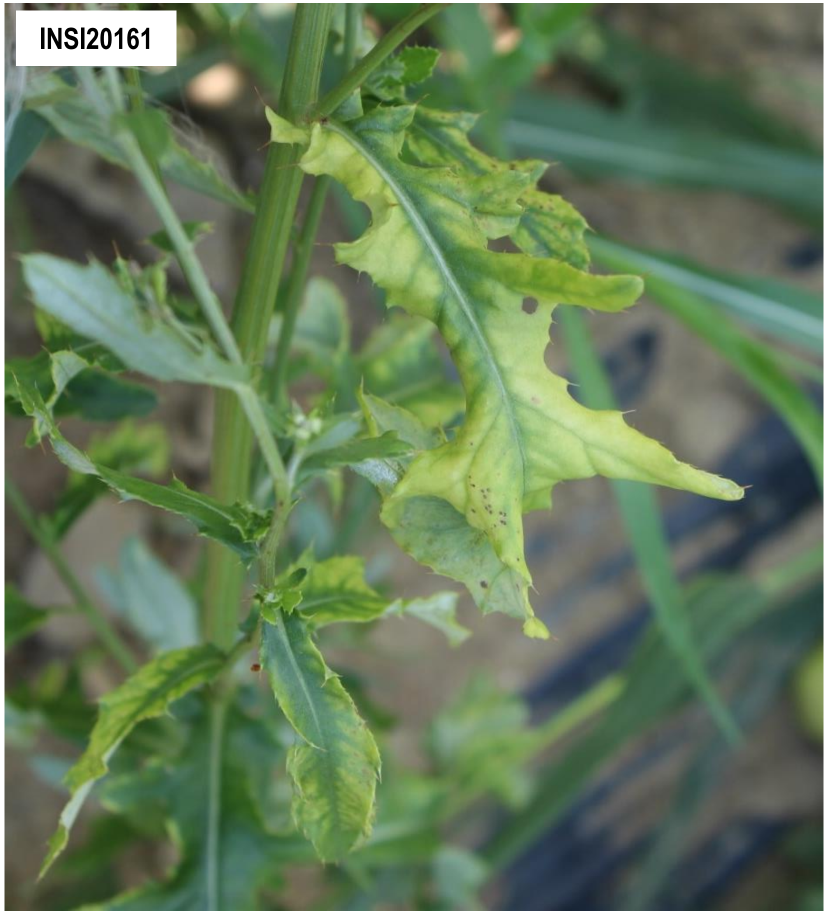

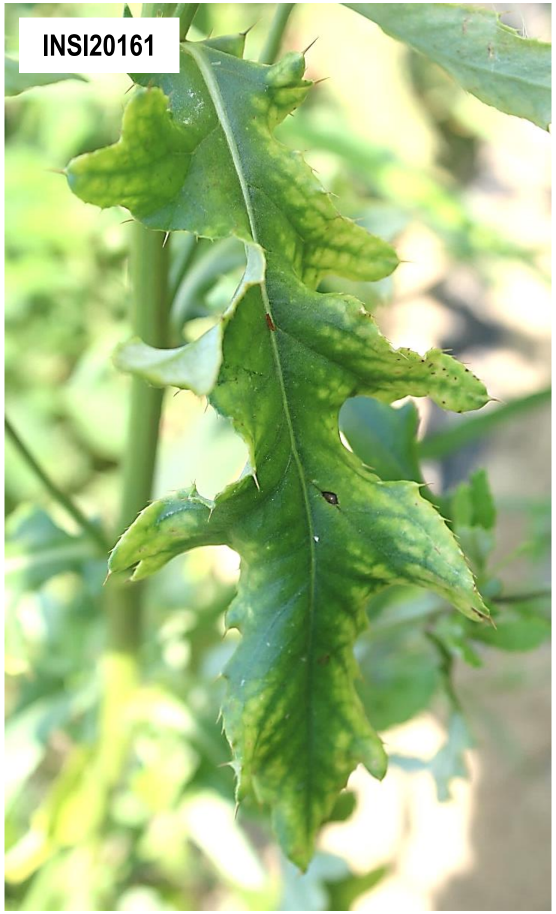

Supplementary Figure 3-20. Cirsium cytorhabdovirus 1. The confirmed associated plant host shown is *Cirsium arvense* (Asteraceae).

Supplementary Figure 3 (continued)**.** Representative field photos of confirmed associated plant hosts of selected viruses and putative viroids. **Note**: There are other viruses detected in the sequencing pools (*i.e.* composite sample) where the samples shown belong, which implies possible co-infection of viruses on the specific sample shown. Thus, it is difficult to associate the symptom phenotype with the detection of a single specific virus. Complete information on the individual samples can be found in Supplementary Table 1 and the description of symptoms of the associated plant hosts are summarized in Supplementary Table 9.

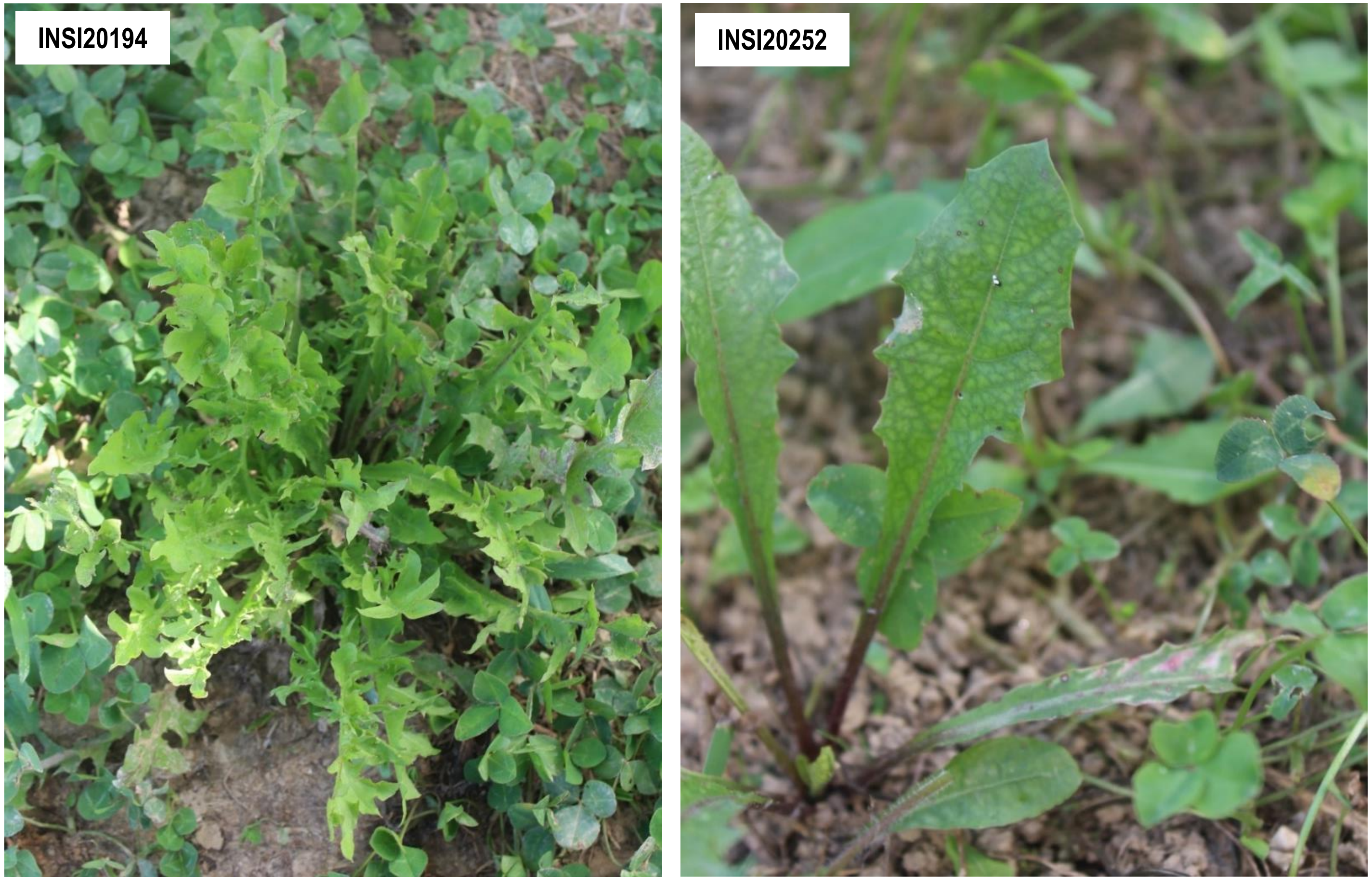

Supplementary Figure 3-21. Taraxacum betanucleorhabdovirus 1. The confirmed associated plant host shown is *Taraxacum officinale* (Asteraceae). Sample INSI20194 is also confirmed associated host of Taraxacum cytorhabdovirus 1, while sample INSI20252 is also confirmed associated host of Taraxacum viroid-like circular RNA 1.

Supplementary Figure 3 (continued)**.** Representative field photos of confirmed associated plant hosts of selected viruses and putative viroids. **Note**: There are other viruses detected in the sequencing pools (*i.e.* composite sample) where the samples shown belong, which implies possible co-infection of viruses on the specific sample shown. Thus, it is difficult to associate the symptom phenotype with the detection of a single specific virus. Complete information on the individual samples can be found in Supplementary Table 1 and the description of symptoms of the associated plant hosts are summarized in Supplementary Table 9.

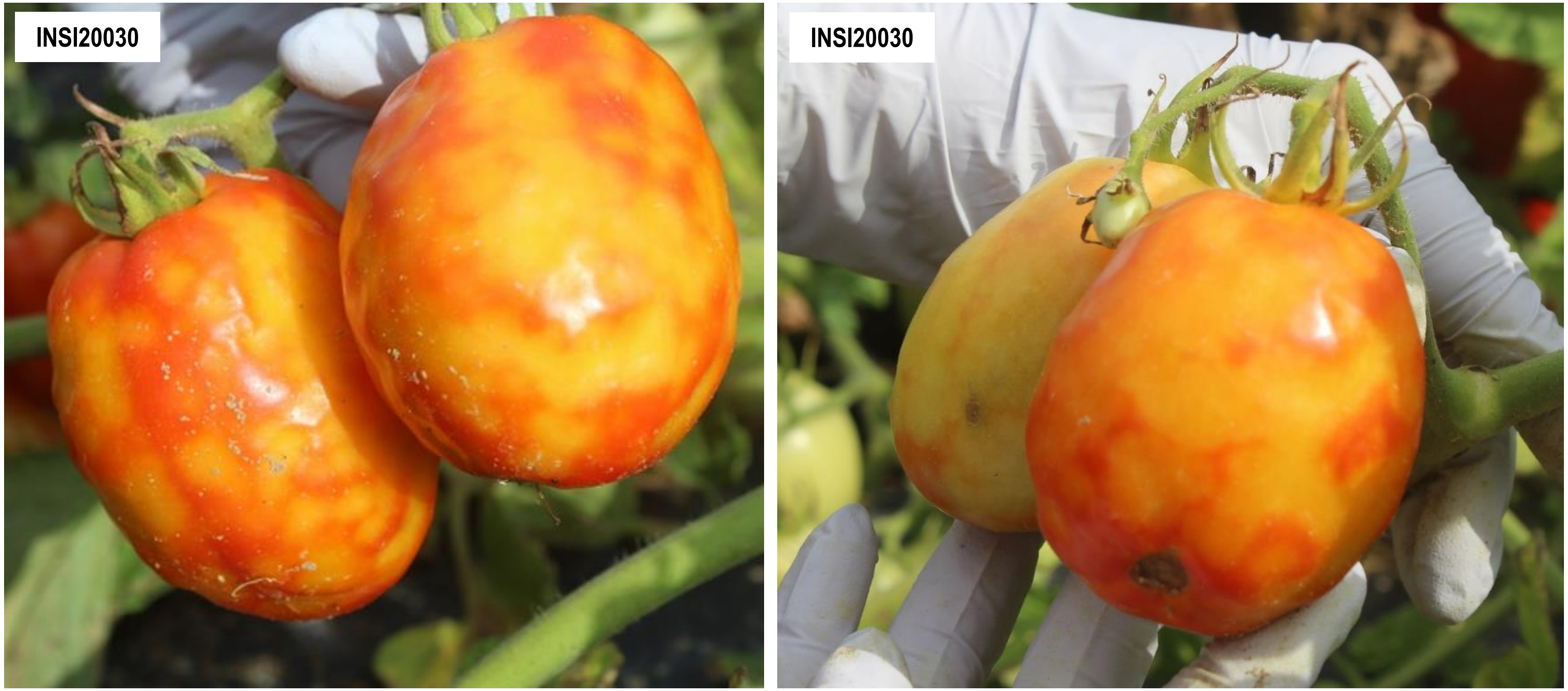

Supplementary Figure 3-22. Tomato alphanucleorhabdovirus 1. The confirmed associated plant host shown is tomato (*Solanum lycopersicum*).

Supplementary Figure 3 (continued)**.** Representative field photos of confirmed associated plant hosts of selected viruses and putative viroids. **Note**: There are other viruses detected in the sequencing pools (*i.e.* composite sample) where the samples shown belong, which implies possible co-infection of viruses on the specific sample shown. Thus, it is difficult to associate the symptom phenotype with the detection of a single specific virus. Complete information on the individual samples can be found in Supplementary Table 1 and the description of symptoms of the associated plant hosts are summarized in Supplementary Table 9.

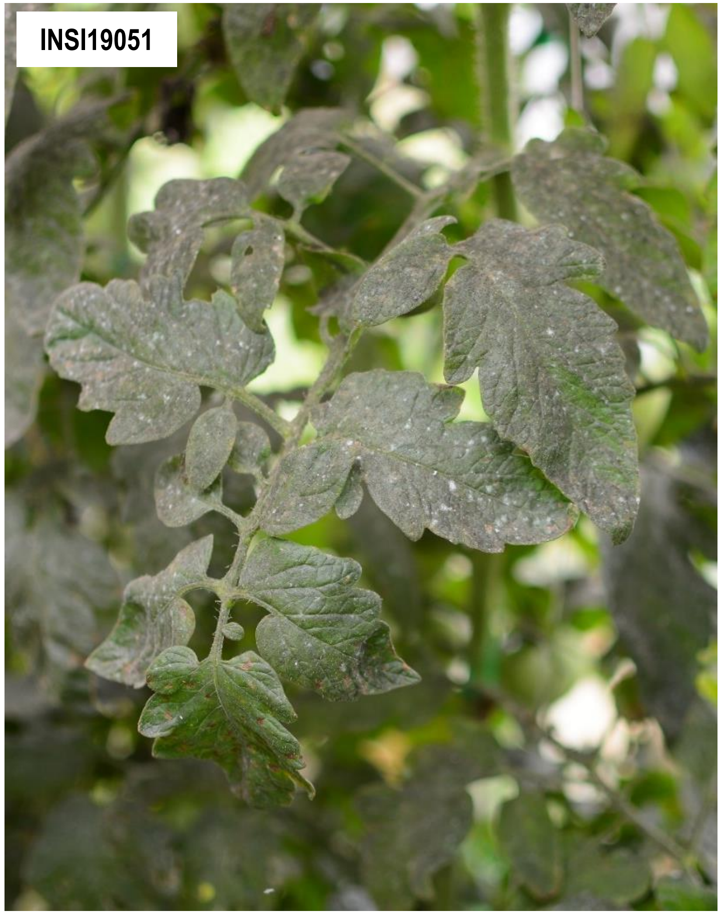

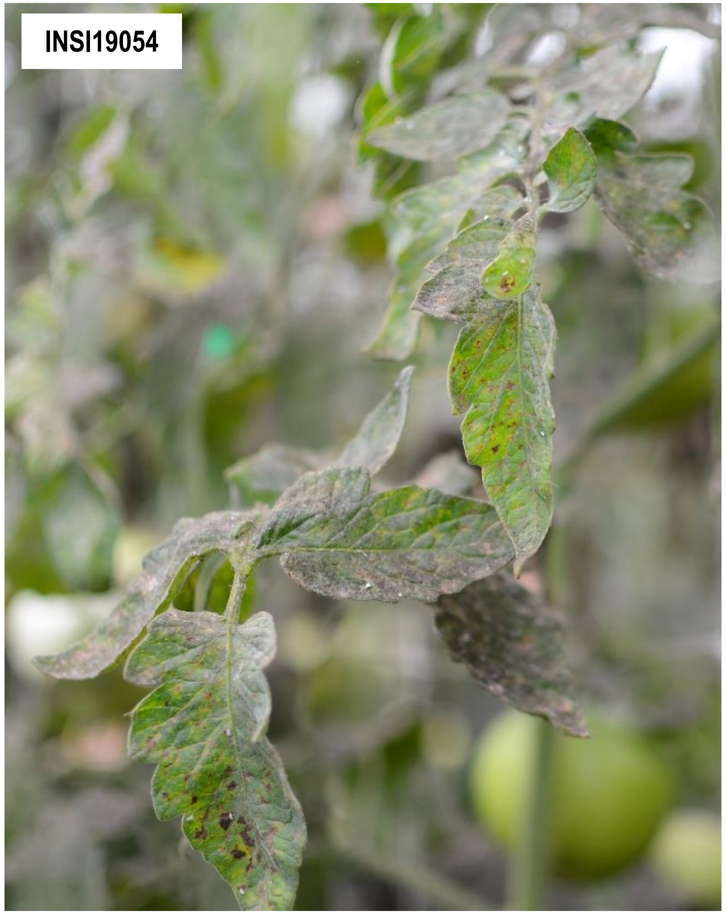

Supplementary Figure 3-23. Leveillula taurica associated rhabdo-like virus 1. The confirmed associated plant host shown is *Solanum lycopersicum* (Solanaceae).

Supplementary Figure 3 (continued)**.** Representative field photos of confirmed associated plant hosts of selected viruses and putative viroids. **Note**: There are other viruses detected in the sequencing pools (*i.e.* composite sample) where the samples shown belong, which implies possible co-infection of viruses on the specific sample shown. Thus, it is difficult to associate the symptom phenotype with the detection of a single specific virus. Complete information on the individual samples can be found in Supplementary Table 1 and the description of symptoms of the associated plant hosts are summarized in Supplementary Table 9.

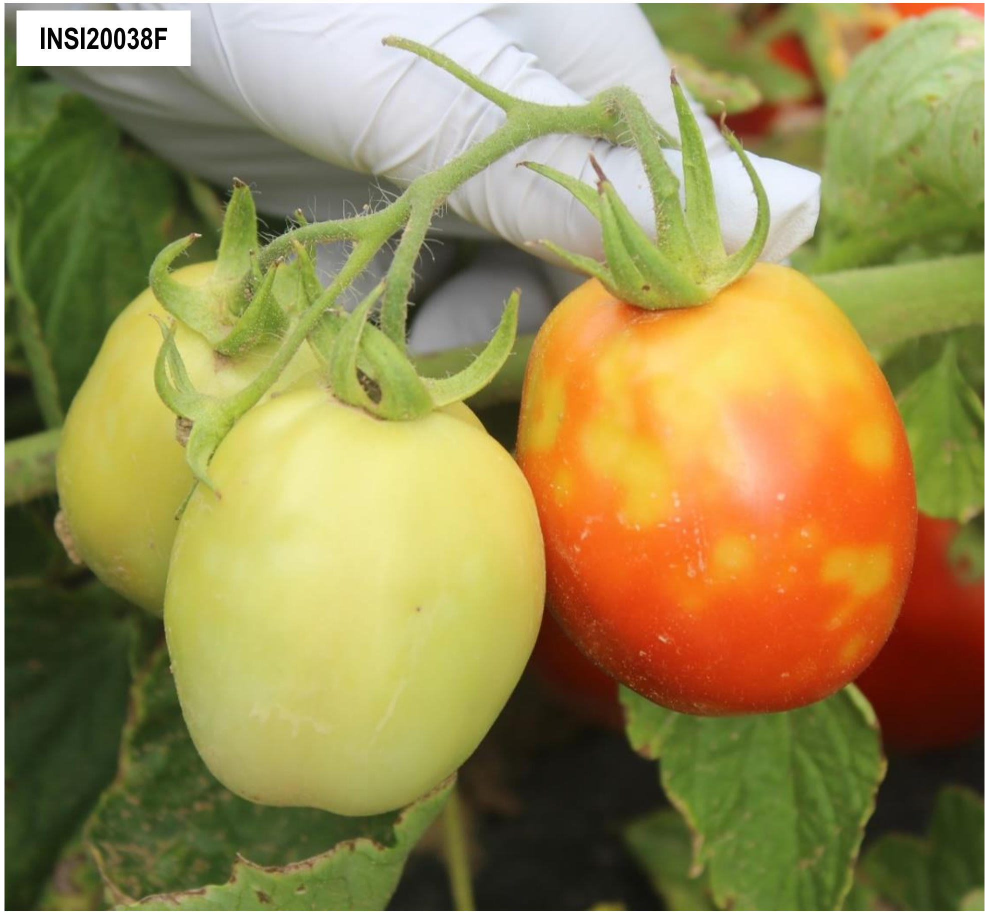

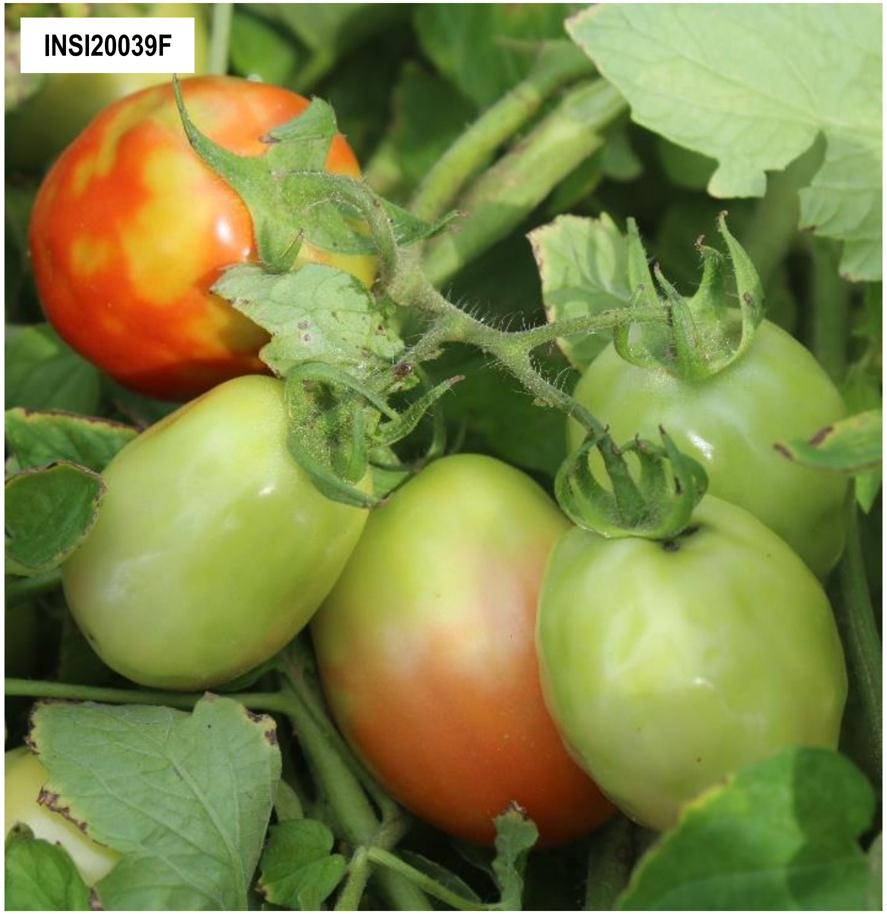

Supplementary Figure 3-24. Eggplant mottled dwarf alphanucleorhabdovirus. The confirmed associated plant host shown is *Solanum lycopersicum* (Solanaceae).

Supplementary Figure 3 (continued)**.** Representative field photos of confirmed associated plant hosts of selected viruses and putative viroids. **Note**: There are other viruses detected in the sequencing pools (*i.e.* composite sample) where the samples shown belong, which implies possible co-infection of viruses on the specific sample shown. Thus, it is difficult to associate the symptom phenotype with the detection of a single specific virus. Complete information on the individual samples can be found in Supplementary Table 1 and the description of symptoms of the associated plant hosts are summarized in Supplementary Table 9.

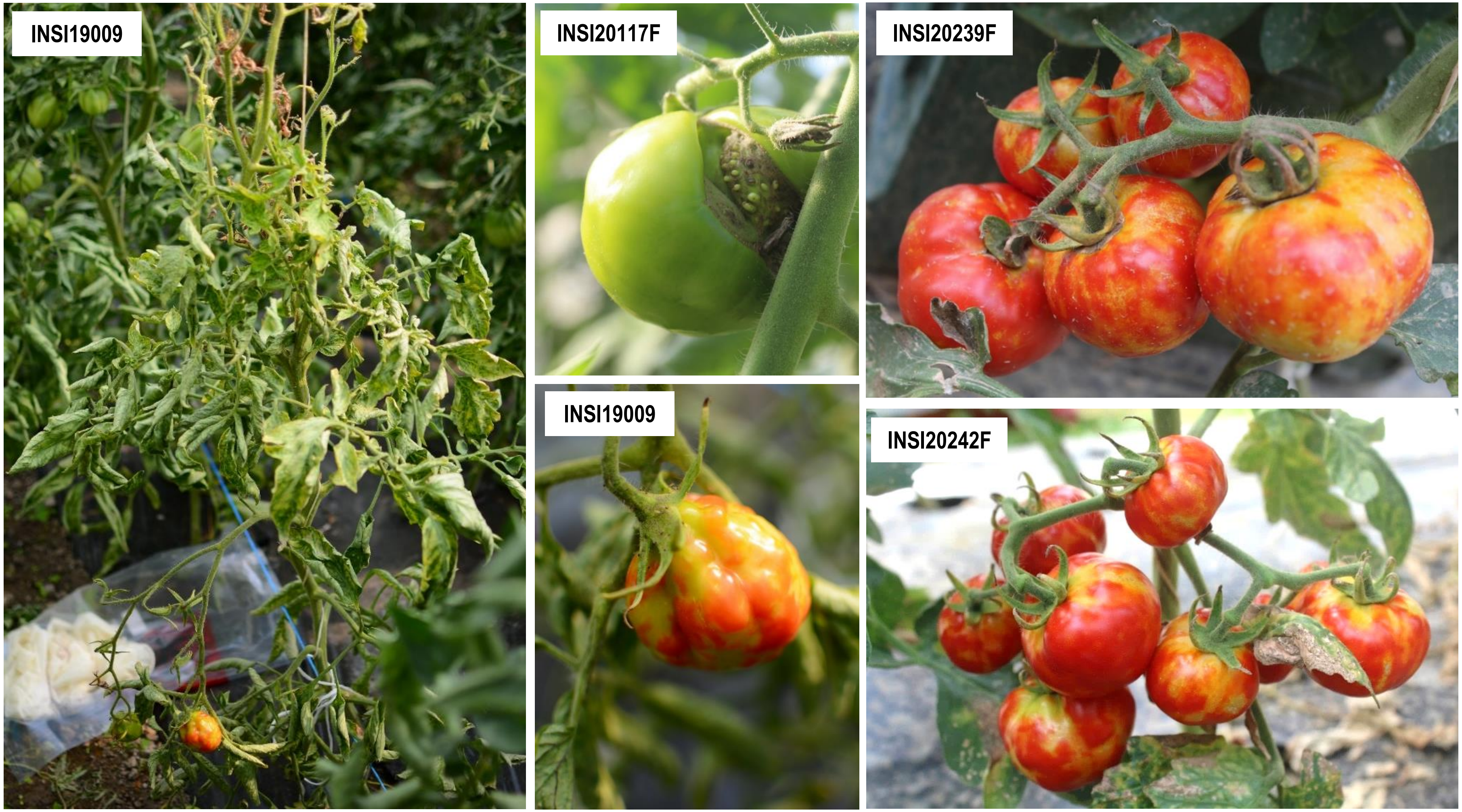

Supplementary Figure 3-25. Physostegia chlorotic mottle alphanucleorhabdovirus. All confirmed associated plant hosts shown are tomatoes (*Solanum lycopersicum*).

Supplementary Figure 3 (continued)**.** Representative field photos of confirmed associated plant hosts of selected viruses and putative viroids. **Note**: There are other viruses detected in the sequencing pools (*i.e.* composite sample) where the samples shown belong, which implies possible co-infection of viruses on the specific sample shown. Thus, it is difficult to associate the symptom phenotype with the detection of a single specific virus. Complete information on the individual samples can be found in Supplementary Table 1 and the description of symptoms of the associated plant hosts are summarized in Supplementary Table 9.

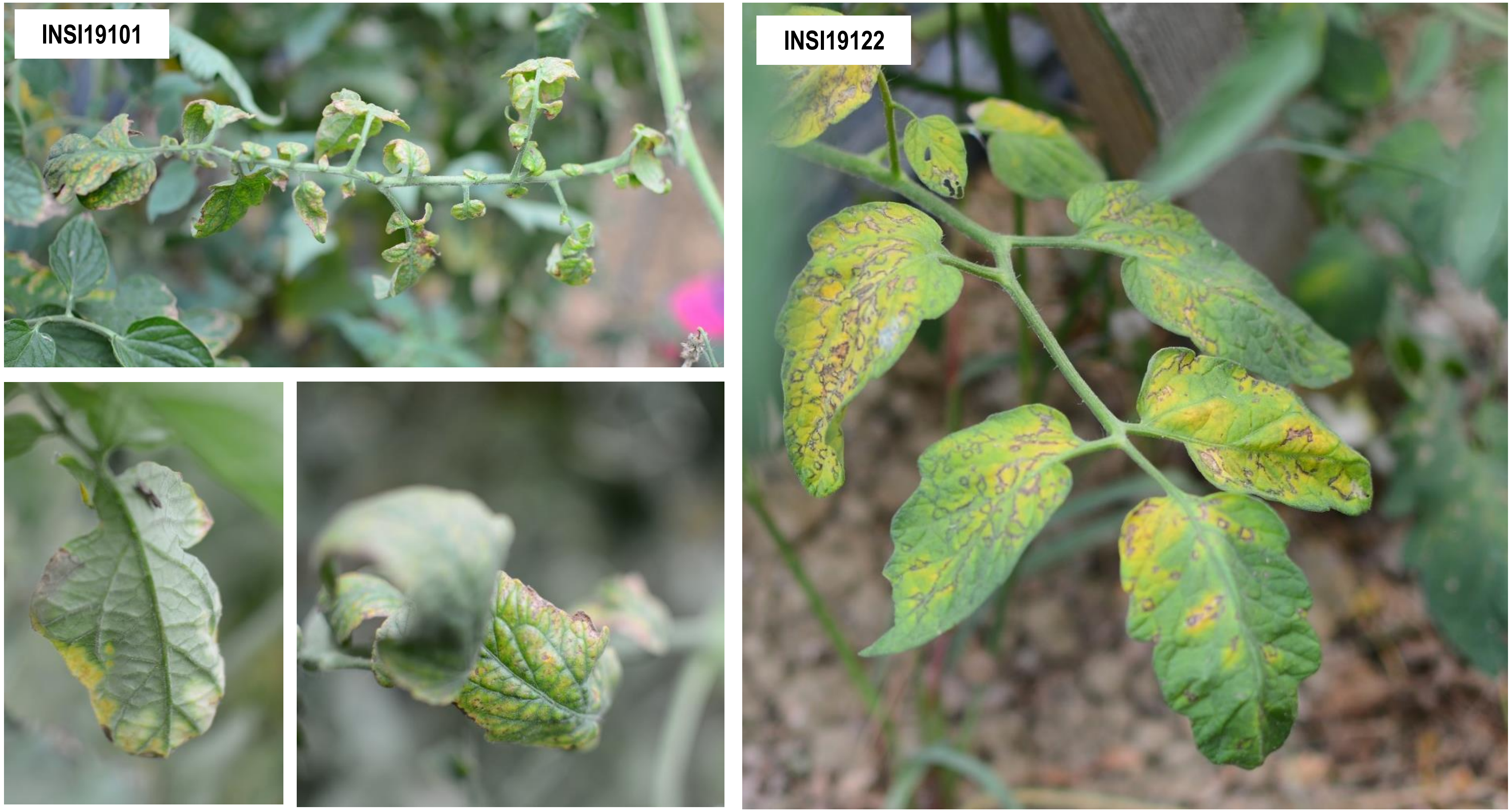

Supplementary Figure 3-26. Tomato fruit blotch virus and Tomato matilda virus co-infected samples.
The confirmed associated plant host shown is *Solanum lycopersicum* (Solanaceae).

Supplementary Figure 3 (continued)**.** Representative field photos of confirmed associated plant hosts of selected viruses and putative viroids. **Note**: There are other viruses detected in the sequencing pools (*i.e.* composite sample) where the samples shown belong, which implies possible co-infection of viruses on the specific sample shown. Thus, it is difficult to associate the symptom phenotype with the detection of a single specific virus. Complete information on the individual samples can be found in Supplementary Table 1 and the description of symptoms of the associated plant hosts are summarized in Supplementary Table 9.

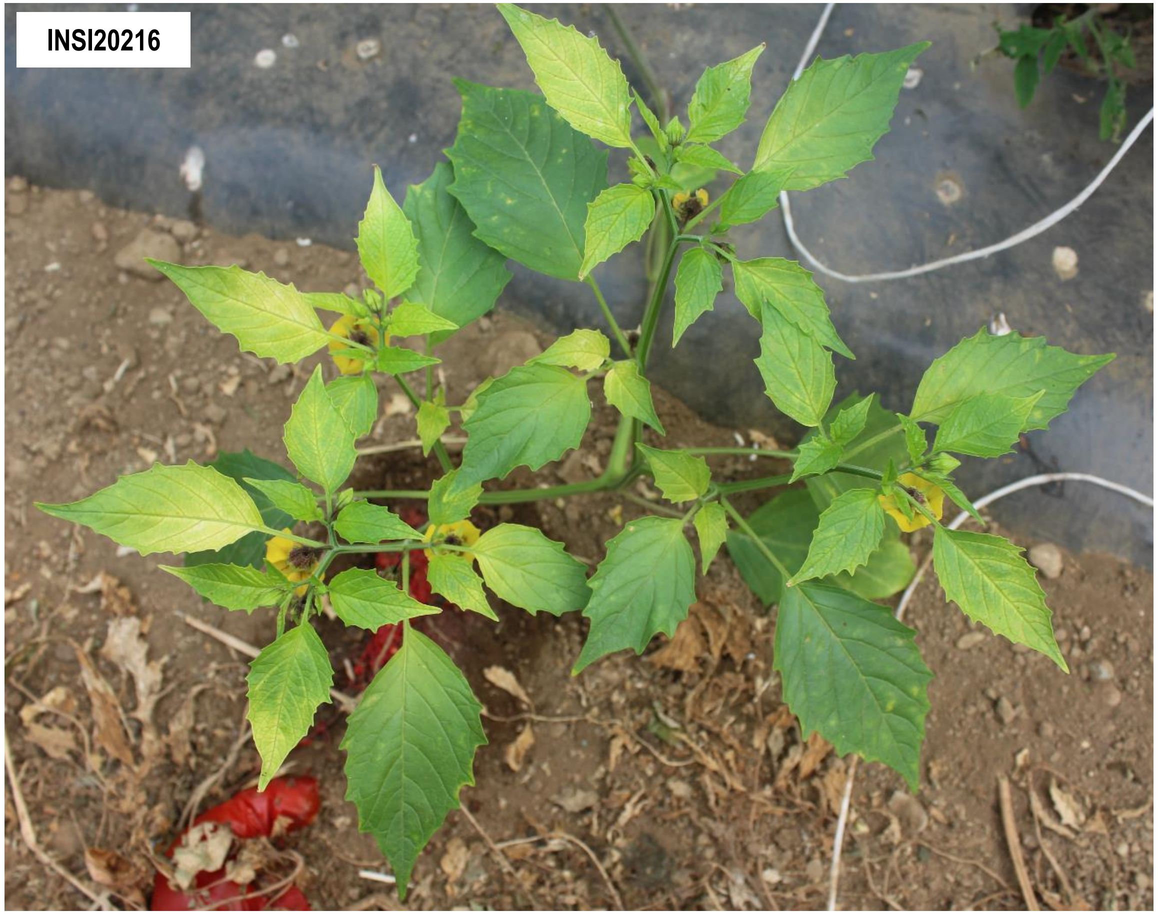

Supplementary Figure 3-27. Solanum nigrum ilarvirus 1. The confirmed associated plant host shown is *Physalis* sp. (Solanaceae).

Supplementary Figure 3 (continued)**.** Representative field photos of confirmed associated plant hosts of selected viruses and putative viroids. **Note**: There are other viruses detected in the sequencing pools (*i.e.* composite sample) where the samples shown belong, which implies possible co-infection of viruses on the specific sample shown. Thus, it is difficult to associate the symptom phenotype with the detection of a single specific virus. Complete information on the individual samples can be found in Supplementary Table 1 and the description of symptoms of the associated plant hosts are summarized in Supplementary Table 9.
