# Supplementary material for: In-depth study of tomato and weed viromes reveals undiscovered plant virus diversity in an agroecosystem
Source: Microbiome. 2023 Mar 28;11:60. doi: 10.1186/s40168-023-01500-6 (PMC10042675; doi:10.1186/s40168-023-01500-6)
Supplement: Supplementary file 11 — Additional file 10: Supplementary Figure 5. RT-PCR, TEM and nanopore sequencing results on the confirmation of infectivity of Plantago tobamovirus 1 in solanaceous hosts. [file 40168_2023_1500_MOESM10_ESM.pdf]

In-depth study of tomato and weed viromes reveals undiscovered plant virus diversity in an agroecosystem

Rivarez, MPS[1,2,*], Pecman, A[1,2], Bačnik, K[1,2], Maksimović Carvalho Ferreira, O[1,2], Vučurović, A[1], Seljak, G[1], Mehle, N[1,3], Gutiérrez-Aguirre, I[1], Ravnikar, M[1], and Kutnjak, D[1,*]

[1]Department of Biotechnology and Systems Biology, National Institute of Biology, Večna pot 111, Ljubljana, 1000 Slovenia. [2]Jožef Stefan International Postgraduate School, Jamova cesta 39, Ljubljana, 1000 Slovenia. [3]School for Viticulture and Enology, University of Nova Gorica, Dvorec Lanthieri Glavni trg 8, Vipava, 5271 Slovenia. *for correspondence email Denis Kutnjak (denis.kutnjak@nib.si) and Mark Paul Selda Rivarez (mpsrivarez@gmail.com)

SUPPLEMENTARY INFORMATION

Additional File 10

Supplementary Figure 5. RT-PCR, TEM and nanopore sequencing results on the confirmation of infectivity of Plantago tobamovirus 1 (PTV1) on Solanaceae hosts.

a

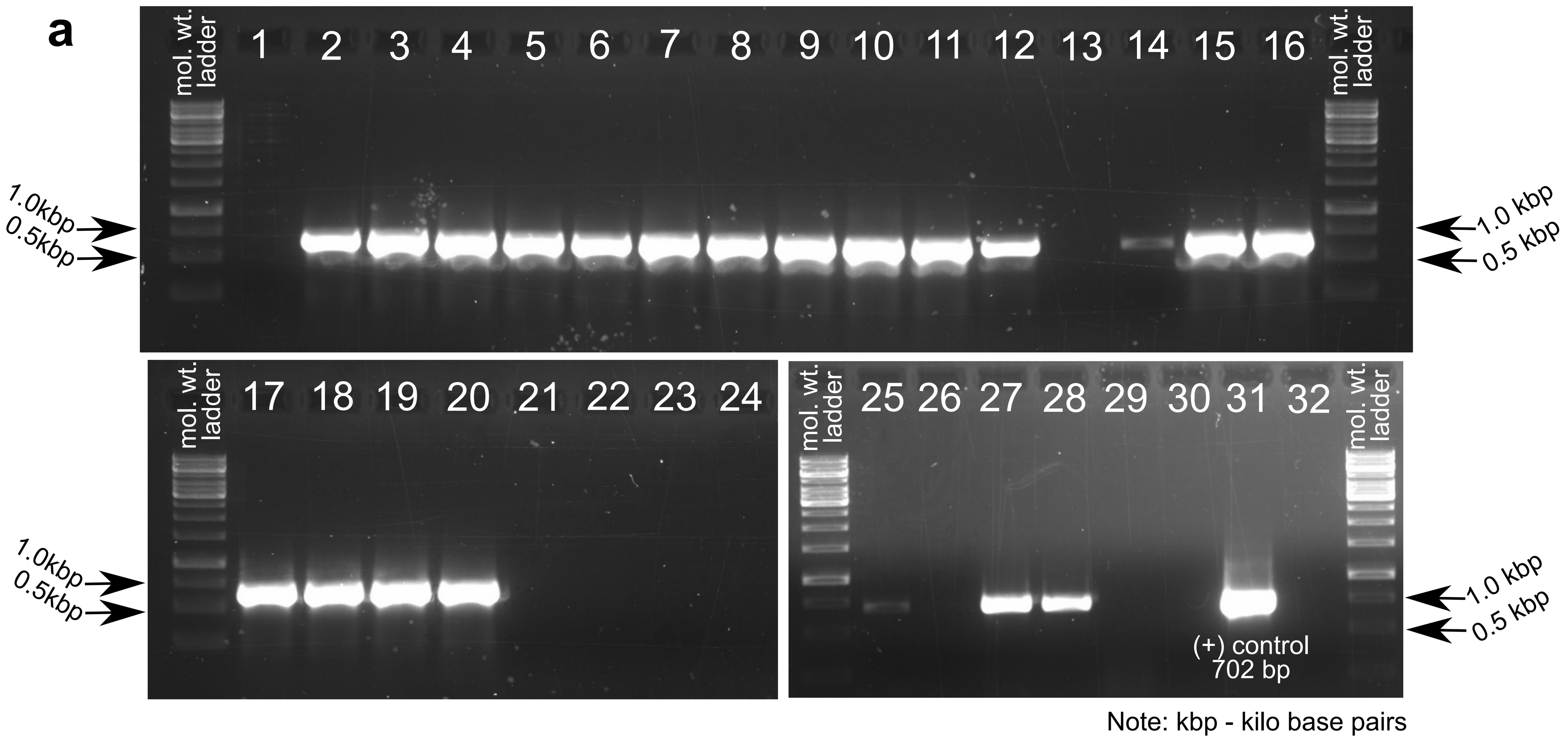

b

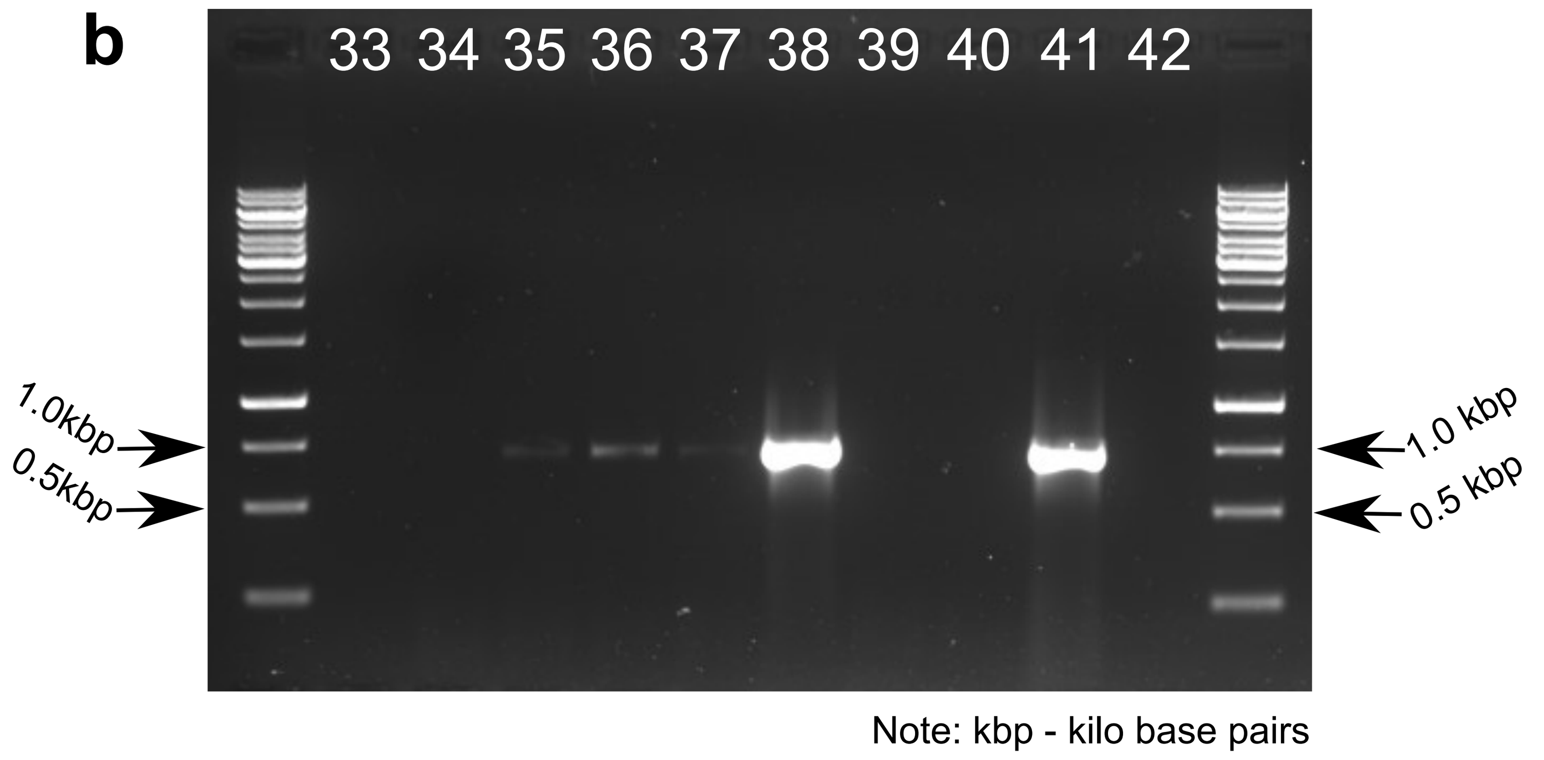

Samples tested in the RT-PCR assay shown in **a:**

Assay controls in **a**:

1 - non-template control 1
13 - blank 1
21 - negative control of RNA isolation 1
22 - negative control of RNA isolation 2
23 - non-template control 1
24 - blank 2
31 - positive control (PTV1 RNA)
32 - negative control of RNA isolation 3

Inoculated plant	7dpi pool of inoculated leaves	14 dpi pool of inoculated leaves	14 dpi pool of young apical (systemic) leaves	21 dpi pool of young apical (systemic) leaves	28 dpi pool of young apical (systemic) leaves	35 dpi pool of young apical (systemic) leaves
Solanum lycopersicum	2[asym]	8[asym]	14[asym]	25[a,asym] 26[b,asym]	27[a,asym] 28[b,asym]	29[a,asym] 30[b,asym]
Nicotiana benthamiana	3[sym]	9[sym]	15[sym]	17[sym]	19[sym]	not tested
Nicotiana clevelandii	4[sym]	10[sym]	16[sym]	18[sym]	20[sym,HTS(+)]	not tested

Note: dpi - days post inoculation, [a]10x diluted RNA, [b]100x diluted RNA, [asym]asymptomatic, [sym]symptomatic, [HTS(+)]was used in HTS where presence of PTV1 was confirmed. Other inoculated plants tested: 7dpi/14dpi pool of inoculated leaves: *Nicotiana tabacum* (lane 5/lane11), *Nicotiana glutinosa* (lane 6/lane12), *Chenopodium quinoa* (lane 7/-);

Samples tested in the RT-PCR assay shown in **b:**

Assay controls in **b**:

33 - non-template control
41 - positive control (PTV1 RNA)
42 - negative control of RNA isolation

Inoculated plant	28 dpi young apical (systemic) leaves from individual plants					49 dpi pool of young apical (systemic) leaves	56 dpi pool of young apical (systemic) leaves
	plant 1	plant 2	plant 3	plant 4	plant 5		
Solanum lycopersicum	34[asym]	35[asym]	36[asym]	37[asym]	38[asym]	39[asym]	40[asym]

Note: RNA extracts used in this assay (**b**) were diluted 1:200, except for the positive control. dpi - days post inoculation. [asym]asymptomatic. [sym]symptomatic.

c

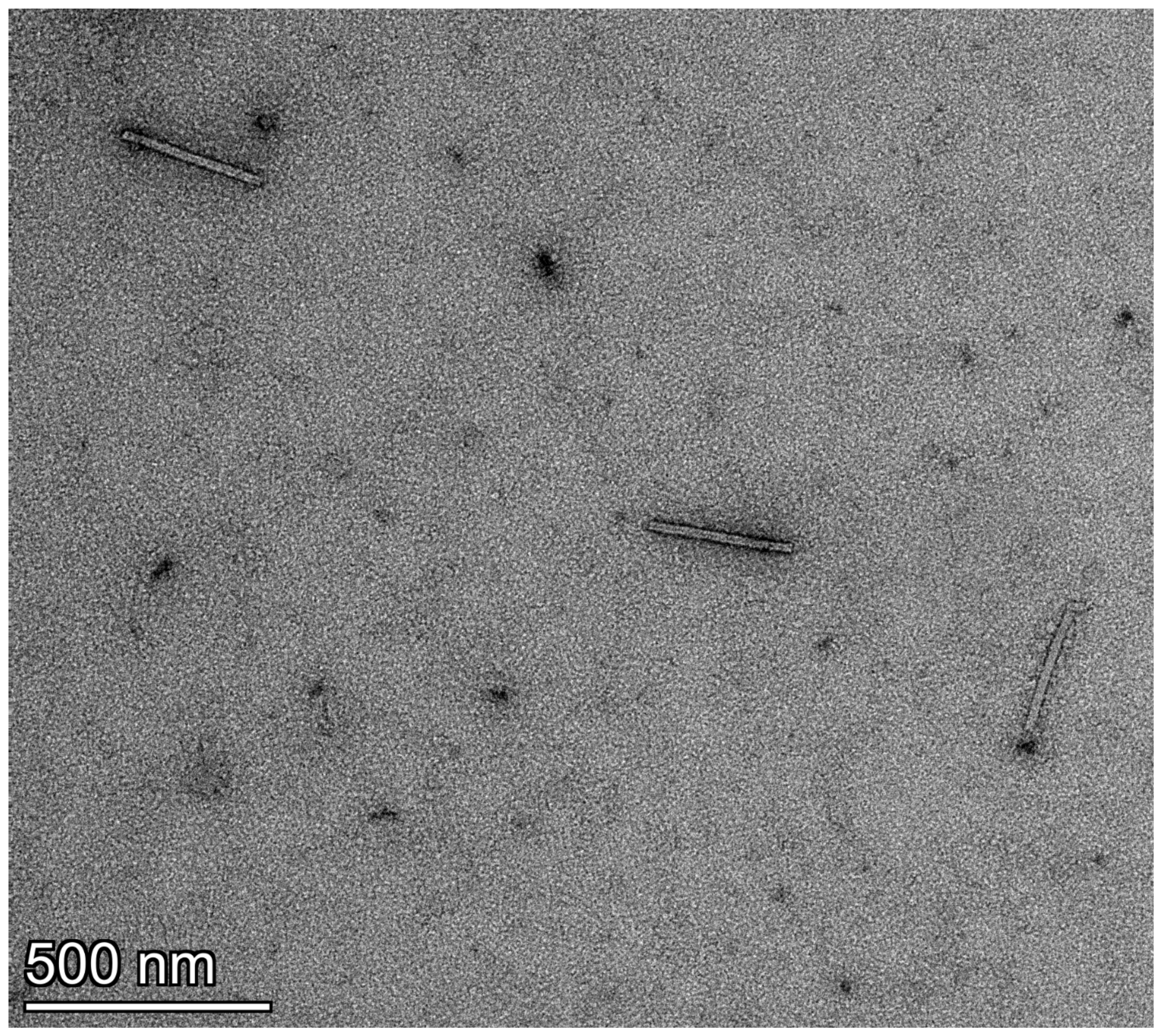

d

Test plant / collection dpi / individual or pooled?	No. of quality-controlled (QC) reads	No. of reads mapping to PTV1 genome	Percentage of QC reads mapping to PTV1 genome
Solanum lycopersicum / 28 dpi / individual plant[a]	157191	0	0.00%
Solanum lycopersicum / 28 dpi / individual plant[b]	160438	0	0.00%
Nicotiana clevelandii / 21 dpi / pooled plants[c]	159570	14216	8.91%

Note: [a]lane 35 in (**b**). [b]lane 37 in (**b**). [c]lane 18 in (**a**).

Supplementary Figure 5. RT-PCR, TEM and nanopore sequencing results on the confirmation of infectivity of Plantago tobamovirus 1 (PTV1) on Solanaceae hosts. **a, b** RT-PCR assays for the detection of PTV1 in inoculated and young apical (systemic) leaves at different time points. **c** Transmission electron micrograph showing three virions of PTV1 from crude preparations of infected *Nicotiana clevelandii* tissues. **d** Number and proportion of long reads mapping to PTV1 genome, derived from selected inoculated plants.
