# Supplementary material for: In-depth study of tomato and weed viromes reveals undiscovered plant virus diversity in an agroecosystem
Source: Microbiome. 2023 Mar 28;11:60. doi: 10.1186/s40168-023-01500-6 (PMC10042675; doi:10.1186/s40168-023-01500-6)
Supplement: Supplementary file 12 — Additional file 11. Detailed parameters of the pipeline used for screening and detection of virus sequences in analyzed datasets implemented in CLC-GWB v. 20. [file 40168_2023_1500_MOESM11_ESM.pdf]

Detailed parameters of the pipeline used for screening and detection of virus sequences in analyzed datasets implemented in CLC-GWB v 20

The pipeline is freely available at: https://gitlab.com/ilvo/phbn-wp2-training/-/tree/master/CLC_NIB_1, where also the detailed how-to manual for its usage is provided.

Illumina reads were analyzed using a pipeline in CLC Genomic Workbench (v20). Quality control was performed, then, adapters were removed from all reads, and additionally, reads were trimmed by quality (quality limit = 0.05) and length (all reads shorter than 30 nts were discarded). Trimmed reads were mapped to viral RefSeq (NCBI database, Jul 2020) and de novo assembled, according to the parameters provided in tables below. Contigs (longer than 100 nts) were also mapped to viral RefSeq (NCBI database, Jul 2020) and unmapped contigs were translated to protein sequences and further analyzed by searching for conserved protein domains using Pfam analysis (v33), according to parameters provided in tables below.

Parameters used in CLC Genomic Workbench 20 for mapping reads to viral/viroid genomes

**Mapping reads**	
Masking mode	No masking
Masking track	
Match score	1
Mismatch cost	1
Cost of insertions and deletions	Linear gap cost
Insertion cost	2
Deletion cost	2
Length fraction	0.8
Similarity fraction	0.8
Global alignment	false
Auto-detect paired distances	false
Non-specific match handling	Map randomly

Parameters used in CLC Genomic Workbench 20 for de novo assembly

**De Novo Assembly**	
Mapping mode	Create simple contig sequences (fast)
Automatic bubble size	true
Automatic word size	true
Minimum contig length	100
Guidance only reads	false
Perform scaffolding	true
Auto-detect paired distances	true

Parameters used in CLC Genomic Workbench 20 for mapping contigs to viral/viroid genomes

**Mapping contigs**	Diagnostic workflow
Masking mode	No masking
Masking track	
Match score	1
Mismatch cost	1
Cost of insertions and deletions	Linear gap cost
Insertion cost	2
Deletion cost	2
Length fraction	0.7
Similarity fraction	0.7
Global alignment	false
Auto-detect paired distances	false
Non-specific match handling	Map randomly

Parameters used in CLC Genomic Workbench 12 for Pfam domain search

**Translate to Protein**	
Genetic code	1 Standard
Reading frame	+1, +2, +3, -1, -2, -3
**Pfam Domain Search**	
Database	Pfam-A v33
Use profile's gathering cutoffs	true
Remove overlapping matches from the same clan	true
